# Smac mimetic combined with eCD4-Ig reverses latency without reducing SHIV reservoirs in rhesus macaques

**DOI:** 10.1172/JCI187961

**Published:** 2026-03-16

**Authors:** Lars Pache, John K. Bui, Lindsay M. Klouser, Christine M. Fennessey, Alexander C. Noyola, Teresa Einhaus, Haiying Zhu, Laurence Stensland, Isai Leguizamo, Abubakarr A. Koroma, Peter Teriete, W.L. William Chang, Ollivier Hyrien, Natasha N. Duggan, Dominik Heimann, Ailyn C. Pérez-Osorio, Katharine J. Bar, Nicholas D.P. Cosford, Brandon F. Keele, Dennis J. Hartigan-O’Connor, Michael Farzan, Matthew R. Gardner, Keith R. Jerome, Sumit K. Chanda, Hans-Peter Kiem, Christopher W. Peterson

**Affiliations:** 1 Center for Therapeutics Discovery, NCI Designated Cancer Center, Sanford Burnham Prebys Medical Discovery Institute, La Jolla, California, USA.; 2 Translational Science and Therapeutics Division, Fred Hutchinson Cancer Center, Seattle, Washington, USA.; 3 Department of Medicine and; 4 Department of Laboratory Medicine and Pathology, University of Washington, Seattle, Washington, USA.; 5 AIDS and Cancer Virus Program, Frederick National Laboratory for Cancer Research, Frederick, Maryland, USA.; 6 Division of Microbiology and Immunology, Emory National Primate Research Center, Emory University, Atlanta, Georgia, USA.; 7 California National Primate Research Center, University of California, Davis, Davis, California, USA.; 8 Department of Medical Microbiology and Immunology, School of Medicine, University of California, Davis, Davis, California, USA.; 9 Biostatistics, Bioinformatics and Epidemiology Program, Vaccine and Infectious Disease Division, Fred Hutchinson Cancer Center, Seattle, Washington, USA.; 10 Department of Immunology and Microbiology, Scripps Research, La Jolla, California, USA.; 11 Department of Medicine, Perelman School of Medicine, University of Pennsylvania, Philadelphia, Pennsylvania, USA.; 12 Division of Experimental Medicine, Department of Medicine, University of California, San Francisco, San Francisco, California, USA.; 13 Department of Pediatrics, Boston Children’s Hospital, Harvard Medical School, Boston, Massachusetts, USA.; 14 The Center for Integrated Solutions to Infectious Diseases (CISID), The Broad Institute of MIT and Harvard, Cambridge, Massachusetts, USA.; 15 Department of Medicine, Division of Infectious Diseases, Emory University, Atlanta, Georgia, USA.; 16 Vaccine and Infectious Disease Division, Fred Hutchinson Cancer Center, Seattle, Washington, USA.

**Keywords:** AIDS/HIV, Infectious disease, Virology, Drug therapy

## Abstract

Despite the success of antiretroviral therapy in controlling HIV replication, latent viral reservoirs persist, presenting a major barrier to a cure. Current treatment approaches that aim to reactivate latent virus and eliminate infected cells, termed “shock and kill,” hold promise but have yet to demonstrate meaningful reservoir reduction in vivo. In this study, we explored combining ciapavir, a Smac mimetic latency-reversing agent, with adeno-associated virus–delivered (AAV-delivered) eCD4-Ig to treat antiretroviral therapy–suppressed, SHIV-infected rhesus macaques. We could demonstrate that a Smac mimetic can induce modest reactivation of the latent SHIV reservoir, as evidenced by transient increases in plasma viremia. However, while AAV-expressed eCD4-Ig conferred partial protection against intrarectal SHIV challenge in uninfected animals, neither eCD4-Ig nor ciapavir reduced the viral reservoir in SHIV-infected rhesus macaques, as determined by total SHIV DNA and a 5-target intact provirus detection assay. Animals treated with the combination showed no significant differences in viral rebound kinetics post–analytical treatment interruption compared with controls. Additionally, repeated ciapavir dosing resulted in adverse effects in some animals, suggesting potential toxicity with repeat administration. These findings highlight the challenges in reducing viral reservoirs using this shock-and-kill approach, particularly in SHIV-infected models, and suggest that further optimization of both latency-reversing agent and immune-mediated clearance strategies is required.

## Abstract 2

A combination treatment designed to eliminate the viral reservoir in SHIV infected rhesus macaques successfully reactivated the latent virus but did not affect reservoir size.

## Introduction

HIV persists in latently infected cells because antiretroviral therapy (ART) fails to completely eradicate the virus, even as it is highly effective in reducing morbidity and mortality (1). The viral reservoir, composed of cells housing integrated, replication-competent latent proviruses, eludes detection and elimination by the immune system and current therapies. Moreover, upon cessation of ART, infectious HIV particles are produced from latently infected cells and subsequently newly infected naive cells, thereby replenishing the reservoir. The “shock and kill” approach aims to cure HIV by reactivating latent proviruses, allowing for the subsequent elimination of virus and infected cells by immune-mediated mechanisms or targeted kill treatments (2). Currently, both components of this strategy, the reactivation (shock) and elimination (kill) of infected cells, require further assessment and development before they can progress to clinical applications (3).

HIV latency-reversing agents (LRAs) with various mechanisms of action have been developed, but most preclinical studies and clinical trials have demonstrated only limited viral expression following their administration (4–13). Smac mimetics, a class of molecules that target inhibitor of apoptosis proteins (IAPs), have emerged as promising LRA candidates in recent years (14). Prior investigations into the in vivo latency-reversing potential of Smac mimetics, particularly ciapavir and AZD5582, have yielded encouraging results (15–19). These molecules, both with a bivalent structure that enables binding of 2 baculoviral IAP repeat domains of IAPs in *cis* or in *trans* configurations, were found to exhibit superior LRA activity compared with most Smac mimetics previously developed as cancer therapeutics (18, 19). Studies conducted in humanized mice, as well as in rhesus macaques infected with simian immunodeficiency virus (SIV), demonstrated the capacity of these compounds to induce replication of latent virus by activating the noncanonical NF-κB pathway without a concomitant induction of T cell activation or cytokines, representing important progress in the search for viable LRAs (15, 16, 18, 19). However, the ability of Smac mimetics to reverse latency in nonhuman primates (NHPs) infected with simian/human immunodeficiency virus (SHIV) has not been successfully demonstrated (20), indicating a gap in our understanding of their effectiveness across different models of ART-suppressed HIV-1 infection. SHIV strains are chimeric viruses based on SIV clones that incorporate certain HIV-1 genes in place of their SIV equivalents. In addition to the HIV-1 *env*, *tat*, and *rev*, SHIV constructs frequently also contain *vpu* (21–23). In the context of reservoir depletion studies, the use of chimeric SHIVs permits the investigation of monoclonal antibodies that target the HIV-1 envelope and other treatments that kill virus-expressing cells. Thus, rhesus macaques infected with SHIV and subsequently suppressed with ART could provide a relevant and controlled model to assess therapies designed to deplete the latent reservoir. In this study, we directly compared the efficacy of ciapavir and AZD5582, the 2 Smac mimetic LRA treatments with validated in vivo activity (16, 18, 19), in ART-treated rhesus macaques infected with SHIV. Additionally, we introduced a “kill” immunotherapy to complement Smac mimetic–based reactivation (“shock”) by administering adeno-associated virus (AAV) vectors expressing eCD4-Ig. This engineered, antibody-like molecule acts as a potent inhibitor of HIV-1, HIV-2, and SIV infection by recapitulating critical cellular components of the viral entry mechanism: the CD4 receptor ectodomain fused to a CCR5/CXCR4 co-receptor mimetic (24). The Ig domain engages with the immune system’s effector functions, including antibody-dependent cellular cytotoxicity (ADCC) (25). This kill treatment therefore is expected to simultaneously clear virus, prevent de novo infection of CD4^+^ T cells, and eliminate infected cells actively producing viral particles by targeting the HIV-1 Env glycoprotein expressed on the plasma membrane. While we report what we believe to be the first evidence of Smac mimetic–induced reactivation of latent SHIV, our data do not demonstrate an impact of this treatment, either alone or in combination with eCD4-Ig treatment, on the size of the viral reservoir.

## Results

### Pharmacokinetics and tolerability of ciapavir in NHPs.

**Figure 1:**
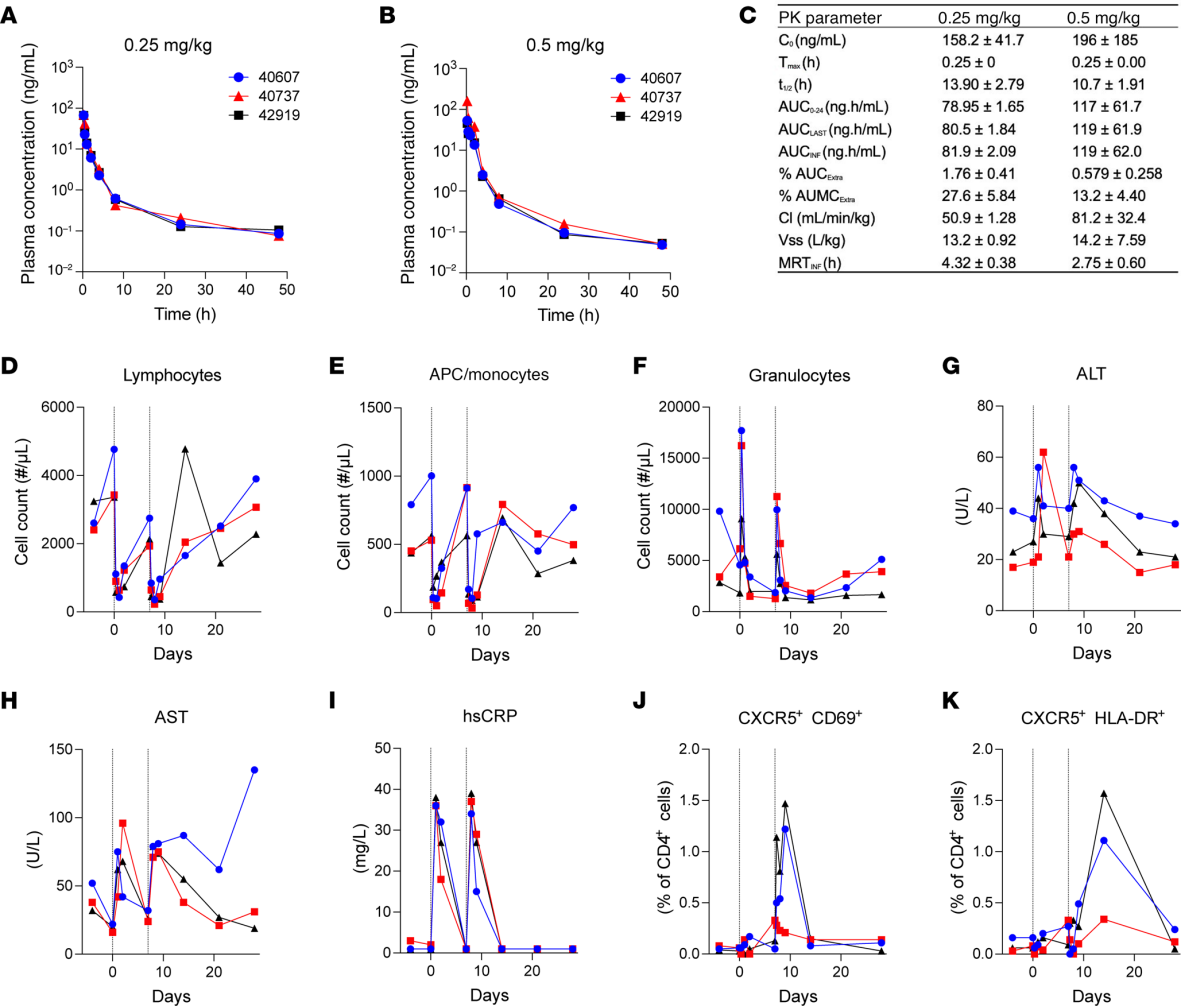
PK profiling of ciapavir in NHPs. Naive rhesus macaques were intravenously administered ciapavir at 0.25 mg/kg on day 0 (**A**) and 0.5 mg/kg on day 7 (**B**) to determine plasma concentrations of the molecule. PK parameters are shown for both doses in the table (**C**). C_0_, initial concentration; MRT_IFN_, mean residence time to infinity. Complete blood count (CBC) analysis revealed a temporary drop in lymphocyte (**D**) and monocyte (**E**) counts following drug administration as well as increases in granulocytes (**F**). Blood chemistry analysis showed modest and temporary increases in alanine aminotransferase (ALT) (**G**) and aspartate aminotransferase (AST) (**H**), as well as in high-sensitivity C-reactive protein (**I**). The second dose of ciapavir was accompanied by increases in CD69^+^ (**J**) and HLA-DR^+^ (**K**) CD4^+^ cells. Vertical lines indicate ciapavir administration on day 0 (0.25 mg/kg) and day 7 (0.5 mg/kg).

To assess the pharmacokinetics (PK) and tolerability of the Smac mimetic ciapavir in NHPs, we conducted a study in 3 uninfected Indian-origin rhesus macaques (*Macaca mulatta*). Each animal received an initial ciapavir dose of 0.25 mg/kg, administered intravenously, followed by a higher dose of 0.5 mg/kg 7 days later (Figure 1A). Blood samples were collected immediately before dosing and at 15 minutes, 30 minutes, 1 hour, 2 hours, 4 hours, 8 hours, 24 hours, and 48 hours postdosing. Peak plasma concentrations (*C_max_*) were observed at 15 minutes postdosing, with values of 158.2 ng/mL for the 0.25 mg/kg dose and 196.4 ng/mL for the 0.5 mg/kg dose (Figure 1B). Plasma half-life (*t_1/2_*) was calculated to be 13.9 hours for the lower dose and 10.7 hours for the higher dose. The area under the concentration-time curve (AUC) was 81.9 ng × h/mL for the 0.25 mg/kg dose and 119.4 ng × h/mL for the 0.5 mg/kg dose. Clearance (Cl) was observed at 50.9 mL/min/kg for the lower dose and 81.2 mL/min/kg for the higher dose, with a steady-state volume of distribution (Vss) of 13.17 L/kg and 14.2 L/kg, respectively, indicating good tissue penetration.

Tolerability assessment revealed no severe adverse effects with the administration of a single dose. Transient hematological changes, including reductions in lymphocyte and APC/monocyte counts and an increase in granulocytes, were observed postdosing but resolved within several days (Figure 1, D–F). Modest elevations in liver enzymes (alanine aminotransferase [ALT] and aspartate aminotransferase [AST]) returned to baseline levels shortly after treatment (Figure 1, G and H). High-sensitivity C-reactive protein (hsCRP) assays indicated a transient increase in this inflammatory marker 24 hours postdosing, returning to pretreatment levels by day 7 (Figure 1I). Notably, increases in CD69- and HLA-DR–positive cells were observed only after the second dose of ciapavir (Figure 1, J and K). No behavioral changes were detected in the study animals. In summary, the PK profile of ciapavir in uninfected rhesus macaques suggests effective systemic exposure and good tissue penetration. The transient changes in blood parameters and liver enzymes without severe adverse effects indicate tolerability of ciapavir but warrant careful monitoring. Since the plasma concentration of ciapavir required to effectively reverse viral latency in NHPs is not known, and both the 0.25 mg/kg and 0.5 mg/kg doses were well tolerated in the PK study, we selected the higher dose for study in SHIV-infected, ART-suppressed animals.

### AAV-expressed eCD4-Ig protects against intrarectal shiv challenge.

**Figure 2:**
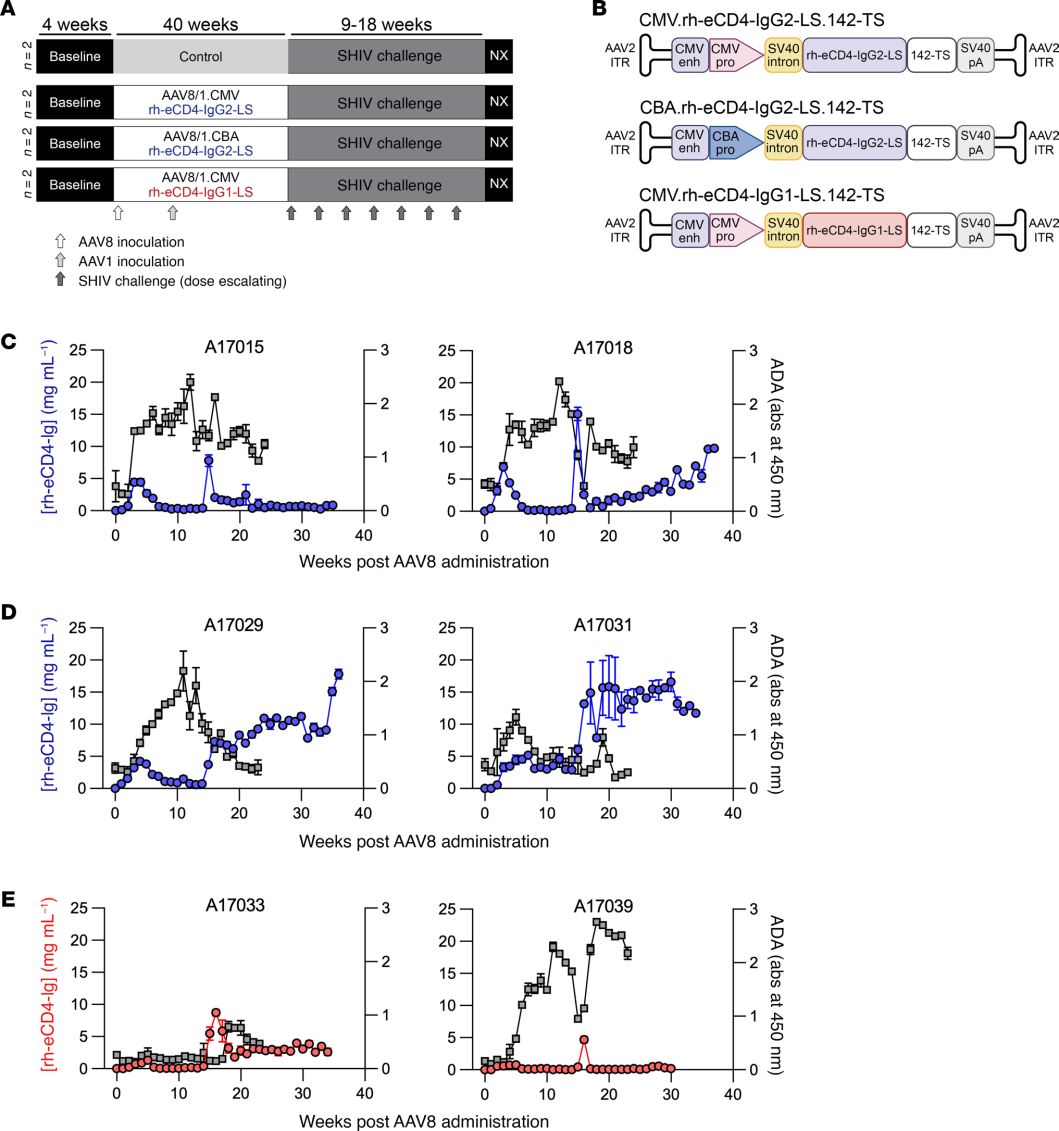
Optimized AAV-expressed rh-eCD4-Ig cassettes used in the NHP study. (**A**) Schematic of the NHP study. Four groups of 2 rhesus macaques each were used. NX, necropsy. (**B**) Designs of the AAV expression cassettes used to express rh-eCD4-Ig. Concentrations of rh-eCD4-Ig and ADA responses as measured by ELISA are shown for vectors using a CMV promoter expressing rh-eCD4-IgG2-LS (**C**), a CBA promoter expressing rh-eCD4-IgG2-LS (**D**), or a CMV promoter expressing rh-eCD4-IgG1-LS (**E**). ADA responses in panels **C**–**E** are shown as gray lines, measured by absorbance at 450 nm using a 1:10 serum dilution. Error bars in **C**–**E** represent range of duplicates per assay.

eCD4-Ig is an immunoadhesin protein that combines the properties of CD4 domains 1 and 2, which bind the viral Env protein, a sulfated coreceptor mimetic peptide, and an Ig domain. This synthetic protein represents a rationally designed broadly neutralizing agent against HIV, SIV, and SHIV (24, 26). eCD4-Ig has potential for long-term expression when administered via AAV vectors, offering a unique advantage for prophylactic as well as therapeutic applications (24, 26). Our study evaluated the expression and efficacy of 3 vectorized eCD4-Ig expression cassettes and their ability to protect against dose-escalating intrarectal (IR) SHIV challenge in 8 Indian-origin rhesus macaques (Figure 2A). Each cassette was designed to optimize eCD4-Ig expression and improve PK from our previous studies (Figure 2B). We had previously tested AAV expression cassettes that utilized the CMV promoter with a woodchuck hepatitis virus posttranscriptional regulatory element (WPRE) to express an rh-eCD4-IgG2 construct that successfully prevented SHIV-AD8 and SIVmac239 infection (24, 26). In an effort to decrease antidrug antibody (ADA) responses, we removed the WPRE and replaced it with 4 miRNA-142 target sequences in tandem to reduce expression from antigen-presenting cells (27, 28). Additionally, in the second cassette, we replaced the CMV promoter with the chicken β-actin (CBA) promoter because the CMV promoter has been associated with transcriptional silencing (29). Last, in the third cassette, we replaced rhesus IgG2 Fc with the rhesus IgG1 Fc. All cassettes included the M428L/N434S amino acid substitutions that have been described to improve FcRn binding and increase antibody half-life (30).

Fuchs et al. had previously demonstrated a prime/boost strategy that resulted in higher antibody expression with lower ADA in rhesus macaques using AAV8 and AAV1 vectors (31). In an effort to reduce ADA and increase eCD4-Ig expression from AAV vectors, we decided to replicate this strategy where 3 groups of 2 rhesus macaques each would receive 4 intramuscular (IM) injections of AAV8 rh-eCD4-Ig vectors on week 0 in the left and right quadriceps muscles followed by 4 IM injections of AAV1 rh-eCD4-Ig vectors in the left and right biceps and deltoid muscles at week 12. AAV8 vectors were administered at a 2.1 vector genomes (vg) per kilogram dose and were mixed with an AAV8 vector encoding TPST2 at a dose of 0.75 vg/kg to ensure the coreceptor mimetic peptide was sulfated. AAV1 vectors were administered at a 2.7 vg/kg dose and were mixed with an AAV1 vector encoding TPST2 at a dose of 0.75 vg/kg. The first group of macaques using the CMV promoter had peak rh-eCD4-IgG2-LS concentrations reach 7–15 μg/mL but ultimately ranged from <1 to 9 μg/mL before the challenge phase of the study (Figure 2C). ADA responses were observed in both animals starting at weeks 3 or 4 after AAV8 administration and lasted throughout the study. The second group of macaques using the CBA promoter had more sustained and constant expression of rh-eCD4-IgG2-LS ranging from 9 to 16 μg/mL for most of the study (Figure 2D). ADA was also observed in both animals but decreased between weeks 10 and 15 of the study. The third group of macaques using the CMV promoter to express rh-eCD4-IgG1-LS had the lowest concentrations and highest ADA of the 6 animals (Figure 2E). We observed peak concentrations after AAV1 administration ranging from 4 to 8 μg/mL, but by the end of the study, the concentrations decreased to <0.1 to 2.5 μg/mL. ADA was again observed in both animals but was markedly higher in A17039.

**Figure 3:**
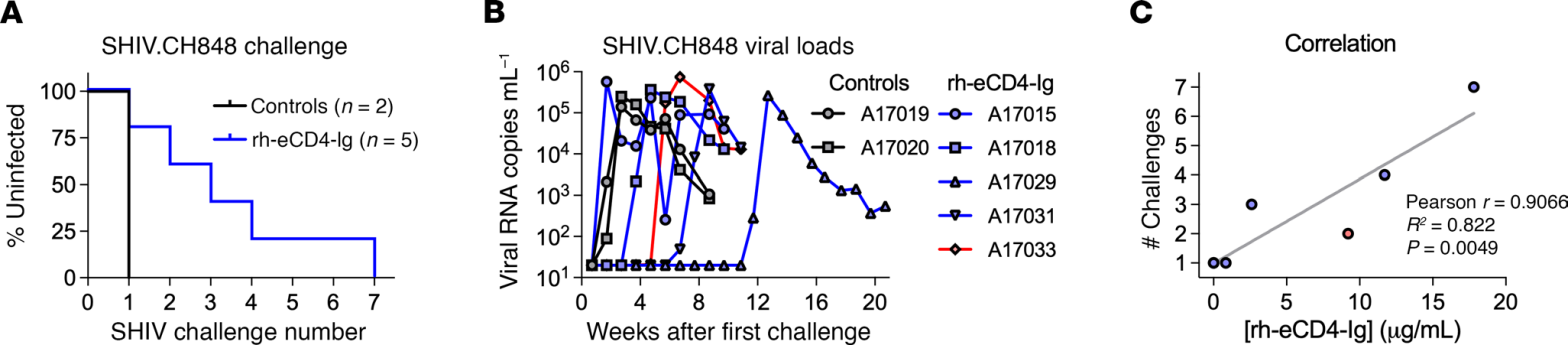
AAV-expressed rh-eCD4-Ig protects from IR SHIV-CH848 challenges. (**A**) Kaplan-Meier curve of uninfected rhesus macaques after each SHIV-CH848 challenge. Two control macaques and 5 of the rh-eCD4-Ig–expressing macaques were IR challenged 6 times with escalating doses of SHIV-CH848. Note that the seventh challenge of A17029 was a dose 10-fold higher than the sixth challenge and administered intravenously. (**B**) Viral RNA measurements of each animal as determined by qPCR. (**C**) Correlation plot of number of challenges needed for infection compared with the amount of rh-eCD4-Ig as measured by ELISA before the start of challenges. Correlation statistics are included in the graph.

We next assessed the in vivo efficacy of the expressed rh-eCD4-Ig to protect against escalating, IR SHIV-CH848 challenges. A17039 was removed from the challenge phase, as it had no detectable rh-eCD4-Ig, and was used to identify anti–rh-eCD4-Ig antibodies. Two control animals became infected after the first challenge (32), and we observed delayed acquisition in the animals expressing rh-eCD4-Ig; however, statistical significance was not assessed because of the limited control sample size (Figure 3, A and B). A17015 became infected after the first challenge, A17018 after the second, A17033 after the third, and A17031 after the fourth. Notably, A17029 was protected against 6 IR challenges and only became infected after an intravenous (IV) dose that was 10-fold higher than the last IR challenge. Moreover, we observed a strong correlation between the number of challenges before infection with the concentration of expressed rh-eCD4-Ig at the start of the challenge phase (Figure 3C). Importantly, to our knowledge, this is the first NHP study to demonstrate that rh-eCD4-Ig can protect against IR SHIV challenges.

### Characterization of NHP response to ART in study groups.

**Figure 4:**
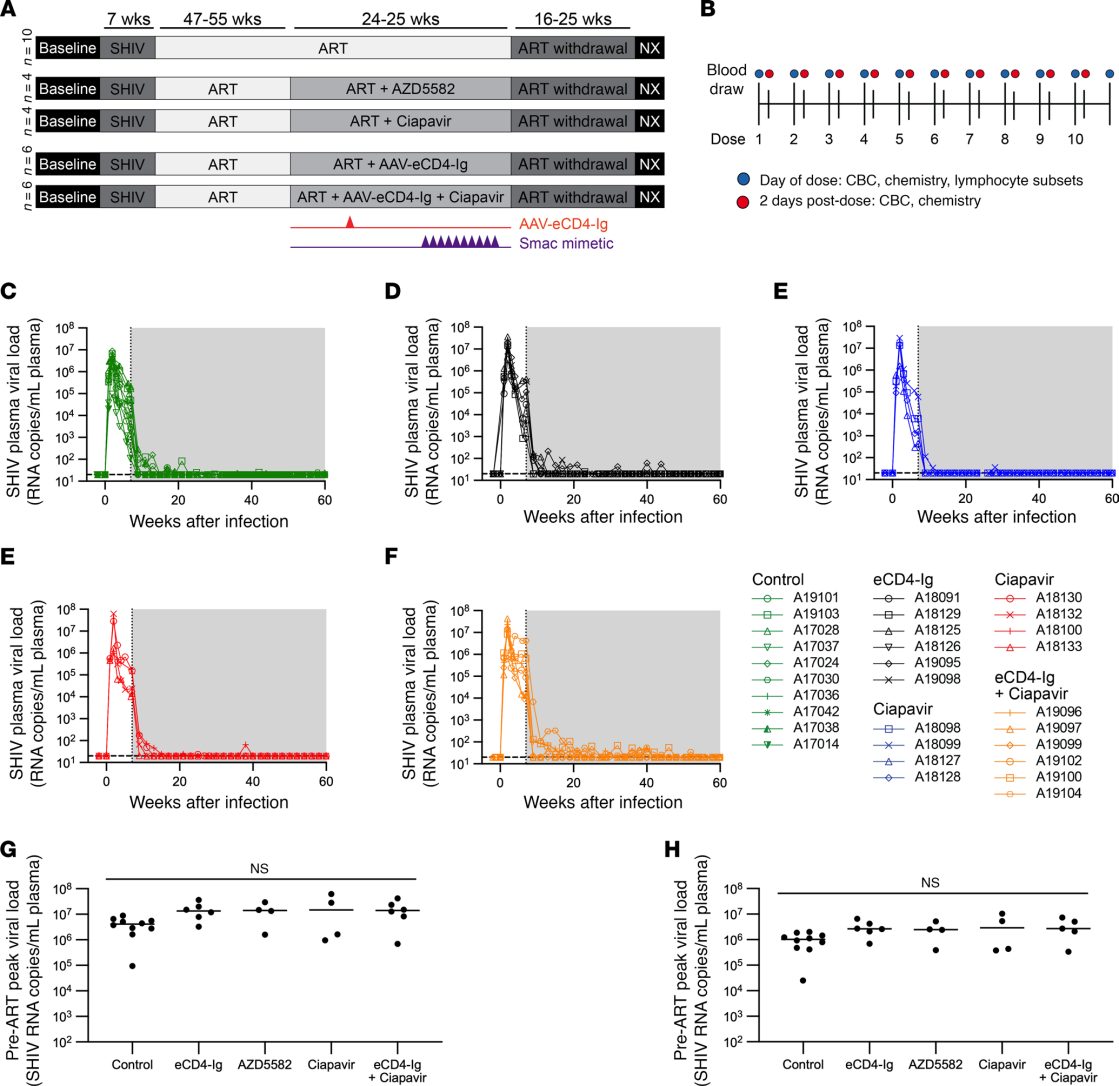
Study design and plasma viral load kinetics in ART-suppressed SHIV-infected NHPs. (**A**) Study schematic for experiments in SHIV-infected, ART-suppressed rhesus macaques. Seven weeks after infection with SHIV, animals initiated suppressive ART and were treated for at least 47 weeks before reservoir targeting with AAV-eCD4-Ig (orange arrows) and/or Smac mimetic compounds (purple arrows). ART was released after 24–25 weeks of reservoir targeting, and viral rebound was monitored for 16–25 weeks prior to end-of-study necropsy. (**B**) Smac mimetic dosing and sampling schedule for SHIV-infected, ART-suppressed rhesus macaques. Up to 10 doses of AZD5582 or ciapavir were administered, 1–2 weeks apart. Blue and red circles indicate sampling on the day of dosing and 2 days postdose, respectively. PVL is continuously monitored throughout the study in animals treated with ART only (negative control) (**C**), eCD4-Ig (**D**), AZD5582 (**E**), ciapavir (**F**), or ciapavir in combination with eCD4-Ig (**G**). Area shaded in gray represents the period of ART administration. The level of quantification (LOQ) is indicated by a black dashed line. Pre-ART peak PVL (**H**) and average PVL (**I**) indicate an equal distribution of viral loads in the animals across treatment groups. Bars indicate group median. Statistical significance was determined by pairwise comparison using the Kruskal-Wallis test with Dunn’s multiple-comparison correction.

We next tested the impact of AAV-delivered eCD4-Ig and Smac mimetics on the latent SHIV reservoir. A total of 30 rhesus macaques (*Macaca mulatta*; Supplemental Table 1; supplemental material available online with this article; https://doi.org/10.1172/JCI187961DS1) of Indian origin were infected IV with the 1157ipd3N4 strain (23, 33–38), a highly replicating and mucosally transmissible R5 virus that encodes an HIV-1 clade C envelope (Figure 4A). Blood draws were conducted biweekly throughout the study and twice per week during Smac mimetic dosing (Figure 4B). Seven weeks postinfection, animals were placed on ART consisting of daily tenofovir (TDF, 5.1 mg/kg), emtricitabine (FTC, 30 mg/kg), and dolutegravir (DTG, 2.5 mg/kg), administered subcutaneously for 72–73 weeks (39) (Figure 4A). Animals treated with ART only (*n* = 10) served as a control group (Figure 4C). Experimental groups consisting of 4 or 6 animals were used to evaluate treatment with AAV-delivered eCD4-Ig, ciapavir, or AZD5582 individually, as well as AAV-delivered eCD4-Ig in combination with ciapavir (Figure 4, D–G). Note that the treatment groups were not all studied concurrently.

With one exception in the control group, all animals displayed peak plasma viral loads (PVLs) between 6 × 10^5^ and 1 × 10^8^, and average PVLs between 3 × 10^5^ and 1 × 10^7^, prior to ART. At the time of ART initiation in week 7, PVLs ranged from 10^4^ to 10^5^ SHIV RNA copies/mL. Although difficult to discern due to ART initiation at week 7, we did observe that set point viral loads began to trend lower approximately 4 weeks postinfection in a subset of our animals, suggesting spontaneous control of SHIV. All animals were well suppressed to PVL below the LOQ (20 copies/mL) at week 60, prior to LRA treatment (Figure 4, C–G). Our study groups exhibited similar virologic parameters, without significant differences in pre-ART peak and average viral loads (Figure 4, H and I). Animals with lower average viral loads in Figure 4I are most likely to have spontaneously controlled virus replication prior to ART initiation.

### AAV9 vector delivery of rh-eCD4-IgG2-LS in SHIV-infected, ART-treated rhesus macaques.

**Figure 5:**
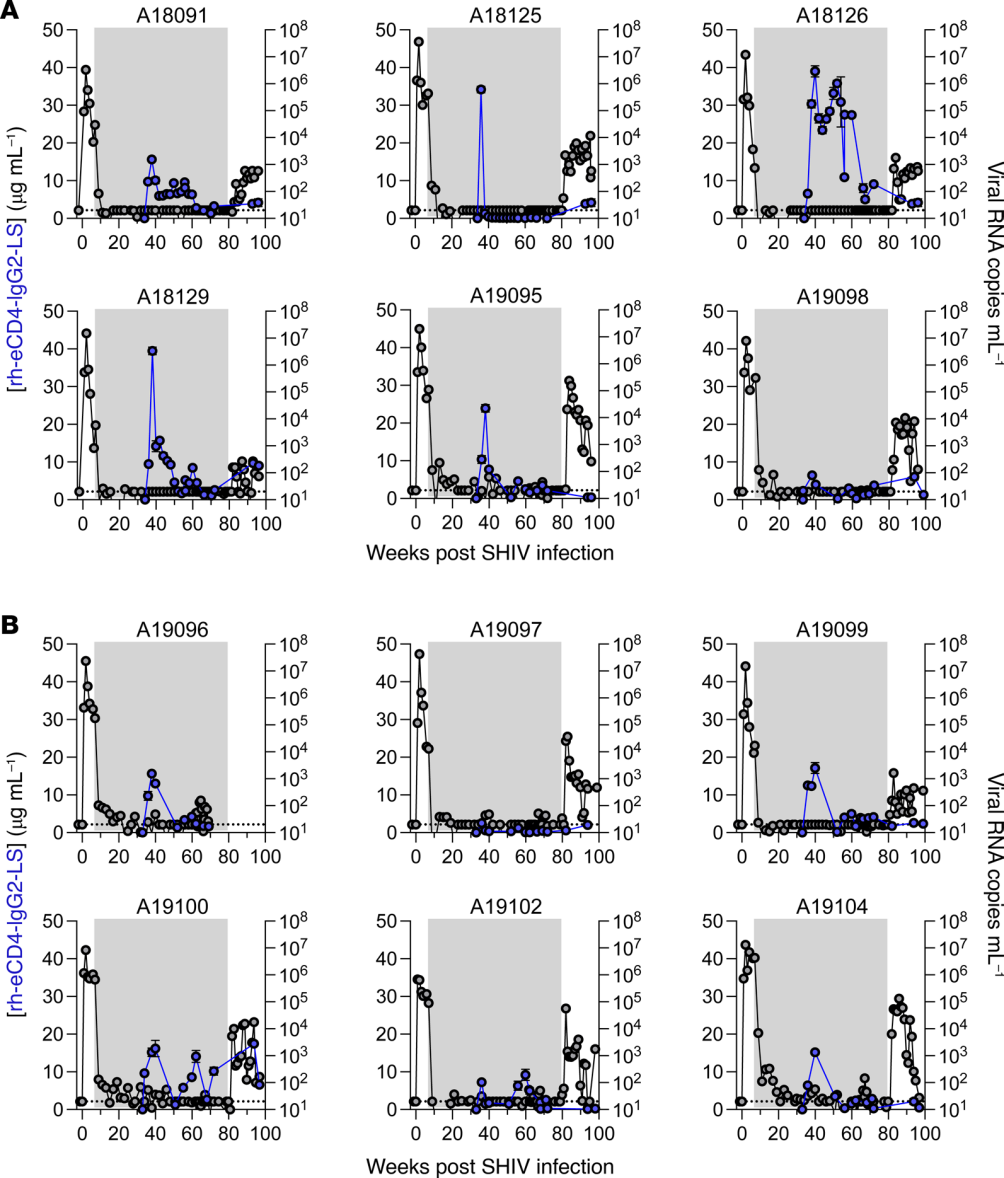
AAV9-delivered rh-eCD4-IgG2-LS concentrations in rhesus macaques with suppressed SHIV viremia mediated by ART. (**A**) Six SHIV-1157ipd3N4–infected rhesus macaques were administered AAV9 vectors encoding rh-eCD4-IgG2-LS and rhTPST2 at 34 weeks after infection while all macaques had suppressed viremia mediated by daily ART. (**B**) Same as **A** except this group of 6 rhesus macaques also received ciapavir. Rh-eCD4-IgG2-LS concentrations were measured by anti-CD4 ELISA, and concentrations are represented as the average of duplicates in blue. Error bars represent range of the duplicates. PVLs as determined by qPCR are represented in black. Area shaded in gray represents the period of ART administration.

**Figure 6:**
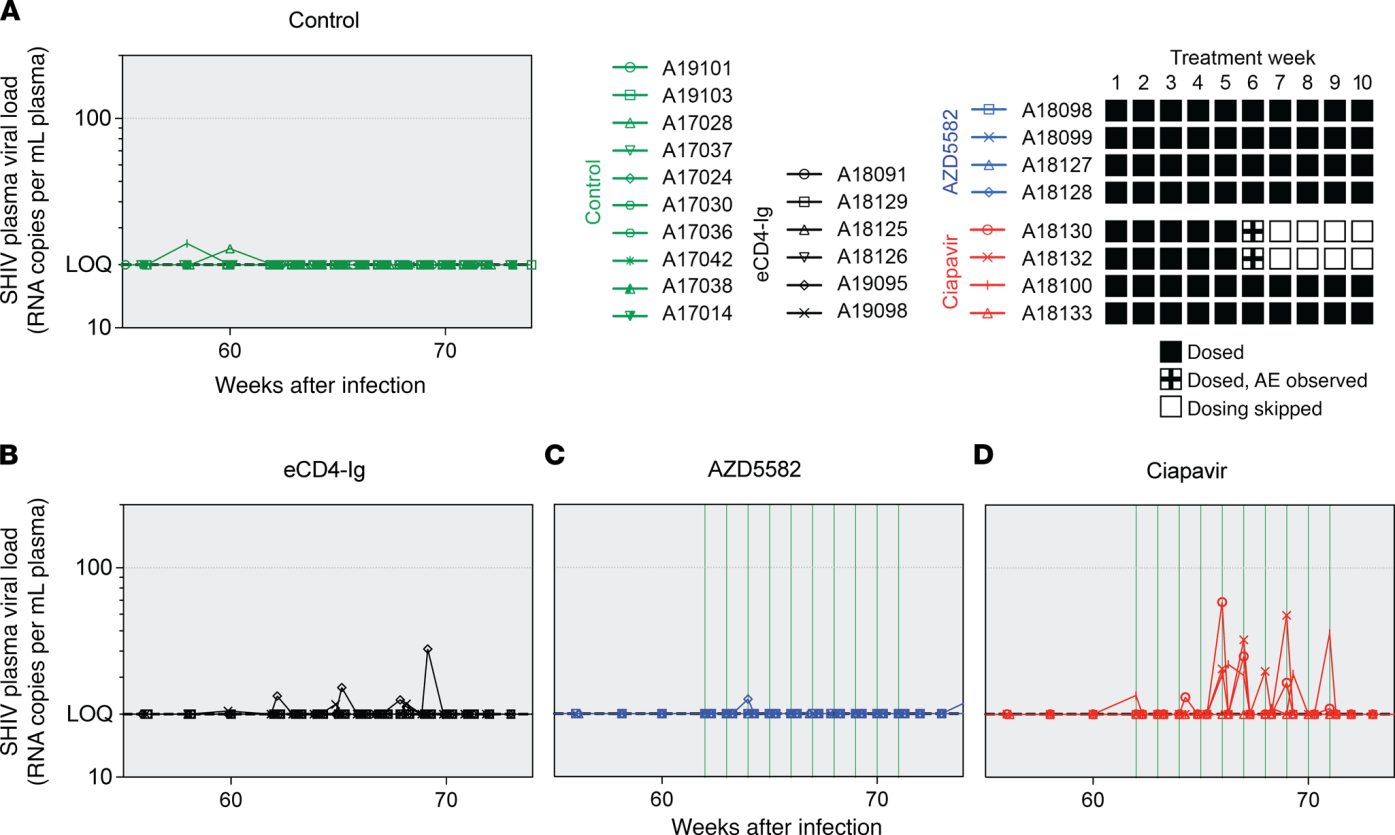
Treatment of SHIV-infected, ART-suppressed NHPs. Smac mimetics were administered to the animals in 10 weekly doses unless adverse effects were observed. Measurements of SHIV PVL in the control group (**A**) and groups treated with eCD4-Ig (**B**) or AZD5582 (**C**) did not show on-ART viremia. In the ciapavir-treated group (**D**), 3 animals showed blips of SHIV RNA in the plasma exceeding the LOQ. The LOQ is indicated by a black dashed line. Area shaded in gray represents the period of ART administration. LRA dosing is indicated by green vertical lines.

Twelve macaques were administered AAV9 vectors encoding rh-eCD4-IgG2-LS and rhTPST2 at 34 weeks postinfection (Figure 4A), including 6 without subsequent ciapavir treatment (Figure 5A) and 6 with ciapavir treatment (Figure 5B). AAV9 was selected over AAV1 and AAV8 for this study, as our previous study had demonstrated AAV9 expression in rhesus macaques to be as good as AAV1 and better than AAV8 via IM administration. We elected to use rh-eCD4-IgG2-LS expressed from the CBA promoter based on our results in Figure 2. While the rhesus macaque IgG2 Fc has reduced effector function compared with the rhesus macaque IgG1 Fc, it does have the ability to kill infected cells, unlike the human IgG2 Fc. Peak rh-eCD4-IgG2-LS concentrations ranged from 10 to 40 μg/mL in all but 3 macaques (A19097, A19098, and A19102) before dropping to concentrations <1 to 5 μg/mL in all macaques for the duration of the study (Figure 5, A and B). The lower concentrations we observed before the ATI phase did not achieve therapeutic efficacy after ART was withdrawn, as viral rebound was observed in all 12 macaques (Supplemental Figure 1).

### Ciapavir induces modest increase in PVLs in SHIV-infected rhesus macaques.

Latency reversal was defined as on-ART viremia where viral RNA in the plasma exceeded the LOQ (20 copies/mL). The Smac mimetics ciapavir or AZD5582 were administered weekly for 10 weeks, starting at week 62, or until adverse effects were observed. AZD5582 was administered at the previously established dose of 0.1 mg/kg (16, 18, 20), and ciapavir was administered at 0.5 mg/kg based on the PK evaluation. Study cohorts correspond to the schematic in Figure 4A and included an ART-only control group (Figure 6A) and animals dosed with AAV-delivered eCD4-Ig (Figure 6B), AZD5582 (Figure 6C), or ciapavir (Figure 6D). Animals treated with eCD4-Ig or AZD5582 largely resembled controls, lacking evidence of latency reversal during the treatment (Supplemental Figure 2). However, 3 of 4 ciapavir-treated animals (75%) responded with low but significant levels of on-ART viremia (Figure 6D and Supplemental Figure 2). Animals A18130, A18132, and A18100 showed multiple blips of viral RNA in the plasma following ciapavir administration.

**Figure 7:**
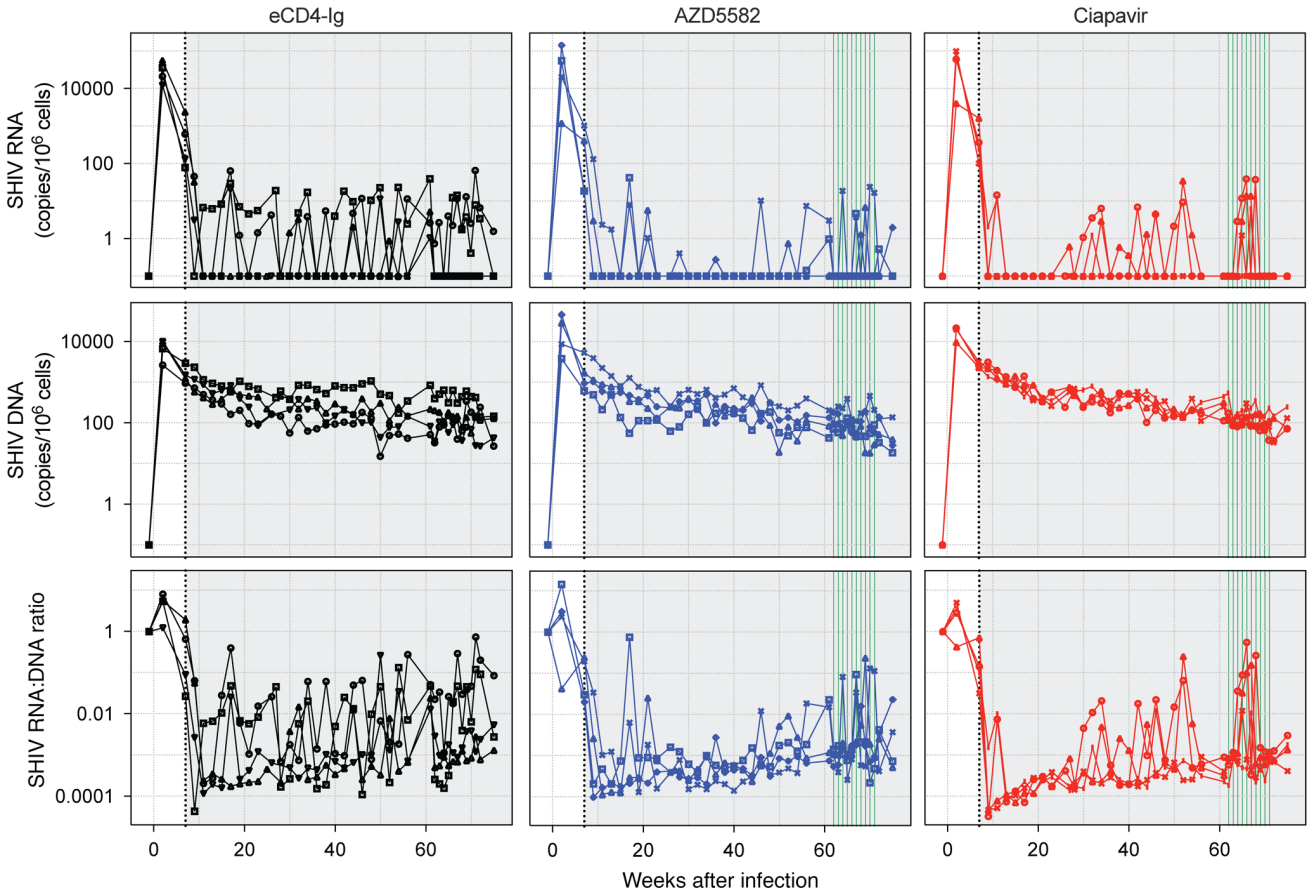
Cell-associated SHIV RNA and DNA levels in PBMCs of treated animals. Following ART initiation, cell-associated (CA) RNA levels remained constant in all treatment groups. CA DNA levels showed a constant decrease postinfection that converged toward a plateau consistent across groups. Area shaded in gray represents the period of ART administration. LRA dosing is indicated by green vertical lines.

In addition to measuring PVL, we quantified cell-associated SHIV RNA and DNA levels in PBMCs of treated animals (Figure 7). Despite PVL increases in the ciapavir-treated group, PBMC-associated SHIV RNA levels were not significantly impacted compared with levels recorded in the other groups. Levels of PBMC-associated SHIV DNA and RNA/DNA ratios remained equally unaffected. While this indicated that ciapavir treatment alone did not impact viral reservoir size, it should be noted that the signal-to-noise ratio, particularly in the RNA/DNA proportion, may preclude detection of modest effects.

### Repeat dosing of ciapavir causes adverse effects in treated animals.

Clinical assessment of repeat administration of ciapavir indicated tolerability through 5 doses without serious adverse events. However, following the sixth dose, 2 animals (A18130 and A18132) exhibited acute nausea and emesis, prompting cessation of treatment. One animal (A18132) also displayed serous nasal discharge and facial flushing. Adverse responses were managed with a regimen consisting of ondansetron, maropitant for nausea/emesis, diphenhydramine and dexamethasone for potential immune reaction, enrofloxacin for potential infection, and fluids for dehydration, resolving within 30 minutes. No such effects were noted in the remaining 2 animals treated with ciapavir or any animals treated with AZD5582 or AAV-delivered eCD4-Ig alone, indicating that the adverse reactions could be dose-/treatment-dependent or specific to certain individuals. CBC and lymphocyte subset analysis showed a temporary decrease in white blood cells and neutrophils postdosing of both Smac mimetics, ciapavir and AZD5582 (Supplemental Figure 3), similar to trends seen in the PK evaluation in uninfected animals (Figure 1, D–F). A sawtooth pattern was observed in several parameters, most notably in lymphocytes and monocytes, decreasing from the day of dosing to the 2-day postdose time point, and resolving by the next dose 7 days later (Supplemental Figures 3 and 4). Comprehensive metabolic panel parameters remained stable except for modest, temporary reductions in alkaline phosphatase (ALP) levels and increases in serum glucose levels, particularly in AZD5582-treated animals (Supplemental Figure 5).

### Combination of AAV-delivered eCD4-Ig and ciapavir does not impact the viral reservoir size in SHIV-infected rhesus macaques.

**Figure 8:**
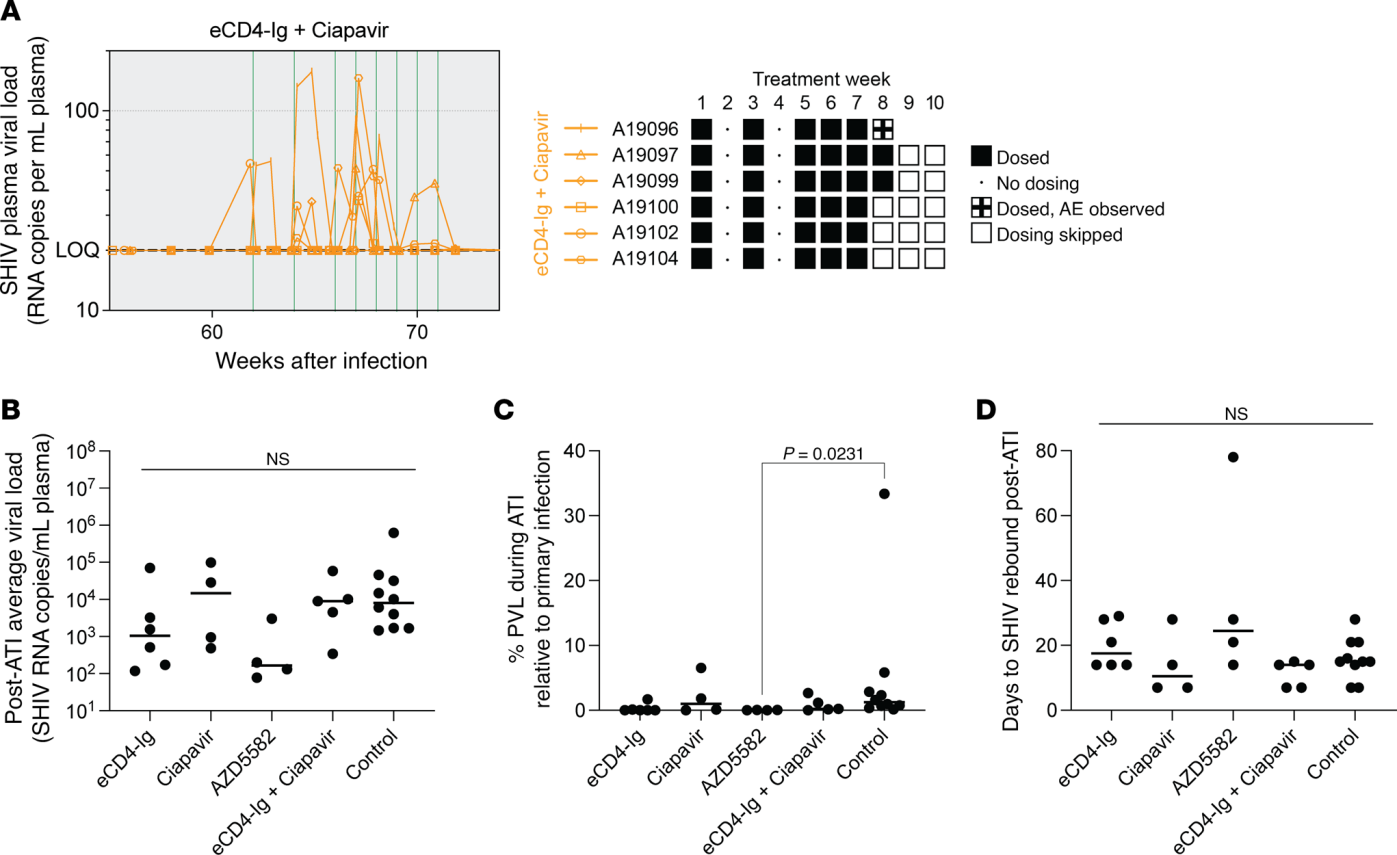
Impact of combined ciapavir and eCD4-Ig treatment on the viral reservoir. Following AAV delivery of eCD4-Ig, 6 animals were scheduled for biweekly (doses 1–3) administration of ciapavir, followed by weekly (doses 4–8) administration. After adverse effects were observed in A19096, dosing of all animals was halted. (**A**) PVL levels show repeated blips of SHIV RNA after LRA treatment. Area shaded in gray represents the period of ART administration. The LOQ is indicated by a black dashed line. (**B**) Post-ATI average viral loads did not significantly differ between treatment groups. (**C**) PVL during ATI was calculated as a percentage of average PVL during primary infection. (**D**) Days to rebound after ATI did not reveal significant differences between treatment groups. Bars indicate group median. Significance was determined with a Kruskal-Wallis test. Unless otherwise noted, pairwise comparisons of treatment groups were not significant (*P* > 0.05).

Finally, we asked whether the administration of a combined “shock” (ciapavir) and “kill” treatment (eCD4-Ig) impacts viral reservoir size following respective virus reactivation and elimination. Based on the significant induction of viremia in the ciapavir group (*P* = 0.0044, Supplemental Figure 2) compared with animals treated with AZD5582 at the administered doses, we selected ciapavir for evaluation in combination with eCD4-Ig to assess the effect of this shock-and-kill treatment on the viral reservoir. To reduce the likelihood of adverse effects upon repeated ciapavir administration, we altered the dosing schema for the treatment combination (Figure 8A). In weeks 1, 3, and 5, animals were treated with ciapavir on a biweekly schedule before returning to a weekly administration starting in week 6. Despite the adjusted dosing regimen, 1 of 6 animals in the treatment group (A19096) exhibited severe adverse effects after dose 6 (treatment week 8), characterized by lack of appetite/nausea and a spike in kidney toxicity markers that ultimately required euthanasia. Due to this severe adverse event, LRA dosing of all animals in the treatment group was halted and not resumed for the duration of the study. Notably, PVLs in this treatment group were reactivated during LRA treatment, with measurements repeatedly exceeding the LOQ of 20 copies/mL (Figure 8A).

To determine the impact of the combination treatment on the viral reservoir, an analytical treatment interruption (ATI) was conducted 8 weeks after LRA administration (79–80 weeks postinfection, 72–73 weeks after ART initiation) in all study groups, including single and combination treatments (Supplemental Figure 1). Analysis of average viral loads prior to initiation of suppressive ART and reservoir-targeting treatments confirmed equal distribution between groups (Figure 4, H and I). Following LRA treatment, a post-ATI average viral load was determined for each animal by averaging measurements over the first 7 weeks of recrudescent viremia, providing a comparable window to pre-ART average viral loads (37, 40) (Figure 8B). Comparisons between pre- and post-ART PVLs did not reveal significant differences between treatment groups. Likewise, calculating post-ATI PVL as percentage of pre-ART PVL to assess rebound did not indicate a meaningful impact of treatments on the viral reservoir (Figure 8C). Time-to-rebound, calculated as days to SHIV rebound post-ATI and serving as an additional measure to detect changes in reservoir size, did not show significant differences between the study arms (Figure 8D). The data suggest that neither the individual treatments with Smac mimetics, nor AAV-delivered eCD4-Ig, nor the combination of ciapavir and eCD4-Ig mediated a measurable impact on the magnitude or kinetics of the viral reservoir.

**Figure 9:**
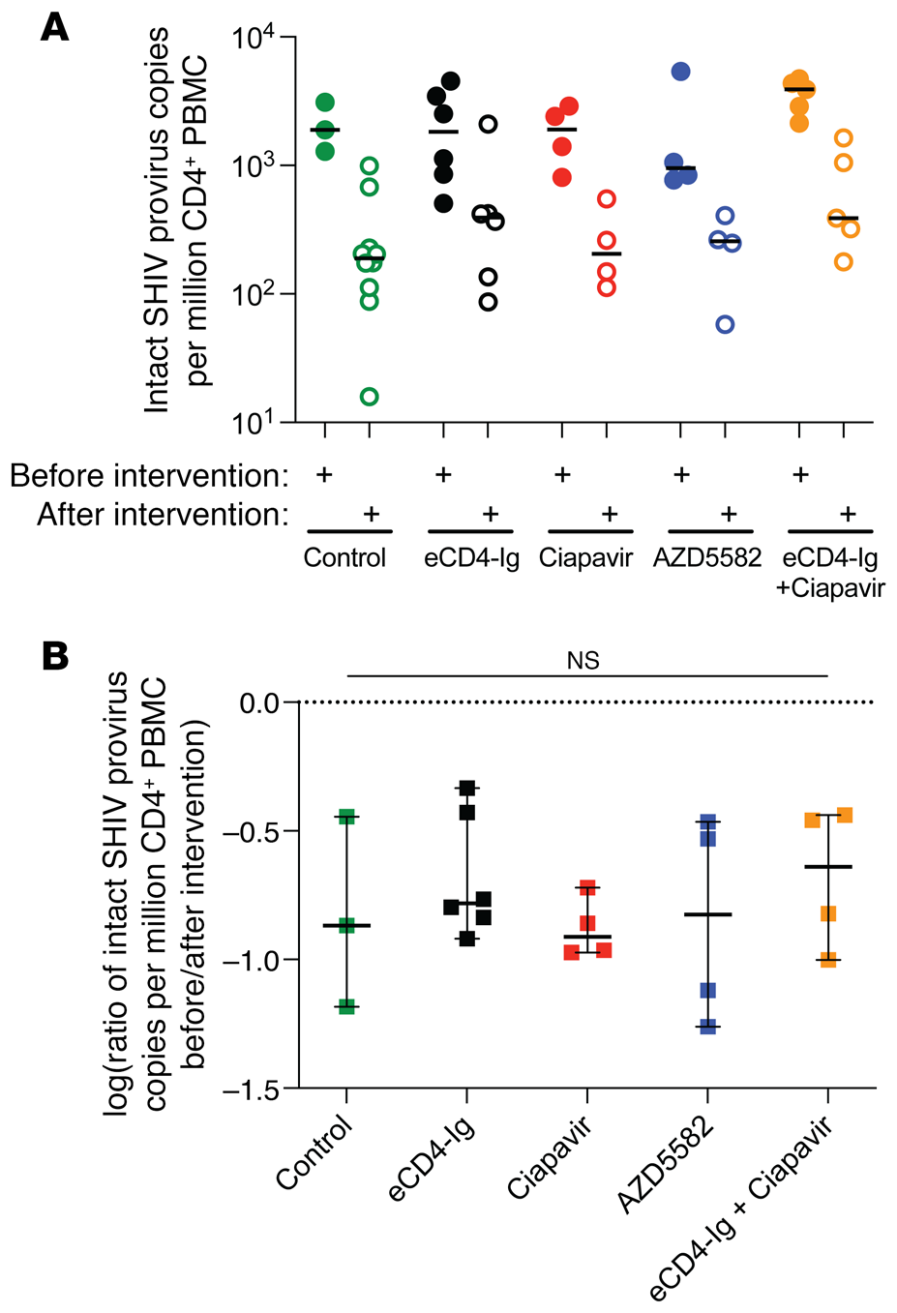
A SHIV-specific 5T-IPDA shows no impact of eCD4-Ig or LRAs in vivo. (**A**) Intact proviral SHIV DNA copies per million CD4^+^ T cells was measured by SHIV 5T-IPDA before (filled circles) and after (unfilled circles) treatment. Horizontal bars represent group median. (**B**) Log_10_ fold-change in intact proviral SHIV DNA copies per million CD4^+^ T cells calculated for each rhesus macaque with matched pre- and posttreatment 5T-IPDA measurements. Each square represents an individual animal. Missing data points indicate animals that lacked sufficient pre- or posttreatment samples for analysis. Horizontal bars denote group medians, and vertical T bars represent 95% confidence intervals for the median. No statistically significant differences in fold-change between groups were detected by Kruskal-Wallis test (*P* = 0.564).

Although our measurements of total SHIV DNA (Figure 7) and PVL kinetics post-ATI (Figure 8, B–D) failed to detect an impact of our shock-and-kill approach, these assays are often insufficient to detect partial effects. To more quantitatively assess the how eCD4-Ig and Smac mimetics may have altered SHIV reservoir size, we conducted 2 additional experiments. First, we optimized a SHIV-specific 5-target intact provirus detection assay (5T-IPDA), applying our expertise from similar assays for people living with HIV (41). Using leukapheresis products collected before and after reservoir targeting, we were able to quantify reservoir size in a total of 51 samples in our *n* = 30 study. In SHIV-infected, ART-suppressed controls that did not receive reservoir-targeting therapies, we observed a natural decay in reservoir size (Figure 9A) consistent with past reports in the NHP/SHIV model (42). Comparing log_10_ fold-changes in reservoir size between each group reinforces the notion that neither eCD4-Ig nor Smac mimetics increased or decreased viral reservoir size relative to controls (Figure 9B). Finally, we asked whether modest selective pressures mediated by our treatments affected viral diversity. Since our SHIV was not barcoded, we generated and compared full-length Env sequences as a surrogate measure of eCD4-Ig and/or Smac mimetics’ impact on virus replication following ART interruption. Plasma samples from 8 rhesus macaques representing the eCD4-Ig, eCD4-Ig + ciapavir, and untreated control groups were analyzed by deep sequencing of the full-length SHIV-1157ipd3N4 *env* gene after viral rebound (Supplemental Table 2). Supplemental Figure 6 shows very low amino acid change rates across the entire Env protein, including areas previously identified for resistance mutations to rh-eCD4-Ig (26, 43). Amino acid changes observed in the regions of interest were minimal and not at residues that have been shown to mediate resistance to rh-eCD4-Ig neutralization, ruling out the emergence of treatment-resistant variants as contributing to the inability to achieve significant reservoir reduction. The lack of measurable Env diversity that we observed reinforces our conclusion that our eCD4-Ig and LRA treatments failed to measurably impact recrudescent SHIV replication following ATI.

## Discussion

We used SHIV 1157ipd3N4-infected rhesus macaques as a model to investigate an eCD4-Ig–based clearance treatment, combined with a Smac mimetic as an LRA. Because they incorporate HIV-1 Env, SHIVs represent a valuable tool to evaluate clearance strategies designed to target the viral reservoir in NHP models without requiring modifications to target SIV-specific Env. Numerous studies have demonstrated the utility of SHIV constructs in NHP latency models (44). However, previous efforts to reverse latency in SHIV-infected rhesus macaques using a Smac mimetic, namely AZD5582, have been unsuccessful (20). Thus, it has remained unclear if latency reversal can be induced by Smac mimetics in a SHIV model, or if the virus is refractory to this class of LRAs in vivo. Differences in the viral LTR and NF-κB binding sites could affect the responsiveness to certain LRAs. The study by Dashti et al. targeted SHIV latency with AZD5582 and reported spontaneous decrease of plasma SHIV RNA levels and low PVL in some animals prior to ART initiation (20). Moreover, levels of pre-ART viremia and CD4^+^ T cell–associated DNA were lower in SHIV- than SIV-infected animals in a comparable study (18). These observations suggest that i) SHIV forms a smaller viral reservoir compared with SIV or ii) that the reservoir is kept latent by specific immune and cellular mechanisms, thus rendering latency reversal more difficult.

Our data indicate that the failure of AZD5582 treatment to reverse SHIV latency in NHPs in vivo — also reported by Dashti et al. (20) — is not due to a fundamental inability of Smac mimetics to reactivate this virus and that the LRA activity of this class of molecules extends to HIV, SIV, and SHIV. Despite relatively modest PVL increases, we observed significant induction of viremia in SHIV-infected animals upon ciapavir treatment (Supplemental Figure 2), demonstrating that SHIV can be reactivated by this Smac mimetic. However, consistent with previous reports (20), we did not observe an impact of AZD5582 treatment on SHIV latency reversal. Since the 2 compounds in our study were administered at different doses — determined by previous publications and our own PK assessment — we cannot attribute differences in LRA potential to structural differences between the compounds. Further analyses that compare LRA activity and tolerability of the molecules at dose equivalency, based on bioavailability of each compound, would be required to draw conclusions about their relative efficacy. Furthermore, the development of robust pharmacodynamic markers of Smac mimetic–mediated pathway activation would also provide critical insight into the comparative in vivo efficacy of this class of molecules.

Though we did not demonstrate natural control of SHIV in our study, we found cell-associated viral DNA levels to be lower than those reported in SIV-infected rhesus macaques where latency reversal was achieved by AZD5582 (16, 18). Based on these data, we concur with Dashti et al. that a smaller SHIV reservoir, and consequently a lower potential for latency reversal, is a likely cause for the absence of AZD5582 LRA activity (16). Thus, while SHIV allows the evaluation of clearance strategies that target the HIV-1 Env, this model in its current form may not be a sufficiently sensitive platform to study shock-and-kill treatments that require the reactivation of latent virus prior to elimination of virus-expressing cells. Further investigations into the reactivation potential of the latent reservoir in SHIV models compared with SIV and HIV-1 may be necessary to better understand the differences between these models.

Measurements of PVL rebound magnitude and time-to-rebound post-ATI did not detect changes in the size of the latent viral reservoir in animals treated with eCD4-Ig and ciapavir versus controls. It remains unclear if this failure is the result of suboptimal latency reversal by ciapavir or inefficient reservoir clearance by eCD4-Ig. While measurable and statistically significant, latency reversal in our study remained low compared with administration of a Smac mimetic to SIV- rather than SHIV-infected NHPs (16, 18). If only a small number of cells express viral proteins after reactivation treatment, the targets for eCD4-Ig would be limited and its potential effect not detectable. Beyond suboptimal efficacy of the LRA treatment, the low level and duration of eCD4-Ig expression, as well as the presence of anti–eCD4-Ig antibodies, suggest that the clearance treatment may have failed to provide sufficient eCD4-Ig levels at the time of latency reversal to induce effective ADCC-mediated elimination of infected cells. Most likely, both components of the shock-and-kill treatment will require improvement to effectively deplete the viral reservoir, which may involve combinations of LRAs as well as combinations of kill strategies. We validated our PVL-based metrics of reservoir size with 2 more quantitative assays: full-length sequencing of the clade C HIV *env* gene in our SHIV-infected animals and a pioneering SHIV-adapted 5T-IPDA. *Env* diversity was limited by our use of a clonal inoculum of SHIV-1157ipd3N4, which was not molecularly barcoded (23), and by initiation of ART 7 weeks postinfection, which may be suboptimal (45). Nevertheless, we found no evidence that either our eCD4-Ig or Smac mimetic therapies exerted selective pressure following ART interruption. Although 2-target SHIV IPDAs have been reported (42, 46), to our knowledge we present the first 5T-IPDA designed specifically for the NHP/SHIV model. Our 5T-IPDA is likely to provide a more accurate assessment of viral reservoir size (47). While our 5T-IPDA results were fully consistent with other data showing no effect of our shock-and-kill strategy on the latent reservoir, we have now developed 5-target assays for both SHIV and HIV (41). The ability to apply orthogonally designed IPDAs both for HIV and SHIV represents an important advance for assessing reservoir dynamics and benchmarking NHP models against clinical studies in people living with HIV.

Safety is a key concern for the clinical development of any LRA. While several classes of compounds have shown efficacy in vitro, their safety profiles have hindered transition to the clinic. PKC agonists, for example, effectively reverse HIV-1 latency in vitro and ex vivo, but adverse effects observed in clinical studies of bryostatin likely preclude their use in therapeutic regimens (48). Smac mimetics have so far been the most promising class of LRAs to reverse viral latency in NHP. While individual doses of AZD5582 and ciapavir have been reported to be well tolerated, our data and observations from a previous study of AZD5582 in SIV-infected macaques suggest that repeat administration of these drugs may require additional caution (18, 19). We observed similar effects on lymphocyte populations in the clinical data for AZD5582 and ciapavir, 2 distinct chemotypes with similar mechanisms of action. This suggests that these effects could be related to the mechanism of action of Smac mimetics rather than being a result of off-target effects. Notably, Nixon et al. reported adverse reactions in 1 macaque following the seventh and eighth doses of AZD5582 that mirror the effects observed in our study in 2 animals treated with ciapavir as a single agent. The symptoms included fever, emesis, fatigue, and lack of appetite (18). In both studies, these effects occurred after 7 and 6 weekly doses of the Smac mimetic, respectively. With a half-life of approximately 10 hours, repeated weekly administration of ciapavir is not expected to impact peak concentrations of the drug in the bloodstream (*C_max_*) after each dosing. However, the slow kinetics of the noncanonical NF-κB pathway activation and reset following Smac mimetic administration, with pathway activity continuing beyond the presence of the drug (14, 18), suggests that adverse events may not directly track to plasma concentrations of the drug. Alternatively, the molecules may depot in unknown cellular or organ compartments. Either of these scenarios could lead to cumulative increases in pathway activity over a repeat-dosing regimen, resulting in toxicity. As noted previously, no comprehensive assessment of the pharmacodynamics of Smac mimetics in NHPs in the context of viral latency has been published. Thus, it remains unclear if the weekly dosing schedule utilized by our study and in previous publications (16, 18–20) is optimal for safety and efficacy or if it could lead to cumulative toxicity, resulting in the adverse effects observed in some of the treated animals. Addressing these cumulative toxicity concerns will likely require pharmacodynamic biomarkers to carefully develop next-generation small molecules. Such biomarkers could play key roles in monitoring pathway activation, designing optimized dosing regimens for pathway reset and recovery, pursuing molecular optimization to improve pharmacodynamic properties, and employing combinatorial approaches with complementary LRAs. The central goal of these future efforts should include achieving synergistic latency reversal, reducing individual drug exposure, and maximizing effector-mediated clearance of latently infected cells.

In summary, our study provides in vivo evidence that SHIV reservoirs are amenable to reactivation mediated by a Smac mimetic and clarifies model-specific constraints that blunt measurable clearance. We also introduce a SHIV-adapted 5T-IPDA to benchmark reservoir dynamics and to align SHIV studies with SIV and clinical settings. The pattern of adverse events with repeat dosing suggests a mechanism involving cumulative pathway activation, which supports pharmacodynamic-informed dosing schedules and medicinal-chemistry optimization. Collectively, these findings indicate that meaningful reservoir depletion will likely require stronger and more durable clearance modalities, potentially in rational combinations, tested alongside dose-equivalent LRA comparisons, to facilitate translation to the clinic.

## Methods

### Sex as a biological variable

Sex was considered as a biological variable in our studies. However, due to limited availability of female macaques from domestic breeding colonies, we were unable to achieve a balanced cohort of males and females. All animals in the PK and AAV optimization experiments (Figures 1 and 2) were male. Of the 30 animals in the ART-suppressed SHIV experiment, 25 were male and 5 were female (Supplemental Table 1).

### PK evaluation of ciapavir

#### Animal care and study design.

All animals had visual and auditory access to other macaques 24 hours per day and were fed a balanced commercial macaque chow (Purina Mills) twice daily with fresh produce twice weekly and free access to water 24 hours per day. All procedures were carried out under ketamine anesthesia by trained personnel under the supervision of veterinary staff. Three male rhesus macaques aged 8–10 years were selected for the PK and tolerability study of ciapavir. Each animal received an initial dose of 0.25 mg/kg IV and a higher dose of 0.5 mg/kg 7 days later, both administered in Plasma-Lyte A (Baxter) at a concentration of 0.5 mg/mL. The rectal temperature and body weight of the animals were recorded before and after (days 1, 2, and 7) each administration. Different anticoagulants were used in this study: sodium citrate for coagulation tests and EDTA for CBCs as well as immunophenotyping of cells by flow cytometry. Blood collected with no anticoagulants was used for chemistry examinations.

#### Analysis of chemistry parameters, CBC, and liver coagulation function.

Blood chemistry parameters of sera samples were measured using an AU480 Chemistry Analyzer (Beckman Coulter). Analysis included sodium (mM/L), potassium (mM/L), chloride (mM/L), carbon dioxide total amount (mM/L), anion gap (mM/L), inorganic phosphorous (mg/dL), calcium (mg/dL), blood urea nitrogen (mg/dL), creatinine (mg/dL), glucose (mg/dL), total protein (g/dL), albumin (g/dL), alanine aminotransferase (ALT; U/L), aspartate aminotransferase (AST; U/L), creatine phosphokinase (CPK; U/L), alkaline phosphatase (ALP; U/L), γ-glutamyl transferase (GGT; U/L), lactate dehydrogenase (LDH; U/L), cholesterol (mg/dL), triglyceride (mg/dL), total bilirubin (mg/dL), direct bilirubin (mg/dL), and high-sensitivity C-reactive protein (hsCRP; mg/L). CBCs were performed using the ABX Pentra 60 C+ Hematology Analyzer (HORIBA Medical). Analysis included white blood cell count (K/μL), red blood cell count (M/μL), hemoglobin (g/dL), hematocrit (%), mean corpuscular volume (fL), mean corpuscular hemoglobin (pg), mean corpuscular hemoglobin concentration (g/dL), and platelets (K/μL). Differential white blood cell counts were conducted by immunophenotyping of cells by flow cytometry. EDTA-treated venous blood samples were stained with fluorochrome-conjugated monoclonal antibodies against human cell markers (rhesus macaque cross-reactive) including anti-CD3-BUV737 (clone SP34-2, BD Biosciences), anti-CD4-BUV395 (clone L200, Thermo Fisher Scientific), anti-CD8-BUV805 (clone SK1, BD Biosciences), anti-CD11b-BV785 (clone ICRF44, BioLegend), anti-CD11c-AF700 (clone 3.9, BD Biosciences), anti-CD14-BV510 (clone M5E2, BioLegend), anti-CD16-BV605 (clone 3G8, BD Biosciences), anti-CD20-PE-Cy5 (clone 2H7, Thermo Fisher Scientific), anti-CD28-APC-Cy7 (clone CD28.2, Thermo Fisher Scientific), anti-CD95-APC (clone DX2, BioLegend), anti-CD159a-VioBright FITC (clone REA110, Miltenyi Biotec), and anti-HLA-DR-PerCP-Cy5.5 (clone L243, Miltenyi Biotec). Lysis and fixation of whole-blood samples were performed using the TQ-Prep Workstation and IMMUNOPREP reagent system (Beckman Coulter). Cells were acquired on a BD Biosciences FACSymphony flow cytometer operated with FACSDiva software (BD Biosciences). Data were analyzed using FlowJo software (BD Biosciences). Coagulation function tests, including prothrombin time, activated partial thromboplastin time, and quantitative fibrinogen (mg/dL), were measured by Quality Vet Lab LLC. PK analysis of the compound was conducted by quantifying plasma concentrations using a SCIEX 6500 mass spectrometer coupled to an HPLC system equipped with a Phenomenex Luna C18(2) column (50 × 2 mm, 5 μm, 100 Å). PK parameters, including AUC, initial concentration (*C_0_*), and half-life (*t_1/2_*), were calculated using Phoenix WinNonlin 8.1 through noncompartmental analysis.

### AAV vector production and administration

AAV1, AAV8, and AAV9 vectors were produced by the University of Massachusetts and University of Pennsylvania Vector Cores by triple cesium chloride purification as previously described (49). All animals were prescreened for AAV1, AAV8, or AAV9 neutralizing antibodies prior to the start of the study as previously described (50–52). For animals A17015, A17018, A17029, A17031, A17033, and A17039, AAV8 vectors encoding rh-eCD4-IgG2-LS or rh-eCD4-IgG1-LS were diluted in sterile PBS to a dose of 2.1 × 10^12^ vg/kg and mixed with AAV8 vectors encoding rhTPST2 at a dose of 7.5 × 10^11^ vg/kg and administered IM in the upper and lower portions of the left and right quadriceps muscles while under sedation. At 14 weeks after AAV8 administration, AAV1 vectors encoding rh-eCD4-IgG2-LS or rh-eCD4-IgG1-LS were diluted in sterile PBS to a dose of 2.7 × 10^12^ vg/kg and mixed with AAV1 vectors encoding rhTPST2 at a dose of 7.5 × 10^11^ vg/kg and administered IM to the left and right biceps and deltoid muscles while under sedation. Animals A18091, A18125, A1826, A18129, and A19095 – A19104 received AAV9 vectors encoding rh-eCD4-IgG2-LS diluted in sterile PBS to a dose of 2.4 × 10^12^ vg/kg and mixed with AAV9 vectors encoding rhTPST2 at a dose of 7.5 × 10^11^ vg/kg delivered IM to the upper and lower portions of the left and right quadriceps muscles, the left and right biceps muscles, and the left and right deltoid muscles while under sedation.

### SHIV infection and virology assays

Rhesus macaques (*Macaca mulatta*) were housed and cared for under conditions that meet NIH standards as stated in the *Guide for the Care and Use of Laboratory Animals* (National Research Council, National Academies Press, Washington, DC, 1996), Institute for Laboratory Animal Research (ILAR) recommendations, and Association for Assessment and Accreditation of Laboratory Animal Care International (AAALAC) accreditation standards, as described previously (53). All rhesus macaques in this study were sourced from domestic national primate research centers (NPRCs), including Oregon, California, and Tulane NPRCs. IV SHIV infection with SHIV-1157ipd3N4 and dose-escalating IR challenge with SHIV.C.CH848 were conducted essentially as previously described with SHIV.C.CH848 (73 ng/mL SIV p27 Gag by ELISA and 4.42 × 10^6^ infectious units/mL by TZM-bl assay). A series of up to 6 IR challenges were performed every other week. The first 2 challenges used a 1:80 diluted stock, followed by 4 challenges with a 1:40 stock. On the day of each challenge, diluted stocks were freshly prepared in a total volume of 1 mL RPMI 1640 medium without additives (32). Animals that were not infected following the sixth IR challenge were subsequently infected IV with 10 ng p27 of SHIV.C.CH848. In ART-suppressed infection experiments, an IV dose of 0.5 mL SHIV-1157ipd3N4 (8,350 tissue culture ID_50_ [TCID_50_], 1.67 × 10^4^/mL TCID_50_ in TZM-bl cells) was followed 7 weeks later by initiation of suppressive therapy (54). ART consisted of the tenofovir prodrug TDF, FTC, and DTG (39, 55), administered as a daily subcutaneous injection of 1 mL/kg body weight. PVL assays amplified a region of SIV Gag specific to unspliced viral RNA found in both SHIV strains. TaqMan primer probe sequences were 5′-GCAGAGGAGGAAATTACCCAGTAC-3′ (forward), 5′-CAATTTTACCCAGGCATTTAATGTT-3′ (reverse), and 5′-TGTCCACCTGCCATTAAGCCCGA-3′ (probe). PBMC-associated SHIV DNA and RNA were quantified as described previously (33, 37, 40). SHIV PVL measurements, CBCs, serum chemistries, and lymphocyte subset analyses were performed as previously described (32, 36, 56). Blood chemistries were analyzed by the University of Washington Clinical Laboratory.

### LRA administration

AZD5582 was dosed as previously described (18). Briefly, the 0.1 mg/kg dose consisted of a 0.4 mg/mL solution prepared in 10% sterile captisol in distilled water and administered at 0.25 mL/kg body weight via the IV route. Ciapavir was also dosed IV at 0.5 mg/kg: A 0.5 mg/mL solution was prepared in PlasmaLyte A and administered IV at 1 mL/kg body weight.

### rh-eCD4-Ig ELISA

Concentrations of rh-eCD4-Ig (IgG2-LS or IgG1-LS) were determined by an anti-CD4 ELISA. A monoclonal mouse anti-human CD4 antibody (Sigma SAB4700059) was coated onto half-well ELISA plates at 3 μg/mL and incubated at 4°C overnight. Plates were washed twice with PBS-T (1× PBS with 0.05% Tween-20) and blocked for 1 hour at 37°C with blocking buffer (2% BSA in PBS). A standard of rh-eCD4-Ig was generated by diluting purified protein to a concentration of 0.5 μg/mL in blocking buffer and diluted 2-fold 14 times. Plasma samples were diluted 1:20 in blocking buffer and further diluted 2-fold 7 times. The standard and diluted samples were plated on the blocked ELISA plate and incubated for 1 hour at 37°C. Plates were washed 5 times with PBS-T, and an HRP-conjugated anti-human Fc secondary antibody (Jackson ImmunoResearch 109-036-008) was added. Plates were incubated for 1 hour at 37°C and washed 10 times with PBS-T. TMB Start Solution (Thermo Fisher Scientific) was added to the wells and developed for 5–10 minutes before being stopped by an acidic TMB stop solution (KPL). Plates were read on a BioTek Synergy Neo2 plate reader at an absorbance of 450 nm. Concentrations were determined with GraphPad Prism using samples that fit on the linear portion of the standard curve.

### ADA ELISA

ADA ELISAs were conducted similarly to the rh-eCD4-Ig ELISA. Purified rh-eCD4-Igs (IgG2-LS or IgG1-LS) were used to coat half-well ELISA plates a 3 μg/mL and incubated overnight at 4°C. Plates were washed twice with PBS-T and blocked for 1 hour at 37°C with blocking buffer (2% BSA in PBS). Plasma samples were diluted 1:10 in blocking buffer. The diluted samples were plated on the blocked ELISA plate and incubated for 1 hour at 37°C. Plates were washed 5 times with PBS-T, and HRP-conjugated anti-human κ and λ light chain secondary antibodies (Millipore AP502P and AP506P, respectively) were added. Plates were incubated for 1 hour at 37°C and washed 10 times with PBS-T. TMB Start Solution was added to the wells and developed for 5–10 minutes before being stopped by an acidic TMB stop solution. Plates were read on a Synergy Neo2 plate reader at an absorbance of 450 nm.

### SHIV 5T-IPDA

To quantify intact proviral reservoirs, a SHIV-specific 5T-IPDA was developed based on our previously published HIV IPDA (41). Briefly, CD4^+^ T cells were isolated from leukapheresis samples collected before (18–22 weeks post-ART) and after (69–73 weeks post-ART) reservoir-targeting treatments. Genomic DNA was extracted using methods optimized to minimize shearing, then analyzed by droplet digital PCR using a multiplexed assay targeting 5 regions within the SHIV genome (gag, LTR, env, pol, and tat). Quintuple-positive droplets were scored as intact proviruses. To correct for DNA shearing during extraction, a reference assay targeting the macaque RNase P p30 gene (MRPP30) with primers spaced ~11 kbp apart was used to calculate a DNA shearing index. Results were normalized to intact SHIV provirus copies per million CD4^+^ T cells. Detailed protocols for cell preparation, nucleic acid extraction, and digital PCR parameters are provided in Supplemental Methods.

### Statistics

Survival curves were generated using the Kaplan-Meier method, and the statistical significance between groups was assessed using the log-rank test. The correlation between rh-eCD4-Ig expression levels and the number of challenges needed for infection was determined by calculating Pearson’s correlation coefficient (*r*) and the coefficient of determination (*R*²). The statistical significance of the correlation was assessed using a 2-tailed test. Statistical significance between viral loads, days to rebound post-ATI, and intact provirus measurements of treatment groups was determined using the Kruskal-Wallis test, with *P* < 0.05 considered statistically significant. Statistical analysis of the AUC of the log_10_-transformed PVL was performed using the Kruskal-Wallis test with Dunn’s multiple-comparison test to compare treatment groups with the control group. Unless otherwise noted, data are shown as individual values for each study animal. Data in Figure 2, C–E, are presented as mean ± range.

### Study approval

PK profiling of ciapavir in rhesus macaques was conducted at the California National Primate Research Center (CNPRC) and approved in advance by the University of California, Davis, Institutional Animal Care and Use Committee (approval number 21373). The University of California, Davis, has an animal welfare assurance on file with the National Institutes of Health Office of Laboratory Animal Welfare (NIH-OLAW) and is fully accredited by the AAALAC. Likewise, experiments involving SHIV were approved by the Institutional Animal Care and Use Committees of the Fred Hutchinson Cancer Center/University of Washington (protocol no. 3235-04). Both institutions are AAALAC-accredited and maintain animal welfare assurance documentation through NIH-OLAW.

Rhesus macaques were housed and cared for under conditions that meet NIH standards as stated in the *Guide for the Care and Use of Laboratory Animals* (National Research Council, National Academies Press, Washington, DC, 1996), ILAR recommendations, and AAALAC accreditation standards, as described previously (53).

### Data availability

All data shown in figures are reported in the Supporting Data Values file. Next-generation sequencing data of Env sequences are available in GenBank under accession numbers PX651481–PX651713.

## Author contributions

LP, PT, DJHOC, MRG, MF, KRJ, SKC, HPK, and CWP designed the experiments. TE, LS, IL, AAK, PT, and WLWC performed experiments. WLWC and DJHOC conducted PK studies. TE and LS processed all samples and performed PVL- and PBMC-associated SHIV DNA and RNA assays. LP, NND, DJHOC, MRG, and CWP analyzed the data. OH and LP performed statistical analyses. DH synthesized ciapavir. KJB produced infectious SHIV.CH848 and designed the IR SHIV challenge experiment. JKB, LMK, ACN, TE, and HZ optimized and performed SHIV 5T-IPDA assays, which were designed by LP, ACPO, KRJ, and CWP. CMF performed Env sequencing assays, which were designed and analyzed by CMF, BFK, MRG, and CWP. LP, DJHOC, MRG, and CWP wrote the manuscript. LP, NDPC, MF, MRG, KRJ, SKC, HPK, and CWP supervised the studies.

## Funding support

This work is the result of NIH funding, in whole or in part, and is subject to the NIH Public Access Policy. Through acceptance of this federal funding, the NIH has been given a right to make the work publicly available in PubMed Central.

- NIH/NIAID (UM1 AI126623 to KRJ and HPK).
- NIH/NIAID (R01 AI167004 and R01 AI170214 to CWP).
- NIH/NIAID (UM1 AI164561 to LP, NDPC, and SKC).
- NIH/NIAID (R00 AI138860 to MRG).
- NIH/NIAID (P01 AI178375 to DJHOC).
- NIH/NIDA (R01 DA056770 to MRG).
- NIH/NIMH (R01 MH128155 to KJB).
- NIH/ORIP (P51 OD010425 and U42 OD011123 to the Washington National Primate Research Center).
- NIH/ORIP (P51 OD011107 supporting the CNPRC and its Flow Cytometry Core).
- National Cancer Institute, NIH (75N91019D00024).

## Supplementary Material

Supplemental data

Supporting data values

## Version 1 03/16/2026

Electronic publication
