# Supplementary material for: Smac mimetic combined with eCD4-Ig reverses latency without reducing SHIV reservoirs in rhesus macaques
Source: J Clin Invest. 2026 Mar 16;136(6):e187961. doi: 10.1172/JCI187961 (PMC12987619; doi:10.1172/JCI187961)
Supplement: Supplemental data [file jci-136-187961-s184.pdf]

Supplemental Methods

Env Sequencing

SHIV envelope was sequenced by single genome amplification as previously described in (1). In brief, viral RNA was isolated from blood plasma using QIAmp Viral RNA Mini Kit (Qiagen), and cDNA was generated using SuperScript IV reverse transcriptase (ThermoFisher) and the gene specific primer 5'-TGTAATAAATCCCTTCCAGTCCCCCC-3'. cDNA was subjected to limiting dilution PCR such that each reaction contained a single viral cDNA template as determined by Poisson statistics (2, 3). Nested PCR was performed to amplify the entire env gene using gene specific primers (round 1 (5'-CCTCCCCCTCCAGGACTAGC-3') and (5'-TGTAATAAATCCCTTCCAGTCCCCCC-3'); round 2 (5'-GACCTCCAGAAAATGAAGGACCAC-3') and (5'-ATGAGACATRTCTATTGCCAATTTGTA-3') and Platinum Taq High Fidelity Polymerase (ThermoFisher) per manufacturer's instructions. PCR amplicons were visualized by gel electrophoresis and correctly sized amplicons were sequenced directly with BigDye Terminator technology (ThermoFisher) and Sanger sequencing using HIV env specific primers. Resultant chromatograms were manually examined using Sequencher (GeneCodes) software, and sequences determined to have derived from the presence more than one viral template in the PCR were removed from the resultant dataset.

Cell Preparation for SHIV 5T-IPDA Assay

$CD4^+$ cells were isolated by bead-based positive selection (Stemcell Technologies, Vancouver, Canada) from leukapheresis samples collected at 2 time-points before and after treatment with the candidate anti-HIV effector and latency reversal therapies. The pre-and post-treatment leukapheresis collections occurred 18-22 and 69-73-weeks following ART initiation, respectively. The post-treatment samples were collected no sooner than 6 weeks following the end of the reservoir targeting phase and 1-3 weeks prior to ART treatment interruption (ATI). Where there was sufficient leukapheresis product available, triplicate CD4 cell purifications were performed. Quantities were verified using an automated cell counter (Cat. No AMQAF1000, Thermo Fisher Scientific, Waltham, MA). Flow cytometry was used to confirm the purity (>95%) of each $CD4^+$ cell product.

Nucleic Acid Extraction for SHIV 5T-IPDA Assay

Following CD4 isolations, genomic DNA (gDNA) was extracted using a previously published method (4), restriction enzyme-digested, and ethanol precipitated. The extraction method was optimized for an IPDA-specific process to minimize the shearing of gDNA. Briefly, 100 µl of 3 M guanidine HCl and 20 µL of Proteinase K (Cat. No. 19134, Qiagen, Hilden, Germany) was added to pellets containing 2-3 million cells, inverted 5 times, and incubated on a heat block at 56 °C for 1 h. Next, 400 µl of 6 M guanidinium isothiocyanate was added, inverted gently to mix, and incubated at 56 °C for 10 min. To precipitate gDNA, 500 µl of 100% isopropanol was added, tubes were inverted 10 times, and samples were centrifuged at 21000 x g for 10 min. Supernatants were carefully removed leaving gDNA pellets and approximately 100 µl of isopropanol; pellets were then washed with 1000 µl of 70% ethanol, inverted 10 times, and centrifuged again at 21000 x g for 3 min. The supernatant was completely removed by pipetting without disturbing the pellet, spun down at 21000 x g for 2 min to remove any residual supernatant, then air dried for 5 min. The gDNA pellets were eluted in 300 µl elution buffer (Cat. No. 19086, Qiagen), incubated at 60 °C for 1 hr then incubated at room temperature (RT) overnight. Restriction enzyme digestion of the genomic DNA was completed to aid in efficient dPCR droplet formation. The *Bgl*I recognition sequence targets outer regions of sites containing integrated SHIV genomes and reference DNA sequences (5). Thirty-five microliters of 10x 3.1 buffer and 13.2 µl *Bgl*I enzyme (Cat No. R0143L, NEB) were added to each tube and inverted to mix. Samples were incubated overnight in a shaking thermomixer at 37 °C at 300 RPM. An additional 5 µl of Bgl1 enzyme was subsequently added and incubation was repeated for 1 hr. An ethanol-based precipitation protocol was applied to purify digested gDNA (6). Briefly, 35 microliters of 3 M sodium acetate and 766 µl of 100% ice-cold ethanol were added to each sample, mixed by inverting, and incubated on ice for 30 min, inverting the tubes every 10 min. After centrifuging at 21000 x g for 10 min, supernatants were removed to 100 µl volume, 1000 µl of 70% ethanol was added, and tubes were centrifuged at 21000 x g for 3 min. The supernatant was completely removed, and the cell pellet was air dried for 5 min. Digested gDNA was eluted with 50 µl of elution buffer, incubated at 60 °C for 1 hr, then incubated overnight at RT. DNA concentration was measured using a nanodrop (Cat. No. NDNDLUSCAN, Thermo Scientific), aiming for concentrations between 200-300 ng per µl. If gDNA concentration was >300 ng, a repeated *Bgl*I digest and ethanol precipitation was performed.

SHIV 5T-IPDA Assay Digital PCR and Data Analysis

Quantitation of intact proviral DNA copies was established by creating a multiplexed assay, which can simultaneously measure 5 target regions within the SHIV genome [Group-specific antigen (gag), long-terminal repeat (LTR), envelope (env), polymerase (pol), and trans-activator of transcription (tat)]. DNA was assayed using the primer/probe sequences listed in **Supplemental Table 3**, which were designed comparatively to an in-house HIV IPDA dPCR assay. Droplets that were quintuple positive for all 5 targets were accepted as intact proviruses. To account for undercounting of intact proviral DNA due to DNA shearing during the extraction process (5), a reference assay was developed to target the Macaque RNase P p30 gene (MRPP30), a highly conserved housekeeping gene. 5'MRPP30 and 3'MRPP30 targets were spaced ~11 kbp apart to approximate the length of intact SHIV proviral genomes. Double positive droplets were accepted as intact DNA. Probability of template shearing was calculated using a DNA shearing index (DSI) against single and double-positive droplets for correction and accuracy of measuring intact DNA. $CD4^+$ T-cell DNA was distinguished from non-$CD4^+$ T-cell DNA by using an MDeltaD assay to amplify a gene that is excised during receptor gene rearrangement in maturing T-cells (5). Total cell copies in each sample were subtracted from non-T-cell copies and normalized to 1 million T-cells per reaction. Droplet PCR was performed on the Bio-Rad QX600 Droplet Digital PCR System for target detection, using manufacturer supplied materials and ddPCR 4x multiplex supermix (Cat. No. 12005911, Bio-Rad, Hercules, CA). For SHIV proviral reactions, 10 µl of DNA was aliquoted into each reaction well with a total volume of 25 µl and each sample was run in triplicate wells to ensure consistent quantitation. Replicate wells were merged during analysis to create a dynamic range of values. For reference reactions measuring DNA shearing, extracted DNA was diluted 1:50 and ran in a single-well reaction per sample. Each type of reaction ran on the same plate simultaneously. PCR was performed with the following settings: 95 °C for 10 min, 40 cycles of: 94 °C for 30 sec, 60 °C for 1 min, and 98 °C for 10 min with a 2 °C ramp rate. Uninfected $CD4^+$ T-cells were processed in parallel, and the extracted DNA was used as a negative control along with commercially isolated rhesus macaque genomic DNA (Cat. No. D1534999-G01, BioChain, Newark, CA), and NTC replicate wells to confirm no PCR contamination. Positive controls consisted of a linearized plasmid containing all 5 SHIV targets, spiked with commercial rhesus macaque background DNA and a secondary positive control made of 5 individual gBlocks of the same concentration, representing each SHIV target. During analysis, controls were used for setting a threshold and each target was set at an individual threshold and kept consistent to avoid inter-assay variation. Each of the singlicate, duplicate, or triplicate CD4 purifications (replicates based on leukapheresis cell yield) were amplified in triplicate wells in the IPDA dPCR assay. If the accepted droplet count was lower than 10,000 for 1 or 2 wells, the values were excluded but the values for the remaining wells of the sample were considered. If all 3 triplicate wells were below 10,000 droplets, the corresponding CD4-purified sample was wholly excluded. Omitted data points either correspond to failed/inefficient leukapheresis (insufficient cells available for analysis) or failed dPCR due to suboptimal droplet formation. Values represent the average of triplicate wells for a single CD4 purification and an average of up to 3 technical replicate CD4 purifications per animal per time point. Data is plotted as intact SHIV provirus copies per Million CD4+ PBMC and for animals with both pre- and post-treatment values as the $\log_{10}$ ratio of those values.

Supplemental Figures

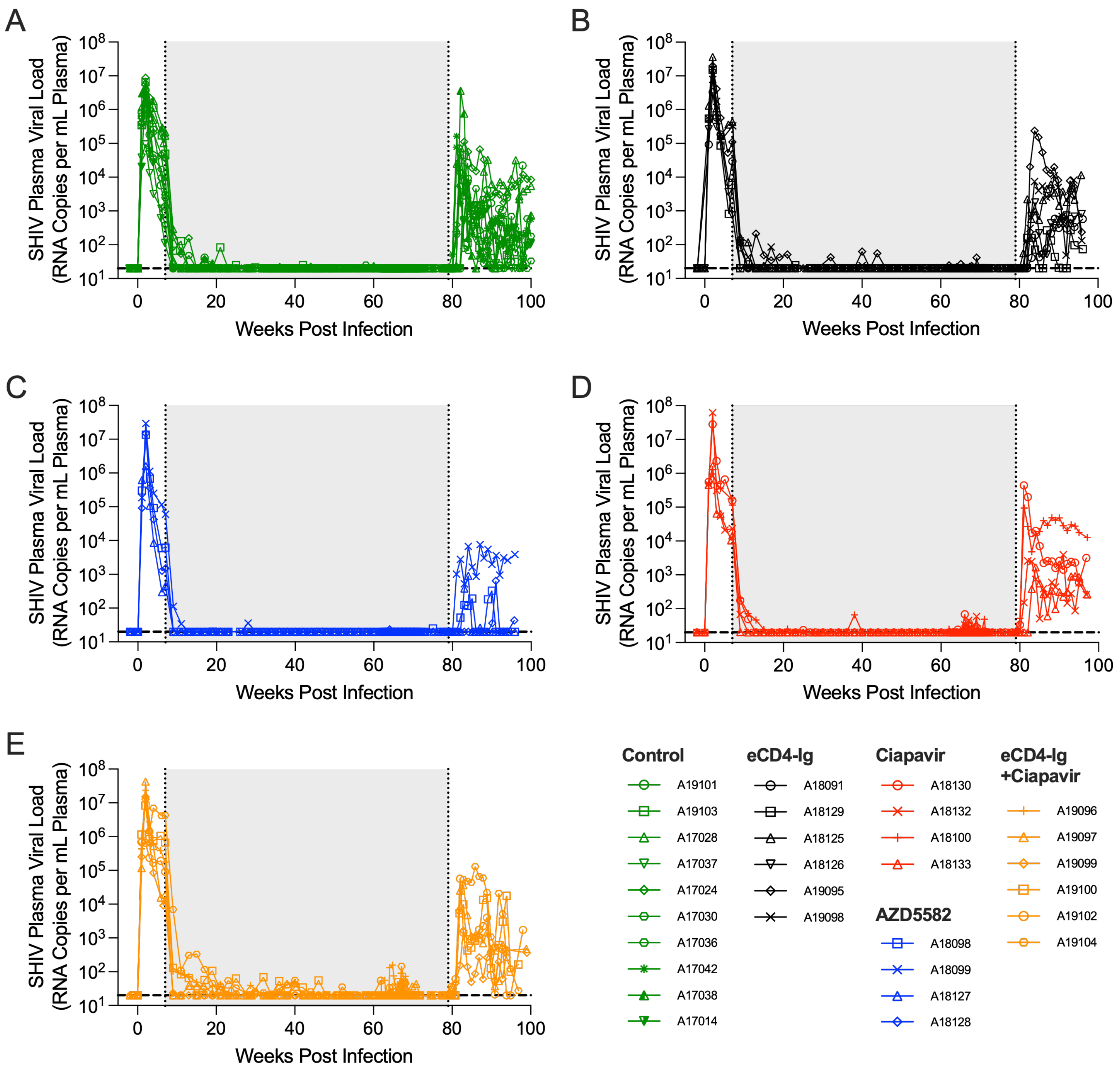

Supplemental Figure 1: Plasma viral load in SHIV-infected animals treated with ART only (negative control) **(A)**, eCD4-Ig **(B)**, AZD5582 **(C)**, Ciapavir **(D),** or Ciapavir in combination with eCD4-Ig **(E)**. Dotted vertical lines indicate start and end of ART, and area shaded in gray represents the period of ART administration. The level of quantification (LOQ) is indicated by a black dashed line.

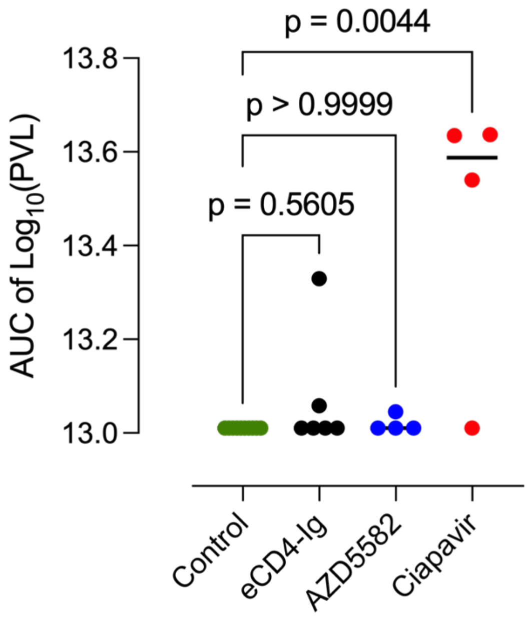

Supplemental Figure 2: Area under the curve (AUC) analysis of log_{10}(PVL) levels withing the LRA treatment period (weeks 61 to 72). Statistical analysis was performed using the Kruskal-Wallis test with Dunn's multiple comparison correction to compare treatment groups with the control group. Black line indicates median. Statistical significance was defined as $p < 0.05$.

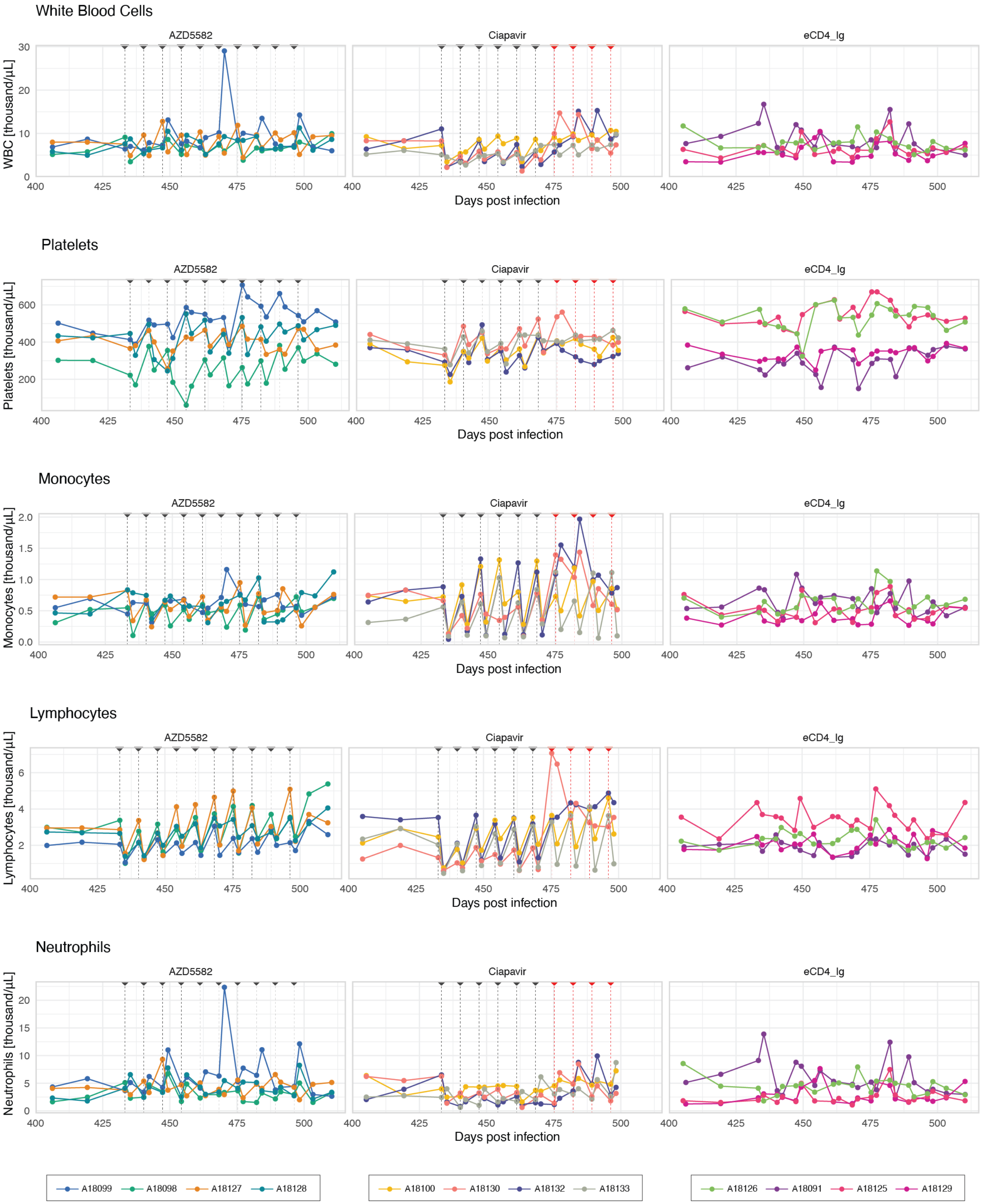

Supplemental Figure 3: Complete blood counts (CBC) were conducted at the day of LRA administration and 2 days post dosing. LRA administration is indicated by grey triangles/lines. Red triangles/lines represent LRA administration to animals A18100 and A18133 only (see Figure 6).

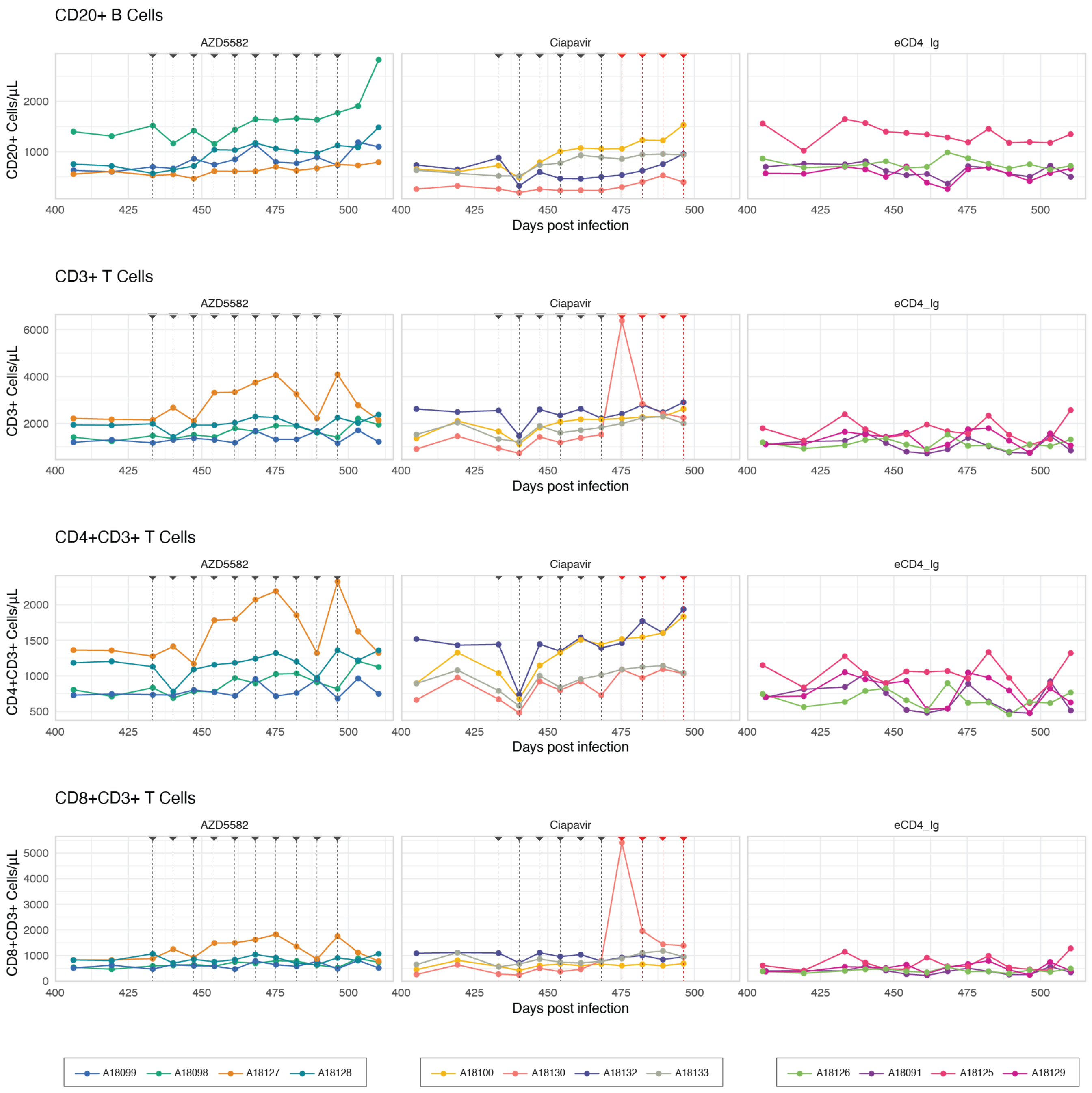

Supplemental Figure 4: Lymphocyte subset analyses were conducted on the day of LRA administration. LRA administration is indicated by grey triangles/lines. Red triangles/lines represent LRA administration to animals A18100 and A18133 only (see Figure 6).

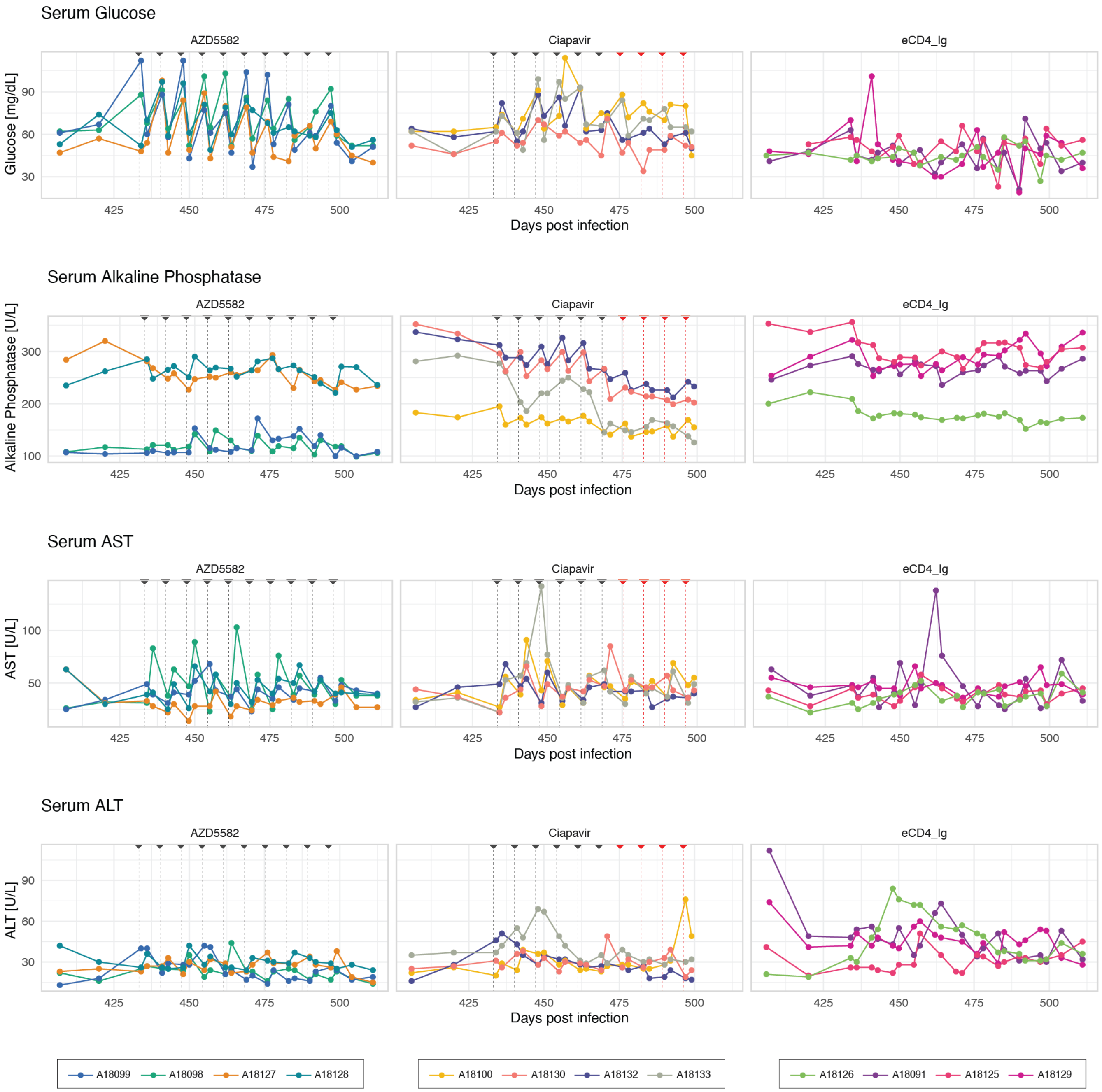

Supplemental Figure 5: Serum chemistry measurements for comparison to ((7)), conducted on the day of LRA administration and 2 days post dosing. LRA administration is indicated by grey triangles/lines. Red triangles/lines represent LRA administration to animals A18100 and A18133 only (see Figure 6).

		Apex				Base of V3 Loop									V3 Crown				CD4 Binding Loop							V4 Region					
Animal ID	Total Sequences	K168	K169	Q170	K171	T297	R298	P299	N300	N301	N302	T303	R304	K305	G312	P313	G314	Q315	S364	S365	G366	G367	D368	L369	E370	F382	F383	Y384	C385	N386	T387
**eCD4-Ig**																															
A19095	39	0	0	0	0	0	0	0	0	0	0	0	0	0	0	0	0	0	0	0	0	0	0	0	0	0	0	0	0	0	0
A19098	25	0	0	0	0	0	0	0	0	0	0	0	0	0	0	0	0	0	0	0	0	0	0	0	0	0	0	0	0	0	0
**eCD4-Ig + Ciapavir**																															
A19097	21	0	0	0	0	0	0	0	0	0	0	0	0	0	0	0	0	0	0	0	0	0	0	0	0	0	0	0	0	0	0
A19100	37	0	0	0	0	0	0	5.4	0	0	0	0	0	0	0	0	0	2.7	8.1	0	0	0	0	0	0	0	0	0	0	2.7	0
A19102	17	0	0	0	0	0	0	0	0	0	0	0	0	0	0	0	0	0	0	0	0	0	0	0	0	0	0	0	0	0	0
A19104	28	0	0	3.6	0	0	0	0	0	0	0	0	0	0	0	0	0	3.6	0	0	0	0	0	0	0	0	0	0	0	0	0
**Control**																															
A19101	29	0	0	0	0	0	0	0	0	0	0	0	0	0	0	0	0	0	0	0	0	0	0	0	0	0	0	0	0	100	0
A19103	35	0	0	0	0	0	0	0	0	0	0	0	0	0	0	0	0	0	0	0	0	0	0	0	0	0	0	0	2.9	0	0

Supplemental Figure 6: SHIV-1157ipd3N4 Env sequence analysis after viral rebound. Full-length SHIV-1157ipd3N4 Env sequences from eight rhesus macaques were analyzed after viral rebound post-analytical treatment interruption (ATI). Treatment groups from which sequences were derived are indicated for each animal. Regions of interest based on previously identified resistance mutations to rh-eCD4-Ig are highlighted with numbered amino acid positions. Amino acid change frequencies are represented for each position based on the total number of Env sequencing reads obtained. Color coding indicates conservation relative to the wild-type SHIV-1157ipd3N4 Env sequence, where blue represents high conservation and red represents low conservation (higher mutation frequency).

Supplemental Tables

Study Group	ID	Sex
eCD4-Ig	A18091	M
	A18129	F
	A18125	F
	A18126	F
	A19095	M
	A19098	M
Ciapavir	A18130	M
	A18132	M
	A18100	M
	A18133	M
AZD5582	A18098	M
	A18099	M
	A18127	F
	A18128	F
eCD4-Ig + Ciapavir	A19100	M
	A19096	M
	A19097	M
	A19102	M
	A19104	M
	A19099	M
Control	A19101	M
	A19103	M
	A17028	M
	A17024	M
	A17036	M
	A17038	M
	A17037	M
	A17030	M
	A17042	M
	A17014	M

Supplemental Table 1: Study groups of rhesus macaques

A19095 eCD4-Ig

```
                       SHIV1157 MRVKEKYQHLWRWGWRWGIM LLGMLMICSASEKLWVTVYY GVPVWKEAKTTLFCASNAKA YEKEVHNIWATHACVPTDPN PQEIVLGNVTENFNMWKNDM  100
 A19095.DAY_552.PlasmacDNA.P4B5 -------------------- -------------------- -------------------- -------------------- --------------------
 A19095.DAY_552.PlasmacDNA.P4B6 -------------------- -------------------- -------------------- -------------------- --------------------
 A19095.DAY_552.PlasmacDNA.P4B7 -------------------- -------------------- -------------------- -------------------- --------------------
 A19095.DAY_552.PlasmacDNA.P4B8 -------------------- -------------------- -------------------- -------------------- --------------------
 A19095.DAY_552.PlasmacDNA.P4B9 -------------------- -------------------- -------------------- -------------------- --------------------
A19095.DAY_552.PlasmacDNA.P4B10 -------------------- -------------------- -------------------- -------------------- --------------------
A19095.DAY_552.PlasmacDNA.P4B12 -------------------- -------------------- -------------------- -------------------- --------------------
A19095.DAY_552.PlasmacDNA.P4B13 -------------------- -------------------- -------------------- -------------------- --------------------
A19095.DAY_552.PlasmacDNA.P4B14 -------------------- -------------------- -------------------- -------------------- --------------------
A19095.DAY_552.PlasmacDNA.P4B16 -------------------- -------------------- -------------------- -------------------- --------------------
A19095.DAY_552.PlasmacDNA.P4B18 -------------------- -------------------- -------------------- -------------------- --------------------
A19095.DAY_552.PlasmacDNA.P4B19 -------------------- -------------------- -------------------- -------------------- --------------------
A19095.DAY_552.PlasmacDNA.P4B20 -------------------- -------------------- -------------------- -------------------- --------------------
A19095.DAY_552.PlasmacDNA.P4B21 -------------------- -------------------- -------------------- -------------------- --------------------
A19095.DAY_552.PlasmacDNA.P4B22 -------------------- -------------------- -------------------- -------------------- --------------------
A19095.DAY_552.PlasmacDNA.P4B23 -------------------- -------------------- -------------------- -------------------- --------------------
A19095.DAY_552.PlasmacDNA.P4B24 -------------------- -------------------- -------------------- -------------------- --------------------
 A19095.DAY_552.PlasmacDNA.P4C1 -------------------- -------------------- -------------------- -------------------- --------------------
 A19095.DAY_552.PlasmacDNA.P4C2 -------------------- -------------------- -------------------- -------------------- --------------------
 A19095.DAY_552.PlasmacDNA.P4C3 -------------------- -------------------- -------------------- -------------------- --------------------
 A19095.DAY_552.PlasmacDNA.P4C4 -------------------- -------------------- -------------------- -------------------- --------------------
 A19095.DAY_552.PlasmacDNA.P4C5 -------------------- -------------------- -------------------- -------------------- --------------------
 A19095.DAY_552.PlasmacDNA.P4C6 -------------------- -------------------- -------------------- -------------------- --------------------
 A19095.DAY_552.PlasmacDNA.P4C9 -------------------- -------------------- -------------------- -------------------- --------------------
A19095.DAY_552.PlasmacDNA.P4C10 -------------------- -------------------- -------------------- -------------------- --------------------
A19095.DAY_552.PlasmacDNA.P4C11 -------------------- -------------------- -------------------- -------------------- --------------------
A19095.DAY_552.PlasmacDNA.P4C12 -------------------- -------------------- -------------------- -------------------- --------------------
A19095.DAY_657.PlasmacDNA.P3A13 -------------------- -------------------- -------------------- -------------------- --------------------
A19095.DAY_657.PlasmacDNA.P3B23 -------------------- -------------------- -------------------- -------------------- --------------------
 A19095.DAY_657.PlasmacDNA.P4C9 -------------------- -------------------- -------------------- -------------------- --------------------
 A19095.DAY_657.PlasmacDNA.P4D6 -------------------- -------------------- -------------------- -------------------- --------------------
 A19095.DAY_657.PlasmacDNA.P4D7 -------------------- -------------------- -------------------- -------------------- --------------------
A19095.DAY_657.PlasmacDNA.P4D24 -------------------- -------------------- -------------------- -------------------- --------------------
 A19095.DAY_657.PlasmacDNA.P4E7 -------------------- -------------------- -------------------- -------------------- --------------------
A19095.DAY_657.PlasmacDNA.P4E15 -------------------- -------------------- -------------------- -------------------- --------------------
 A19095.DAY_657.PlasmacDNA.P4F6 -------------------- -------------------- -------------------- -------------------- --------------------
 A19095.DAY_657.PlasmacDNA.P4H2 -------------------- -------------------- -------------------- -------------------- --------------------
 A19095.DAY_657.PlasmacDNA.P4H7 -------------------- -------------------- -------------------- -------------------- --------------------
 A19095.DAY_657.PlasmacDNA.P4H8 -------------------- -------------------- -------------------- -------------------- --------------------

                       SHIV1157 VDQMHEDIISLWDQSLKPCV KLTSLCVTLKCSNFTGKSNV TYKGDMEVKNCSFNVTTEIR DKKQKVYALFYRLDITPLDD NSSEYILINCNSSTITQACP  200
 A19095.DAY_552.PlasmacDNA.P4B5 -------------------- -------------------- -------------------- -------------------- --------------------
 A19095.DAY_552.PlasmacDNA.P4B6 -------------------- -------------------- -------------------- -------------------- --------------------
 A19095.DAY_552.PlasmacDNA.P4B7 -------------------- -------------------- -------------------- -------------------- --------------------
 A19095.DAY_552.PlasmacDNA.P4B8 -------------------- -------------------- -------------------- -------------------- --------------------
 A19095.DAY_552.PlasmacDNA.P4B9 -------------------- -------------------- -------------------- -------------------- --------------------
A19095.DAY_552.PlasmacDNA.P4B10 -------------------- -------------------- -------------------- -------------------- --------------------
A19095.DAY_552.PlasmacDNA.P4B12 -------------------- -------------------- -------------------- -------------------- --------------------
A19095.DAY_552.PlasmacDNA.P4B13 -------------------- -------------------- -------------------- -------------------- --------------------
A19095.DAY_552.PlasmacDNA.P4B14 -------------------- -------------------- -------------------- -------------------- --------------------
A19095.DAY_552.PlasmacDNA.P4B16 -------------------- -------------------- -------------------- -------------------- --------------------
A19095.DAY_552.PlasmacDNA.P4B18 -------------------- -------------------- -------------------- -------------------- --------------------
A19095.DAY_552.PlasmacDNA.P4B19 -------------------- -------------------- -------------------- -------------------- --------------------
A19095.DAY_552.PlasmacDNA.P4B20 -------------------- -------------------- -------------------- -------------------- --------------------
A19095.DAY_552.PlasmacDNA.P4B21 -------------------- -------------------- -------------------- -------------------- --------------------
A19095.DAY_552.PlasmacDNA.P4B22 -------------------- -------------------- -------------------- -------------------- --------------------
A19095.DAY_552.PlasmacDNA.P4B23 -------------------- -------------------- -------------------- -------------------- --------------------
A19095.DAY_552.PlasmacDNA.P4B24 -------------------- -------------------- -------------------- -------------------- --------------------
 A19095.DAY_552.PlasmacDNA.P4C1 -------------------- -------------------- -------------------- -------------------- --------------------
 A19095.DAY_552.PlasmacDNA.P4C2 -------------------- -------------------- -------------------- -------------------- --------------------
 A19095.DAY_552.PlasmacDNA.P4C3 -------------------- -------------------- -------------------- -------------------- --------------------
 A19095.DAY_552.PlasmacDNA.P4C4 -------------------- -------------------- -------------------- -------------------- --------------------
 A19095.DAY_552.PlasmacDNA.P4C5 -------------------- -------------------- -------------------- -------------------- --------------------
 A19095.DAY_552.PlasmacDNA.P4C6 -------------------- -------------------- -------------------- -------------------- --------------------
 A19095.DAY_552.PlasmacDNA.P4C9 -------------------- -------------------- -------------------- -------------------- --------------------
A19095.DAY_552.PlasmacDNA.P4C10 -------------------- -------------------- -------------------- -------------------- --------------------
A19095.DAY_552.PlasmacDNA.P4C11 -------------------- -------------------- -------------------- -------------------- --------------------
A19095.DAY_552.PlasmacDNA.P4C12 -------------------- -------------------- -------------------- -------------------- --------------------
A19095.DAY_657.PlasmacDNA.P3A13 -------------------- -------------------- -------------------- -------------------- --------------------
A19095.DAY_657.PlasmacDNA.P3B23 -------------------- -------------------- -------------------- -------------------- --------------------
 A19095.DAY_657.PlasmacDNA.P4C9 -------------------- -------------------- ------------------V- -------------------- --------------------
 A19095.DAY_657.PlasmacDNA.P4D6 -------------------- -------------------- -------------------- -------------------- --------------------
 A19095.DAY_657.PlasmacDNA.P4D7 -------------------- -------------------- -------------------- -------------------- --------------------
A19095.DAY_657.PlasmacDNA.P4D24 -------------------- -------------------- -------------------- -------------------- --------------------
 A19095.DAY_657.PlasmacDNA.P4E7 -------------------- -------------------- -------------------- -------------------- --------------------
A19095.DAY_657.PlasmacDNA.P4E15 -------------------- -------------------- -------------------- -------------------- --------------------
 A19095.DAY_657.PlasmacDNA.P4F6 -------------------- -------------------- -------------------- -------------------- --------------------
 A19095.DAY_657.PlasmacDNA.P4H2 -------------------- -------------------- -------------------- -------------------- --------------------
 A19095.DAY_657.PlasmacDNA.P4H7 -------------------- -------------------- -------------------- -------------------- --------------------
 A19095.DAY_657.PlasmacDNA.P4H8 -------------------- -------------------- -------------------- -------------------- --------------------

                       SHIV1157 KVNFDPIPIHYCAPAGYAIL KCNNKTFNGTGPCHNVSTVQ CTHGIKPVVSTQLLLNGSLA EGEIIIRSENLTDNVKTIIV HFNESVEITCTRPNNNTRKS  300
 A19095.DAY_552.PlasmacDNA.P4B5 -------------------- -------------------- -------------------- -------------------- ---K----------------
 A19095.DAY_552.PlasmacDNA.P4B6 -------------------- -------------------- -------------------- -------------------- --------------------
 A19095.DAY_552.PlasmacDNA.P4B7 -------------------- -------------------- -------------------- -------------------- --------------------
 A19095.DAY_552.PlasmacDNA.P4B8 -------------------- -------------------- -------------------- -------------------- --------------------
 A19095.DAY_552.PlasmacDNA.P4B9 -------------------- -------------------- -------------------- -------------------- --------------------
A19095.DAY_552.PlasmacDNA.P4B10 -------------------- -------------------- -------------------- -------------------- --------------------
A19095.DAY_552.PlasmacDNA.P4B12 -------------------- -------------------- -------------------- -------------------- --------------------
A19095.DAY_552.PlasmacDNA.P4B13 -------------------- -------------------- -------------------- -------------------- --------------------
A19095.DAY_552.PlasmacDNA.P4B14 -------------------- -------------------- -------------------- -------------------- --------------------
A19095.DAY_552.PlasmacDNA.P4B16 -------------------- -------------------- -------------------- -------------------- --------------------
A19095.DAY_552.PlasmacDNA.P4B18 -------------------- -------------------- -------------------- -------------------- --------------------
A19095.DAY_552.PlasmacDNA.P4B19 -------------------- -------------------- -------------------- -------------------- --------------------
A19095.DAY_552.PlasmacDNA.P4B20 -------------------- -------------------- -------------------- -------------------- --------------------
A19095.DAY_552.PlasmacDNA.P4B21 -------------------- -------------------- -------------------- -------------------- --------------------
A19095.DAY_552.PlasmacDNA.P4B22 -------------------- -------------------- -------------------- -------------------- --------------------
A19095.DAY_552.PlasmacDNA.P4B23 -------------------- -------------------- -------------------- -------------------- --------------------
A19095.DAY_552.PlasmacDNA.P4B24 -------------------- -------------------- -------------------- -------------------- --------------------
 A19095.DAY_552.PlasmacDNA.P4C1 -------------------- -------------------- -------------------- -------------------- --------------------
 A19095.DAY_552.PlasmacDNA.P4C2 -------------------- -------------------- -------------------- -------------------- --------------------
 A19095.DAY_552.PlasmacDNA.P4C3 -------------------- -------------------- -------------------- -------------------- --------------------
 A19095.DAY_552.PlasmacDNA.P4C4 -------------------- -------------------- -------------------- -------------------- --------------------
```

```
 A19095.DAY_552.PlasmacDNA.P4C5 -------------------- -------------------- -------------------- -------------------- --------------------
 A19095.DAY_552.PlasmacDNA.P4C6 -------------------- -------------------- -------------------- -------------------- --------------------
 A19095.DAY_552.PlasmacDNA.P4C9 -------------------- -------------------- -------------------- -------------------- --------------------
A19095.DAY_552.PlasmacDNA.P4C10 -------------------- -------------------- -------------------- -------------------- --------------------
A19095.DAY_552.PlasmacDNA.P4C11 -------------------- -------------------- -------------------- -------------------- --------------------
A19095.DAY_552.PlasmacDNA.P4C12 -------------------- -------------------- -------------------- -------------------- --------------------
A19095.DAY_657.PlasmacDNA.P3A13 -------------------- -------------------- -------------------- -------------------- --------------------
A19095.DAY_657.PlasmacDNA.P3B23 -------------------- -------------------- -------------------- -------------------- --------------------
 A19095.DAY_657.PlasmacDNA.P4C9 -------------------- -------------------- -------------------- -------------------- --------------------
 A19095.DAY_657.PlasmacDNA.P4D6 -------------------- -------------------- -------------------- -------------------- --------------------
 A19095.DAY_657.PlasmacDNA.P4D7 -------------------- -------------------- -------------------- -------------------- --------------------
A19095.DAY_657.PlasmacDNA.P4D24 -------------------- -------------------- -------------------- -------------------- --------------------
 A19095.DAY_657.PlasmacDNA.P4E7 -------------------- -------------------- -------------------- -------------------- --------------------
A19095.DAY_657.PlasmacDNA.P4E15 -------------------- -------------------- -------------------- -------------------- --------------------
 A19095.DAY_657.PlasmacDNA.P4F6 -------------------- -------------------- -------------------- -------------------- --------------------
 A19095.DAY_657.PlasmacDNA.P4H2 -------------------- -------------------- -------------------- -------------------- --------------------
 A19095.DAY_657.PlasmacDNA.P4H7 -------------------- -------------------- -------------------- -------------------- --------------------
 A19095.DAY_657.PlasmacDNA.P4H8 -------------------- -------------------- -------------------- -------------------- --------------------

                       SHIV1157 ISIGPGQAIYATGDIIGDIR QAHCNISKENWNKTLQWVRG KLKEHFPNKTIVFKPSSGGD LEITTHSFNCRGEFFYCNTS KLFNSTDNSTHMGTENNTII  400
 A19095.DAY_552.PlasmacDNA.P4B5 -------------------- -------------------- -------------------- -------------------- --------------------
 A19095.DAY_552.PlasmacDNA.P4B6 -------------------- -------------------- -------------------- -------------------- --------------------
 A19095.DAY_552.PlasmacDNA.P4B7 -------------------- -------------------- -------------------- -------------------- --------------------
 A19095.DAY_552.PlasmacDNA.P4B8 -------------------- -------------------- -------------------- -------------------- --------------------
 A19095.DAY_552.PlasmacDNA.P4B9 -------------------- -------------------- -------------------- -------------------- --------------------
A19095.DAY_552.PlasmacDNA.P4B10 -------------------- -------------------- -------------------- -------------------- --------------------
A19095.DAY_552.PlasmacDNA.P4B12 -------------------- -------------------- -------------------- -------------------- --------------------
A19095.DAY_552.PlasmacDNA.P4B13 -------------------- -------------------- -------------------- -------------------- --------------------
A19095.DAY_552.PlasmacDNA.P4B14 -------------------- -------------------- -------------------- -------------------- --------------------
A19095.DAY_552.PlasmacDNA.P4B16 -------------------- -------------------- -------------------- -------------------- --------------------
A19095.DAY_552.PlasmacDNA.P4B18 -------------------- -------------------- -------------------- -------------------- --------------------
A19095.DAY_552.PlasmacDNA.P4B19 -------------------- -------------------- -------------------- -------------------- --------------------
A19095.DAY_552.PlasmacDNA.P4B20 -------------------- -------------------- -------------------- -------------------- --------------------
A19095.DAY_552.PlasmacDNA.P4B21 -------------------- -------------------- -------------------- -------------------- --------------------
A19095.DAY_552.PlasmacDNA.P4B22 -------------------- -------------------- -------------------- -------------------- --------------------
A19095.DAY_552.PlasmacDNA.P4B23 -------------------- -------------------- -------------------- -------------------- --------------------
A19095.DAY_552.PlasmacDNA.P4B24 -------------------- -------------------- -------------------- -------------------- --------------------
 A19095.DAY_552.PlasmacDNA.P4C1 -------------------- -------------------- -------------------- -------------------- --------------------
 A19095.DAY_552.PlasmacDNA.P4C2 -------------------- -------------------- -------------------- -------------------- --------------------
 A19095.DAY_552.PlasmacDNA.P4C3 -------------------- -------------------- -------------------- -------------------- --------------------
 A19095.DAY_552.PlasmacDNA.P4C4 -------------------- -------------------- -------------------- -------------------- --------------------
 A19095.DAY_552.PlasmacDNA.P4C5 -------------------- -------------------- -------------------- -------------------- --------------------
 A19095.DAY_552.PlasmacDNA.P4C6 -------------------- -------------------- -------------------- -------------------- --------------------
 A19095.DAY_552.PlasmacDNA.P4C9 -------------------- -------------------- -------------------- -------------------- --------------------
A19095.DAY_552.PlasmacDNA.P4C10 -------------------- -------------------- -------------------- -------------------- --------------------
A19095.DAY_552.PlasmacDNA.P4C11 -------------------- -------------------- -------------------- -------------------- --------------------
A19095.DAY_552.PlasmacDNA.P4C12 -------------------- -------------------- -------------------- -------------------- --------------------
A19095.DAY_657.PlasmacDNA.P3A13 -------------------- -------------------- -------------------- -------------------- --------------------
A19095.DAY_657.PlasmacDNA.P3B23 -------------------- -------------------- -------------------- -------------------- --------------------
 A19095.DAY_657.PlasmacDNA.P4C9 -------------------- -------------------- -------------------- -------------------- --------------------
 A19095.DAY_657.PlasmacDNA.P4D6 -------------------- -------------------- -------------------- -------------------- --------------------
 A19095.DAY_657.PlasmacDNA.P4D7 -------------------- -------------------- -------------------- -------------------- --------------------
A19095.DAY_657.PlasmacDNA.P4D24 -------------------- -------------------- -------------------- -------------------- --------------------
 A19095.DAY_657.PlasmacDNA.P4E7 -------------------- -------------------- -------------------- -------------------- --------------------
A19095.DAY_657.PlasmacDNA.P4E15 -------------------- -------------------- -------------------- -------------------- --------------G-----
 A19095.DAY_657.PlasmacDNA.P4F6 -------------------- -------------------- -------------------- -------------------- --------------------
 A19095.DAY_657.PlasmacDNA.P4H2 -------------------- -------------------- -------------------- -------------------- --------------------
 A19095.DAY_657.PlasmacDNA.P4H7 -------------------- -------------------- -------------------- -------------------- --------------------
 A19095.DAY_657.PlasmacDNA.P4H8 -------------------- -------------------- -------------------- -------------------- -------........-----

                       SHIV1157 TIPCRIKQIINMWQEVGRAM YAPPIEGNITCKSNITGLLL VRDGGWDNSTNDTETFRPGG GDMRDNWRSELYKYKVVEVK PLGIAPTKAKRRVVEREKRA  500
 A19095.DAY_552.PlasmacDNA.P4B5 -------------------- -------------------- ------G------------- -------------------- --------------------
 A19095.DAY_552.PlasmacDNA.P4B6 -------------------- -------------------- ------G------------- -------------------- --------------------
 A19095.DAY_552.PlasmacDNA.P4B7 -------------------- -------------------- ------G------------- -------------------- --------------------
 A19095.DAY_552.PlasmacDNA.P4B8 -------------------- -------------------- ------G------------- -------------------- --------------------
 A19095.DAY_552.PlasmacDNA.P4B9 -------------------- -------------------- ------G------------- -------------------- --------------------
A19095.DAY_552.PlasmacDNA.P4B10 -------------------- -------------------- ------G------------- -------------------- --------------------
A19095.DAY_552.PlasmacDNA.P4B12 -------------------- -------------------- ------G------------- -------------------- --------------------
A19095.DAY_552.PlasmacDNA.P4B13 -------------------- -------------------- ------G------------- -------------------- --------------------
A19095.DAY_552.PlasmacDNA.P4B14 -------------------- -------------------- ------G------------- -------------------- --------------------
A19095.DAY_552.PlasmacDNA.P4B16 -------------------- -------------------- ------G------------- -------------------- --------------------
A19095.DAY_552.PlasmacDNA.P4B18 -------------------- -------------------- ------G------------- -------------------- --------------------
A19095.DAY_552.PlasmacDNA.P4B19 -------------------- -------------------- ------G------------- -------------------- --------------------
A19095.DAY_552.PlasmacDNA.P4B20 -------------------- -------------------- ------G------------- -------------------- --------------------
A19095.DAY_552.PlasmacDNA.P4B21 -------------------- -------------------- ------G------------- -------------------- --------------------
A19095.DAY_552.PlasmacDNA.P4B22 -------------------- -------------------- ------G------------- -------------------- --------------------
A19095.DAY_552.PlasmacDNA.P4B23 -------------------- -------------------- ------G------------- -------------------- --------------------
A19095.DAY_552.PlasmacDNA.P4B24 -------------------- -------------------- ------G------------- -------------------- --------------------
 A19095.DAY_552.PlasmacDNA.P4C1 -------------------- -------------------- ------G------------- -------------------- --------------------
 A19095.DAY_552.PlasmacDNA.P4C2 -------------------- -------------------- ------G------------- -------------------- --------------------
 A19095.DAY_552.PlasmacDNA.P4C3 -------------------- -------------------- ------G------------- -------------------- --------------------
 A19095.DAY_552.PlasmacDNA.P4C4 -------------------- -------------------- ------G------------- -------------------- --------------------
 A19095.DAY_552.PlasmacDNA.P4C5 -------------------- -------------------- ------G------------- -------------------- --------------------
 A19095.DAY_552.PlasmacDNA.P4C6 -------------------- -------------------- ------G------------- -------------------- --------------------
 A19095.DAY_552.PlasmacDNA.P4C9 -------------------- -------------------- ------G------------- -------------------- --------------------
A19095.DAY_552.PlasmacDNA.P4C10 -------------------- -------------------- ------G------------- -------------------- --------------------
A19095.DAY_552.PlasmacDNA.P4C11 -------------------- -------------------- ------G------------- -------------------- --------------------
A19095.DAY_552.PlasmacDNA.P4C12 -------------------- -------------------- ------G------------- -------------------- --------------------
A19095.DAY_657.PlasmacDNA.P3A13 -------------------- -------------------- -------------------- -------------------- --------------------
A19095.DAY_657.PlasmacDNA.P3B23 -------------------- -------------------- -------------------- -------------------- --------------------
 A19095.DAY_657.PlasmacDNA.P4C9 -------------------- -------------------- -------------------- -------------------- --------------------
 A19095.DAY_657.PlasmacDNA.P4D6 -------------------- -------------------- -------------------- -------------------- --------------------
 A19095.DAY_657.PlasmacDNA.P4D7 -------------------- -------------------- -------------------- -------------------- --------------------
A19095.DAY_657.PlasmacDNA.P4D24 -------------------- -------------------- ------G------------- -------------------- --------------------
 A19095.DAY_657.PlasmacDNA.P4E7 -------------------- -------------------- -------------------- -------------------- --------------------
A19095.DAY_657.PlasmacDNA.P4E15 -------------------- -------------------- ------G------------- -------------------- --------------------
 A19095.DAY_657.PlasmacDNA.P4F6 -------------------- -------------------- -------------------- -------------------- --------------------
 A19095.DAY_657.PlasmacDNA.P4H2 -------------------- -------------------- -------------------- -------------------- --------------------
 A19095.DAY_657.PlasmacDNA.P4H7 -------------------- -------------------- -------------------- R------------------- --------------------
 A19095.DAY_657.PlasmacDNA.P4H8 -------------------- -------------------- -------------------- -------------------- --------------------

                       SHIV1157 VGIGAVFLGFLGAAGSTMGA ASITLTVQARQLLSGIVQQQ DNLLRAIEAQQHMLQLTVWG IKQLQARVLAIERYLQDQQL LGIWGCSGKLICTTAVPWND  600
 A19095.DAY_552.PlasmacDNA.P4B5 -------------------- -------------------- -------------------- -------------------- --------------------
 A19095.DAY_552.PlasmacDNA.P4B6 -------------------- -------------------- -------------------- -------------------- --------------------
 A19095.DAY_552.PlasmacDNA.P4B7 -------------------- -------------------- -------------------- -------------------- --------------------
```

```
 A19095.DAY_552.PlasmacDNA.P4B8 -------------------- -------------------- -------------------- -------------------- --------------------
 A19095.DAY_552.PlasmacDNA.P4B9 -------------------- -------------------- -------------------- -------------------- --------------------
A19095.DAY_552.PlasmacDNA.P4B10 -------------------- -------------------- -------------------- -------------------- --------------------
A19095.DAY_552.PlasmacDNA.P4B12 -------------------- -------------------- -------------------- -------------------- --------------------
A19095.DAY_552.PlasmacDNA.P4B13 -------------------- -------------------- -------------------- -------------------- --------------------
A19095.DAY_552.PlasmacDNA.P4B14 -------------------- -------------------- -------------------- -------------------- --------------------
A19095.DAY_552.PlasmacDNA.P4B16 -------------------- -------------------- -------------------- -------------------- --------------------
A19095.DAY_552.PlasmacDNA.P4B18 -------------------- -------------------- -------------------- -------------------- --------------------
A19095.DAY_552.PlasmacDNA.P4B19 -------------------- -------------------- -------------------- -------------------- --------------------
A19095.DAY_552.PlasmacDNA.P4B20 -------------------- -------------------- -------------------- -------------------- --------------------
A19095.DAY_552.PlasmacDNA.P4B21 -------------------- -------------------- -------------------- -------------------- --------------------
A19095.DAY_552.PlasmacDNA.P4B22 -------------------- -------------------- -------------------- -------------------- --------------------
A19095.DAY_552.PlasmacDNA.P4B23 -------------------- -------------------- -------------------- -------------------- --------------------
A19095.DAY_552.PlasmacDNA.P4B24 -------------------- -------------------- -------------------- -------------------- --------------------
 A19095.DAY_552.PlasmacDNA.P4C1 -------------------- -------------------- -------------------- -------------------- --------------------
 A19095.DAY_552.PlasmacDNA.P4C2 -------------------- -------------------- -------------------- -------------------- --------------------
 A19095.DAY_552.PlasmacDNA.P4C3 -------------------- -------------------- -------------------- -------------------- --------------------
 A19095.DAY_552.PlasmacDNA.P4C4 -------------------- -------------------- -------------------- -------------------- --------------------
 A19095.DAY_552.PlasmacDNA.P4C5 -------------------- -------------------- -------------------- -------------------- --------------------
 A19095.DAY_552.PlasmacDNA.P4C6 -------------------- -------------------- -------------------- -------------------- --------------------
 A19095.DAY_552.PlasmacDNA.P4C9 -------------------- -------------------- -------------------- -------------------- --------------------
A19095.DAY_552.PlasmacDNA.P4C10 -------------------- -------------------- -------------------- -------------------- --------------------
A19095.DAY_552.PlasmacDNA.P4C11 -------------------- -------------------- -------------------- -------------------- --------------------
A19095.DAY_552.PlasmacDNA.P4C12 -------------------- -------------------- -------------------- -------------------- --------------------
A19095.DAY_657.PlasmacDNA.P3A13 -------------------- -------------------- -------------------- -------------------- --------------------
A19095.DAY_657.PlasmacDNA.P3B23 -------------------- -------------------- -------------------- -------------------- --------------------
 A19095.DAY_657.PlasmacDNA.P4C9 -------------------- -------------------- -------------------- -------------------- --------------------
 A19095.DAY_657.PlasmacDNA.P4D6 -------------------- -------------------- -------------------- -------------------- --------------------
 A19095.DAY_657.PlasmacDNA.P4D7 -------------------- -------------------- -------------------- -------------------- --------------------
A19095.DAY_657.PlasmacDNA.P4D24 -------------------- -------------------- -------------------- -------------------- --------------------
 A19095.DAY_657.PlasmacDNA.P4E7 -------------------- -------------------- -------------------- -------------------- --------------------
A19095.DAY_657.PlasmacDNA.P4E15 -------------------- -------------------- -------------------- -------------------- --------------------
 A19095.DAY_657.PlasmacDNA.P4F6 -------------------- -------------------- -------------------- -------------------- --------------------
 A19095.DAY_657.PlasmacDNA.P4H2 -------------------- -------------------- -------------------- -------------------- --------------------
 A19095.DAY_657.PlasmacDNA.P4H7 -------------------- -------------------- -------------------- -------------------- --------------------
 A19095.DAY_657.PlasmacDNA.P4H8 -------------------- -------------------- -------------------- -------------------- --------------------

                       SHIV1157 SWSNKSQTDIWENMTWMQWD REISRHTDTIYRLLEDSQNQ QEKNEKDLLALDSWKNLWNW FSITRWLWYIKIFIMIVGGL IGLRIIFAVLSIVNRVRQGY  700
 A19095.DAY_552.PlasmacDNA.P4B5 -------------------- -------N------------ -------------------- -------------------- --------------------
 A19095.DAY_552.PlasmacDNA.P4B6 -------------------- -------N------------ -------------------- -------------------- --------------------
 A19095.DAY_552.PlasmacDNA.P4B7 -------------------- -------N------------ -------------------- -------------------- --------------------
 A19095.DAY_552.PlasmacDNA.P4B8 -------------------- -------N------------ -------------------- -------------------- --------------------
 A19095.DAY_552.PlasmacDNA.P4B9 -------------------- -------N------------ -------------------- -------------------- --------------------
A19095.DAY_552.PlasmacDNA.P4B10 -------------------- -------N------------ -------------------- -------------------- M-------------------
A19095.DAY_552.PlasmacDNA.P4B12 -------------------- -------N------------ -------------------- -------------------- --------------------
A19095.DAY_552.PlasmacDNA.P4B13 -------------------- -------N------------ -------------------- -------------------- --------------------
A19095.DAY_552.PlasmacDNA.P4B14 -------------------- -------N------------ -------------------- -------------------- --------------------
A19095.DAY_552.PlasmacDNA.P4B16 -------------------- -------N------------ -------------------- -------------------- --------------------
A19095.DAY_552.PlasmacDNA.P4B18 -------------------- -------N------------ -------------------- -------------------- --------------------
A19095.DAY_552.PlasmacDNA.P4B19 -------------------- -------N------------ -------------------- -------------------- --------------------
A19095.DAY_552.PlasmacDNA.P4B20 -------------------- -------N------------ -------------------- -------------------- --------------------
A19095.DAY_552.PlasmacDNA.P4B21 -------------------- -------N------------ -------------------- -------------------- --------------------
A19095.DAY_552.PlasmacDNA.P4B22 -------------------- -------N------------ -------------------- -------------------- --------------------
A19095.DAY_552.PlasmacDNA.P4B23 -------------------- -------N------------ -------------------- -------------------- --------------------
A19095.DAY_552.PlasmacDNA.P4B24 -------------------- -------N------------ -------------------- -------------------- --------------------
 A19095.DAY_552.PlasmacDNA.P4C1 -------------------- -------N------------ -------------------- -------------------- --------------------
 A19095.DAY_552.PlasmacDNA.P4C2 -------------------- -------N------------ -------------------- -------------------- --------------------
 A19095.DAY_552.PlasmacDNA.P4C3 -------------------- -------N------------ -------------------- -------------------- --------------------
 A19095.DAY_552.PlasmacDNA.P4C4 -------------------- -------N------------ -------------------- -------------------- --------------------
 A19095.DAY_552.PlasmacDNA.P4C5 -------------------- -------N------------ -------------------- -------------------- --------------------
 A19095.DAY_552.PlasmacDNA.P4C6 -------------------- -------N------------ -------------------- -------------------- --------------------
 A19095.DAY_552.PlasmacDNA.P4C9 -------------------- -------N------------ -------------------- -------------------- --------------------
A19095.DAY_552.PlasmacDNA.P4C10 -------------------- -------N------------ -------------------- -------------------- --------------------
A19095.DAY_552.PlasmacDNA.P4C11 -------------------- -------N------------ -------------------- -------------------- --------------------
A19095.DAY_552.PlasmacDNA.P4C12 -------------------- -------N------------ -------------------- -------------------- --------------------
A19095.DAY_657.PlasmacDNA.P3A13 -------------------- -------N------------ -------------------- -------------------- --------------------
A19095.DAY_657.PlasmacDNA.P3B23 -------------------- -------N------------ -------------------- -------------------- --------------------
 A19095.DAY_657.PlasmacDNA.P4C9 -------------------- -------N------------ -------------------- -------------------- --------------------
 A19095.DAY_657.PlasmacDNA.P4D6 -------------------- -------N------------ -------------------- -------------------- --------------------
 A19095.DAY_657.PlasmacDNA.P4D7 -------------------- -------N------------ -------------------- -------------------- --------------------
A19095.DAY_657.PlasmacDNA.P4D24 -------------------- -------------------- -------------------- -------------------- --------------------
 A19095.DAY_657.PlasmacDNA.P4E7 -------------------- -------N------------ -------------------- -------------------- --------------------
A19095.DAY_657.PlasmacDNA.P4E15 -------------------- -------N------------ -------------------- -------------------- --------------------
 A19095.DAY_657.PlasmacDNA.P4F6 -------------------- -------N------------ -------------------- -------------------- --------------------
 A19095.DAY_657.PlasmacDNA.P4H2 -------------------- -------N------------ -------------------- -------------------- --------------------
 A19095.DAY_657.PlasmacDNA.P4H7 -------------------- -------N------------ -------------------- -------------------- --------------------
 A19095.DAY_657.PlasmacDNA.P4H8 -------------------- -------------------- -------------------- -------------------- --------------------

                       SHIV1157 SPLSFQTHLPLPRGADRPEG IEEEGGERDRDRSIRLVTGS LALIWDDLRSLCLFSYHRLR DLLLIVTRTVELLGRRGWEA LKYWWNLLLYWSQELKNSAV  800
 A19095.DAY_552.PlasmacDNA.P4B5 -------------------- -------------V------ -------------------- -------------------- --------------------
 A19095.DAY_552.PlasmacDNA.P4B6 -------------------- -------------V------ -------------------- -------------------- --------------------
 A19095.DAY_552.PlasmacDNA.P4B7 -------------------- -------------V------ -------------------- -------------------- --------------------
 A19095.DAY_552.PlasmacDNA.P4B8 -------------------- -------------V------ -------------------- -------------------- --------------------
 A19095.DAY_552.PlasmacDNA.P4B9 -------------------- -------------V------ -------------------- -------------------- --------------------
A19095.DAY_552.PlasmacDNA.P4B10 -------------------- -------------V------ -------------------- -------------------- --------------------
A19095.DAY_552.PlasmacDNA.P4B12 -------------------- -------------V------ -------------------- -------------------- --------------------
A19095.DAY_552.PlasmacDNA.P4B13 -------------------- -------------V------ -------------------- -------------------- --------------------
A19095.DAY_552.PlasmacDNA.P4B14 -------------------- -------------V------ -------------------- -------------------- --------------------
A19095.DAY_552.PlasmacDNA.P4B16 -------------------- -------------V------ -------------------- -------------------- --------------------
A19095.DAY_552.PlasmacDNA.P4B18 -------------------- -------------V------ -------------------- -------------------- --------------------
A19095.DAY_552.PlasmacDNA.P4B19 -------------------- -------------V------ -------------------- -------------------- --------------------
A19095.DAY_552.PlasmacDNA.P4B20 -------------------- -------------V------ -------------------- -------------------- --------------------
A19095.DAY_552.PlasmacDNA.P4B21 -------------------- -------------V------ -------------------- -------------------- --------------------
A19095.DAY_552.PlasmacDNA.P4B22 -------------------E -------------V------ -------------------- -------------------- --------------------
A19095.DAY_552.PlasmacDNA.P4B23 -------------------- -------------V------ -------------------- -------------------- --------------------
A19095.DAY_552.PlasmacDNA.P4B24 -------------------- -------------V------ -------------------- -------------------- --------------------
 A19095.DAY_552.PlasmacDNA.P4C1 -------------------- -------------V------ -------------------- -------------------- --------------------
 A19095.DAY_552.PlasmacDNA.P4C2 -------------------- -------------V------ -------------------- -------------------- --------------------
 A19095.DAY_552.PlasmacDNA.P4C3 -------------------- -------------V------ -------------------- -------------------- --------------------
 A19095.DAY_552.PlasmacDNA.P4C4 -------------------- -------------V------ -------------------- -------------------- --------------------
 A19095.DAY_552.PlasmacDNA.P4C5 -------------------- -------------V------ -------------------- -------------------- --------------------
 A19095.DAY_552.PlasmacDNA.P4C6 -------------------- -------------V------ -------------------- -------------------- --------------------
 A19095.DAY_552.PlasmacDNA.P4C9 -------------------- -------------V------ -------------------- -------------------- --------------------
A19095.DAY_552.PlasmacDNA.P4C10 -------------------- -------------V------ -------------------- -------------------- --------------------
A19095.DAY_552.PlasmacDNA.P4C11 -------------------- -------------V------ -------------------- -------------------- --------------------
```

```
A19095.DAY_552.PlasmacDNA.P4C12 -------------------- -------------V------ -------------------- -------------------- --------------------
A19095.DAY_657.PlasmacDNA.P3A13 -------------------- -------------V------ -------------------- -------------------- --------------------
A19095.DAY_657.PlasmacDNA.P3B23 -------------------- -------------V------ -------------------- -------------------- --------------------
 A19095.DAY_657.PlasmacDNA.P4C9 -------------------- -------------V------ -------------------- -------------------- --------------------
 A19095.DAY_657.PlasmacDNA.P4D6 -------------------- -------------V------ -------------------- -------------------- --------------------
 A19095.DAY_657.PlasmacDNA.P4D7 -------------------- -------------V------ -------------------- -------------------- --------------------
A19095.DAY_657.PlasmacDNA.P4D24 -------------------- -------------V------ -------------------- -------------------- --------------------
 A19095.DAY_657.PlasmacDNA.P4E7 -------------------- -------------V------ -------------------- -------------------- --------------------
A19095.DAY_657.PlasmacDNA.P4E15 -------------------- -------------V------ -------------------- -----I-------------- --------------------
 A19095.DAY_657.PlasmacDNA.P4F6 -------------------- -------------V------ -------------------- ------A------------- -R------------------
 A19095.DAY_657.PlasmacDNA.P4H2 -------------------- -------------V------ -------------------- -------------------- --------------------
 A19095.DAY_657.PlasmacDNA.P4H7 -------------------E -------------V------ -------------------- -------------------- --------------------
 A19095.DAY_657.PlasmacDNA.P4H8 -------------------- -------------V------ -------------------- -------------------- --------------------

                       SHIV1157 SLLNATAIAVRQYGWSYFHE AVQAVWRSATETLAGAWGDL WEILRRGGRWILAIPRRIRQ GLELTLL*  868
 A19095.DAY_552.PlasmacDNA.P4B5 -------------------- -------------------- -------------------- --------
 A19095.DAY_552.PlasmacDNA.P4B6 -------------------- -------------------- -------------------- --------
 A19095.DAY_552.PlasmacDNA.P4B7 -------------------- -------------------- -------------------- --------
 A19095.DAY_552.PlasmacDNA.P4B8 -------------------- -------------------- -------------------- --------
 A19095.DAY_552.PlasmacDNA.P4B9 -------------------- -------------------- -------------------- --------
A19095.DAY_552.PlasmacDNA.P4B10 -------------------- -------------------- -------------------- --------
A19095.DAY_552.PlasmacDNA.P4B12 -------------------- -------------------- -------------------- --------
A19095.DAY_552.PlasmacDNA.P4B13 -------------------- -------------------- -------------------- --------
A19095.DAY_552.PlasmacDNA.P4B14 -------------------- -------------------- -------------------- --------
A19095.DAY_552.PlasmacDNA.P4B16 -------------------- -------------------- -------------------- --------
A19095.DAY_552.PlasmacDNA.P4B18 -------------------- -------------------- -------------------- --------
A19095.DAY_552.PlasmacDNA.P4B19 -------------------- -------------------- -------------------- --------
A19095.DAY_552.PlasmacDNA.P4B20 -------------------- -------------------- -------------------- --------
A19095.DAY_552.PlasmacDNA.P4B21 -------------------- -------------------- -------------------- --------
A19095.DAY_552.PlasmacDNA.P4B22 -------------------- -------------------- -------------------- --------
A19095.DAY_552.PlasmacDNA.P4B23 -------------------- -------------------- -------------------- --------
A19095.DAY_552.PlasmacDNA.P4B24 -------------------- -------------------- -------------------- --------
 A19095.DAY_552.PlasmacDNA.P4C1 -------------------- -------------------- -------------------- --------
 A19095.DAY_552.PlasmacDNA.P4C2 -------------------- -------------------- -------------------- --------
 A19095.DAY_552.PlasmacDNA.P4C3 -------------------- -------------------- -------------------- --------
 A19095.DAY_552.PlasmacDNA.P4C4 -------------------- -------------------- -------------------- --------
 A19095.DAY_552.PlasmacDNA.P4C5 -------------------- -------------------- -------------------- --------
 A19095.DAY_552.PlasmacDNA.P4C6 -------------------- -------------------- -------------------- --------
 A19095.DAY_552.PlasmacDNA.P4C9 -------------------- -------------------- -------------------- --------
A19095.DAY_552.PlasmacDNA.P4C10 -------------------- -------------------- -------------------- --------
A19095.DAY_552.PlasmacDNA.P4C11 -------------------- -------------------- -------------------- --------
A19095.DAY_552.PlasmacDNA.P4C12 -------------------- -------------------- -------------------- --------
A19095.DAY_657.PlasmacDNA.P3A13 -------------------- -------------------- --T----------------- --------
A19095.DAY_657.PlasmacDNA.P3B23 -------------------- -------------------- --T----------------- --------
 A19095.DAY_657.PlasmacDNA.P4C9 -------------------- -------------------- --T----------------- --------
 A19095.DAY_657.PlasmacDNA.P4D6 -------------------- -------------------- --T----------------- --------
 A19095.DAY_657.PlasmacDNA.P4D7 -------------------- -------------------- --T----------------- --------
A19095.DAY_657.PlasmacDNA.P4D24 -------------------- -------------------- --T----------------- --------
 A19095.DAY_657.PlasmacDNA.P4E7 -------------------- -------------------- --T----------------- --------
A19095.DAY_657.PlasmacDNA.P4E15 -------------------- -------------------- -------------------- --------
 A19095.DAY_657.PlasmacDNA.P4F6 -------------------- -------------------- --T----------------- --------
 A19095.DAY_657.PlasmacDNA.P4H2 -------------------- -------------------- --T----------------- --------
 A19095.DAY_657.PlasmacDNA.P4H7 -------------------- -------------------- --T----------------- --------
 A19095.DAY_657.PlasmacDNA.P4H8 -------------------- -------------------- --T----------------- --------
```

A19097 eCD4-Ig+Ciapavir

```
                       SHIV1157 MRVKEKYQHLWRWGWRWGIM LLGMLMICSASEKLWVTVYY GVPVWKEAKTTLFCASNAKA YEKEVHNIWATHACVPTDPN PQEIVLGNVTENFNMWKNDM  100
 A19097.DAY_574.PlasmacDNA.P4A3 -------------------- -------------------- -------------------- -------------------- --------------------
 A19097.DAY_574.PlasmacDNA.P4A4 -------------------- -------------------- ----------------D--- -------------------- --------------------
 A19097.DAY_574.PlasmacDNA.P4A7 -------------------- -------------------- ------------------R- -------------------- --------------------
 A19097.DAY_574.PlasmacDNA.P4A8 -------------------- -------------------- ------------------R- -------------------- --------------------
 A19097.DAY_574.PlasmacDNA.P4A9 -------------------- -------------------- ----------------D--- -------------------- --------------------
A19097.DAY_574.PlasmacDNA.P4A10 -------------------- -------------------- -------------------- -------------------- --------------------
A19097.DAY_574.PlasmacDNA.P4A11 -------------------- -------------------- ----------------D--- -------------------- --------------------
A19097.DAY_574.PlasmacDNA.P4A13 -------------------- ---I---------------- -------------------- -------------------- --------------------
A19097.DAY_574.PlasmacDNA.P4A14 -------------------- -------------------- -------------------- -------------------- --------------------
A19097.DAY_574.PlasmacDNA.P4A15 -------------------- -------------------- -------------------- -------------------- --------------------
A19097.DAY_574.PlasmacDNA.P4A16 -------------------- ----M--------------- ----------------D--- -------------------- --------------------
A19097.DAY_574.PlasmacDNA.P4A17 -------------------- -------------------- ------------------R- -------------------- --------------------
A19097.DAY_574.PlasmacDNA.P4A18 -------------------- -------------------- -I--------------D--- -------------------- --------------------
A19097.DAY_574.PlasmacDNA.P4A20 -------------------- ----M--------------- ----------------D--- -------------------- --------------------
A19097.DAY_574.PlasmacDNA.P4A21 -------------------- -------------------- ----------------D--- -------------------- --------------------
A19097.DAY_574.PlasmacDNA.P4A22 ---E---------------- -------------------- ----------------D--- -------------------- --------------------
A19097.DAY_574.PlasmacDNA.P4A23 -------------------- -------------------- ----------------D--- -------------------- --------------------
 A19097.DAY_574.PlasmacDNA.P4B1 -----------------C-- -------------------- ------------------R- -------------------- --------------------
 A19097.DAY_574.PlasmacDNA.P4B2 -------------------- -------------------- ----------------S--- -------------------- --------------------
 A19097.DAY_574.PlasmacDNA.P4B3 -------------------- -------------------- ----------------D--- -------------------- --------------------
 A19097.DAY_574.PlasmacDNA.P4B4 -------------------- -------------------- -------------------- -------------------- --------------------

                       SHIV1157 VDQMHEDIISLWDQSLKPCV KLTSLCVTLKCSNFTGKSNV TYKGDMEVKNCSFNVTTEIR DKKQKVYALFYRLDITPLDD NSSEYILINCNSSTITQACP  200
 A19097.DAY_574.PlasmacDNA.P4A3 -------------------- -------------------- -------------------- -------------------- --------------------
 A19097.DAY_574.PlasmacDNA.P4A4 -------------------- -------------------- -------------------- -------------------- --------------------
 A19097.DAY_574.PlasmacDNA.P4A7 -------------------- -------------------- -------------------- -------------------- --------------------
 A19097.DAY_574.PlasmacDNA.P4A8 -------------------- -------------------- -------------------- -------------------- --------------------
 A19097.DAY_574.PlasmacDNA.P4A9 -------------------- -------------------- -------------------- -------------------- --------------------
A19097.DAY_574.PlasmacDNA.P4A10 -------------------- -------------------- -------------------- -------------------- --------------------
A19097.DAY_574.PlasmacDNA.P4A11 -------------------- -------------------- -------------------- -------------------- --------------------
A19097.DAY_574.PlasmacDNA.P4A13 -------------------- -------------------- -------------------- -------------------- --------------------
A19097.DAY_574.PlasmacDNA.P4A14 -------------------- -------------------- -------------------- -------------------- --------------------
A19097.DAY_574.PlasmacDNA.P4A15 -------------------- -------------------- -------------------- -------------------- --------------------
A19097.DAY_574.PlasmacDNA.P4A16 -------------------- -------------------- -------------------- -------------------- --------------------
A19097.DAY_574.PlasmacDNA.P4A17 -------------------- -------------------- -------------------- -------------------- --------------------
A19097.DAY_574.PlasmacDNA.P4A18 -------------------- -------------------- -------------------- -------------------- --------------------
A19097.DAY_574.PlasmacDNA.P4A20 -------------------- -------------------- -------------------- -------------------- --------------------
A19097.DAY_574.PlasmacDNA.P4A21 -------------------- -------------------- -------------------- -------------------- --------------------
A19097.DAY_574.PlasmacDNA.P4A22 -------------------- -------------------- -------------------- -------------------- --------------------
A19097.DAY_574.PlasmacDNA.P4A23 -------------------- -------------------- -------------------- -------------------- --------------------
 A19097.DAY_574.PlasmacDNA.P4B1 -------------------- -------------------- -------------------- -------------------- --------------------
 A19097.DAY_574.PlasmacDNA.P4B2 -------------------- -------------------- -------------------- -------------------- --------------------
 A19097.DAY_574.PlasmacDNA.P4B3 -------------------- -------------------- -------------------- -------------------- --------------------
 A19097.DAY_574.PlasmacDNA.P4B4 -------------------- -------------------- -------------------- -------------------- --------------------

                       SHIV1157 KVNFDPIPIHYCAPAGYAIL KCNNKTFNGTGPCHNVSTVQ CTHGIKPVVSTQLLLNGSLA EGEIIIRSENLTDNVKTIIV HFNESVEITCTRPNNNTRKS  300
 A19097.DAY_574.PlasmacDNA.P4A3 -------------------- -------------------- -------------------- -------------------- --------------------
```

```
 A19097.DAY_574.PlasmacDNA.P4A4 -------------------- -------------------- -------------------- -------------------- --------------------
 A19097.DAY_574.PlasmacDNA.P4A7 -------------------- R------------------- -------------------- -------------------- --------------------
 A19097.DAY_574.PlasmacDNA.P4A8 -------------------- -------------------- -------------------- -------------------- --------------------
 A19097.DAY_574.PlasmacDNA.P4A9 -------------------- -------------------- -------------------- -------------------- --------------------
A19097.DAY_574.PlasmacDNA.P4A10 -------------------- -------------------- -------------------- -------------------- --------------------
A19097.DAY_574.PlasmacDNA.P4A11 -------------------- -------------------- -------------------- -------------------- --------------------
A19097.DAY_574.PlasmacDNA.P4A13 -------------------- -------------------- -------------------- K------------------- --------------------
A19097.DAY_574.PlasmacDNA.P4A14 -------------------- -------------------- -------------------- -------------------- --------------------
A19097.DAY_574.PlasmacDNA.P4A15 -------------------- -------------------- -------------------- -------------------- --------------------
A19097.DAY_574.PlasmacDNA.P4A16 -------------------- -------------------- -------------------- -------------------- --------------------
A19097.DAY_574.PlasmacDNA.P4A17 -------------------- -------------------- -------------------- -------------------- --------------------
A19097.DAY_574.PlasmacDNA.P4A18 -------------------- -------------------- -------------------- -------------------- --------------------
A19097.DAY_574.PlasmacDNA.P4A20 -------------------- -------------------- -------------------- -------------------- --------------------
A19097.DAY_574.PlasmacDNA.P4A21 -------------------- -------------------- -------------------- -------------------- --------------------
A19097.DAY_574.PlasmacDNA.P4A22 -------------------- -------------------- -------------------- -------------------- --------------------
A19097.DAY_574.PlasmacDNA.P4A23 -------------------- -------------------- -------------------- -------------------- --------------------
 A19097.DAY_574.PlasmacDNA.P4B1 -------------------- -------------------- -------------------- -------------------- --------------------
 A19097.DAY_574.PlasmacDNA.P4B2 -------------------- -------------------- -------------------- -------------------- --------------------
 A19097.DAY_574.PlasmacDNA.P4B3 -------------------- -------------------- -------------------- -------------------- --------------------
 A19097.DAY_574.PlasmacDNA.P4B4 -------------------- -------------------- -------------------- -------------------- --------------------

                       SHIV1157 ISIGPGQAIYATGDIIGDIR QAHCNISKENWNKTLQWVRG KLKEHFPNKTIVFKPSSGGD LEITTHSFNCRGEFFYCNTS KLFNSTDNSTHMGTENNTII  400
 A19097.DAY_574.PlasmacDNA.P4A3 -------------------- -------------------- -------------------- -------------------- --------------------
 A19097.DAY_574.PlasmacDNA.P4A4 -------------------- -------------------- -------------------- -------------------- --------------------
 A19097.DAY_574.PlasmacDNA.P4A7 -------------------- -------------------- -------------------- -------------------- --------------------
 A19097.DAY_574.PlasmacDNA.P4A8 -------------------- -------------------- -------------------- -------------------- --------------------
 A19097.DAY_574.PlasmacDNA.P4A9 -------------------- -------------------- -------------------- -------------------- --------------------
A19097.DAY_574.PlasmacDNA.P4A10 -------------------- -------------------- -------------------- -------------------- --------------------
A19097.DAY_574.PlasmacDNA.P4A11 -------------------- -------------------- -------------------- -------------------- --------------------
A19097.DAY_574.PlasmacDNA.P4A13 -------------------- -------------------- -------------------- -------------------- --------------------
A19097.DAY_574.PlasmacDNA.P4A14 -------------------- -------------------- -------------------- -------------------- --------------------
A19097.DAY_574.PlasmacDNA.P4A15 -------------------- -------------------- -------------------- -------------------- --------------------
A19097.DAY_574.PlasmacDNA.P4A16 -------------------- ----------------G--- -------------------- -------------------- --------------------
A19097.DAY_574.PlasmacDNA.P4A17 -------------------- -------------------- -------------------- -------------------- --------------------
A19097.DAY_574.PlasmacDNA.P4A18 -------------------- -------------------- -------------------- -------------------- --------------------
A19097.DAY_574.PlasmacDNA.P4A20 -------------------- -------------------- -------------------- -------------------- --------------------
A19097.DAY_574.PlasmacDNA.P4A21 -------------------- -------------------- -------------------- -------------------- --------------------
A19097.DAY_574.PlasmacDNA.P4A22 -------------------- -------------------- -------------------- -------------------- --------------------
A19097.DAY_574.PlasmacDNA.P4A23 -------------------- -------------------- -------------------- -------------------- --------------------
 A19097.DAY_574.PlasmacDNA.P4B1 -------------------- -------------------- -------------------- -------------------- --------------------
 A19097.DAY_574.PlasmacDNA.P4B2 -------------------- -------------------- -------------------- -------------------- --------------------
 A19097.DAY_574.PlasmacDNA.P4B3 -------------------- -------------------- -------------------- -------------------- --------------------
 A19097.DAY_574.PlasmacDNA.P4B4 -------------------- -------------------- -------------------- -------------------- --------------------

                       SHIV1157 TIPCRIKQIINMWQEVGRAM YAPPIEGNITCKSNITGLLL VRDGGWDNSTNDTETFRPGG GDMRDNWRSELYKYKVVEVK PLGIAPTKAKRRVVEREKRA  500
 A19097.DAY_574.PlasmacDNA.P4A3 ----------------R--- -------------------- -------------------- -------------------- --------------------
 A19097.DAY_574.PlasmacDNA.P4A4 -------------------- -------------------- -------------------- -------------------- --------------------
 A19097.DAY_574.PlasmacDNA.P4A7 -------------------- -------------------- -------------------- -------------------- --------------------
 A19097.DAY_574.PlasmacDNA.P4A8 -------------------- -------------------- -------------------- -------------------- --------------------
 A19097.DAY_574.PlasmacDNA.P4A9 -------------------- -------------------- -------------------- -------------------- --------------------
A19097.DAY_574.PlasmacDNA.P4A10 ----------------R--- -------------------- -------------------- -------------------- --------------------
A19097.DAY_574.PlasmacDNA.P4A11 -------------------- -------------------- -------------------- -------------------- --------------------
A19097.DAY_574.PlasmacDNA.P4A13 -------------------- -------------------- -------------------- -------------------- --------------------
A19097.DAY_574.PlasmacDNA.P4A14 ----------------R--- -------------------- -------------------- -------------------- --------------------
A19097.DAY_574.PlasmacDNA.P4A15 -------------------- -------------------- -------------------- -N------------------ --------------------
A19097.DAY_574.PlasmacDNA.P4A16 -------------------- -------------------- -------------------- -------------------- --------------------
A19097.DAY_574.PlasmacDNA.P4A17 -------------------- -------------------- -------------------- -------------------- --------------------
A19097.DAY_574.PlasmacDNA.P4A18 -------------------- -------------------- -------------------- -------------------- --------------------
A19097.DAY_574.PlasmacDNA.P4A20 -------------------- -------------------- -------------------- -------------------- --------------------
A19097.DAY_574.PlasmacDNA.P4A21 -------------------- -------------------- -------------------- -------------------- --------------------
A19097.DAY_574.PlasmacDNA.P4A22 -------------------- -------------------- -------------------- -------------------- --------------------
A19097.DAY_574.PlasmacDNA.P4A23 -------------------- -------------------- -------------------- -------------------- --------------------
 A19097.DAY_574.PlasmacDNA.P4B1 -------------------- -------------------- -------------------- -------------------- --------------------
 A19097.DAY_574.PlasmacDNA.P4B2 -------------------- -------------------- -------------------- -------------------- --------------G-----
 A19097.DAY_574.PlasmacDNA.P4B3 -------------------- -------------------- -------------------- -------------------- --------------------
 A19097.DAY_574.PlasmacDNA.P4B4 ----------------R--- -------------------- -------------------- -------------------- --------------------

                       SHIV1157 VGIGAVFLGFLGAAGSTMGA ASITLTVQARQLLSGIVQQQ DNLLRAIEAQQHMLQLTVWG IKQLQARVLAIERYLQDQQL LGIWGCSGKLICTTAVPWND  600
 A19097.DAY_574.PlasmacDNA.P4A3 -------------------- -------------------- -------------------- -------------------- --------------------
 A19097.DAY_574.PlasmacDNA.P4A4 -------------------- -------------------- -------------------- ----------V--------- --------------------
 A19097.DAY_574.PlasmacDNA.P4A7 -------------------- -------------------- -T------------------ ----------V--------- --------------------
 A19097.DAY_574.PlasmacDNA.P4A8 -------------------- -------------------- -------------------- ----------V--------- --------------------
 A19097.DAY_574.PlasmacDNA.P4A9 -------------------- -------------------- -------------------- ----------V--------- --------------------
A19097.DAY_574.PlasmacDNA.P4A10 -------------------- -------------------- -------------------- -------------------- --------------------
A19097.DAY_574.PlasmacDNA.P4A11 -------------------- -------------------- -------------------- ----------V--------- --------------------
A19097.DAY_574.PlasmacDNA.P4A13 -------------------- -------------------- -------------------- ----------M--------- --------------T-----
A19097.DAY_574.PlasmacDNA.P4A14 -------------------- -------------------- -------------------- -------------------- --------------------
A19097.DAY_574.PlasmacDNA.P4A15 -------------------- -------------------- -------------------- -------------------- --------------------
A19097.DAY_574.PlasmacDNA.P4A16 -------------------- -------------------- -------------------- ----------V--------- --------------------
A19097.DAY_574.PlasmacDNA.P4A17 -------------------- -------------------- -------------------- ----------V--------- --------------------
A19097.DAY_574.PlasmacDNA.P4A18 -------------------- -------------------- -------------------- ----------V--------- --------------------
A19097.DAY_574.PlasmacDNA.P4A20 -------------------- -------------------- -------------------- ----------V--------- --------------------
A19097.DAY_574.PlasmacDNA.P4A21 -------------------- -------------------- -------------------- ----------V--------- --------------------
A19097.DAY_574.PlasmacDNA.P4A22 -------------------- -------------------- -------------------- ----------V--------- --------------------
A19097.DAY_574.PlasmacDNA.P4A23 -------------------- -------------------- -------------------- ----------V--------- --------------------
 A19097.DAY_574.PlasmacDNA.P4B1 -------------------- -------------------- -------------------- ----------V--------- --------------------
 A19097.DAY_574.PlasmacDNA.P4B2 -------------------- -------------------- -------------------- -------------------- --------------------
 A19097.DAY_574.PlasmacDNA.P4B3 -------------------- -------------------- -------------------- ----------V--------- --------------------
 A19097.DAY_574.PlasmacDNA.P4B4 -------------------- -------------------- -------------------- -------------------- --------------------

                       SHIV1157 SWSNKSQTDIWENMTWMQWD REISRHTDTIYRLLEDSQNQ QEKNEKDLLALDSWKNLWNW FSITRWLWYIKIFIMIVGGL IGLRIIFAVLSIVNRVRQGY  700
 A19097.DAY_574.PlasmacDNA.P4A3 -------------------- -------------------- -------------------- -------------------- --------------------
 A19097.DAY_574.PlasmacDNA.P4A4 -------------------- -------------------- -------------------- -------------------- --------------------
 A19097.DAY_574.PlasmacDNA.P4A7 -------------------- -------------------- -------------------- -------------------- --------------------
 A19097.DAY_574.PlasmacDNA.P4A8 -------------------- -------------------- -------------------- -------------------- --------------------
 A19097.DAY_574.PlasmacDNA.P4A9 -------------------- -------------------- -------------------- -------------------- --------------------
A19097.DAY_574.PlasmacDNA.P4A10 -------------------- -------------------- -------------------- -------------------- --------------------
A19097.DAY_574.PlasmacDNA.P4A11 -------------------- -------------------- -------------------- -------------------- --------------------
A19097.DAY_574.PlasmacDNA.P4A13 -------------------- -------------------- -------------------- -------------------- --------------------
A19097.DAY_574.PlasmacDNA.P4A14 -------------------- -------------------- -------------------- -------------------- --------------------
A19097.DAY_574.PlasmacDNA.P4A15 -------------------- -------------------- -------------------- -------------------- --------------------
A19097.DAY_574.PlasmacDNA.P4A16 -------------------- -------------------- -------------------- -------------------- --------------------
A19097.DAY_574.PlasmacDNA.P4A17 -------------------- -------------------- -------------------- -------------------- --------------------
A19097.DAY_574.PlasmacDNA.P4A18 -------------------- -------------------- -------------------- -------------------- --------------------
A19097.DAY_574.PlasmacDNA.P4A20 -------------------- -------------------- -----------N-------- -------------------- --------------------
```

```
A19097.DAY_574.PlasmacDNA.P4A21 -------------------- -------------------- -------------------- -------------------- --------------------
A19097.DAY_574.PlasmacDNA.P4A22 -------------------- -------------------- -------------------- -------------------- --------------------
A19097.DAY_574.PlasmacDNA.P4A23 -------------------- -------------------- -------------------- -------------------- --------------------
 A19097.DAY_574.PlasmacDNA.P4B1 -------------------- -------------------- -------------------- -------------------- --------------------
 A19097.DAY_574.PlasmacDNA.P4B2 -------------------- -------------------- -------------------- -------------------- --------------------
 A19097.DAY_574.PlasmacDNA.P4B3 -------------------- -------------------- -------------------- -------------------- --------------------
 A19097.DAY_574.PlasmacDNA.P4B4 -------------------- -------------------- -------------------- -------------------- --------------------

                       SHIV1157 SPLSFQTHLPLPRGADRPEG IEEEGGERDRDRSIRLVTGS LALIWDDLRSLCLFSYHRLR DLLLIVTRTVELLGRRGWEA LKYWWNLLLYWSQELKNSAV  800
 A19097.DAY_574.PlasmacDNA.P4A3 -------------------- -------------------- -------------------- -------------------- --------------------
 A19097.DAY_574.PlasmacDNA.P4A4 -------------------- -------------------- -------------------- -------------------- --------------------
 A19097.DAY_574.PlasmacDNA.P4A7 -------------------- -------------------- -------------------- -------------------- --------------------
 A19097.DAY_574.PlasmacDNA.P4A8 -------------------- -------------------- -------------------- -------------------- --------------------
 A19097.DAY_574.PlasmacDNA.P4A9 -------------------- -------------------- -------------------- -------------------- --------------------
A19097.DAY_574.PlasmacDNA.P4A10 -------------------- -------------------- -------------------- -------------------- --------------------
A19097.DAY_574.PlasmacDNA.P4A11 -------------------- -------------------- -------------------- -------------------- --------------------
A19097.DAY_574.PlasmacDNA.P4A13 -------------------- -------------------- -------------------- -------------------- --------------------
A19097.DAY_574.PlasmacDNA.P4A14 -------------------- -------------------- -------------------- -------------------- --------------------
A19097.DAY_574.PlasmacDNA.P4A15 -------------------- -------------------- F------------------- -------------------- --------------------
A19097.DAY_574.PlasmacDNA.P4A16 -------------------- -------------------- -------------------- -------------------- --------------------
A19097.DAY_574.PlasmacDNA.P4A17 -------------------- -------------------- -------------------- -------------------- --------------------
A19097.DAY_574.PlasmacDNA.P4A18 -------------------- -------------------- -------------------- -------------------- --------------------
A19097.DAY_574.PlasmacDNA.P4A20 -------------------- -------------------- -------------------- -------------------- --------------------
A19097.DAY_574.PlasmacDNA.P4A21 -------------------- -------------------- -------------------- -------------------- --------------------
A19097.DAY_574.PlasmacDNA.P4A22 -------------------- -------------------- -------------------- -------------------- --------------------
A19097.DAY_574.PlasmacDNA.P4A23 -------------------- -------------------- -------------------- -------------------- --------------------
 A19097.DAY_574.PlasmacDNA.P4B1 -------------------- -------------------- -------------------- -------------------- --------------------
 A19097.DAY_574.PlasmacDNA.P4B2 -------------------- -------------------- -------------------- -------------------- --------------------
 A19097.DAY_574.PlasmacDNA.P4B3 -------------------- -------------------- -------------------- -------------------- --------------------
 A19097.DAY_574.PlasmacDNA.P4B4 -------------------- -------------------- -------------------- -------------------- --------------------

                       SHIV1157 SLLNATAIAVRQYGWSYFHE AVQAVWRSATETLAGAWGDL WEILRRGGRWILAIPRRIRQ GLELTLL*  868
 A19097.DAY_574.PlasmacDNA.P4A3 -------------------- -------------------- -------------------- --------
 A19097.DAY_574.PlasmacDNA.P4A4 -------------------- -------------------- -------------------- --------
 A19097.DAY_574.PlasmacDNA.P4A7 -------------------- -------------------- -------------------- --------
 A19097.DAY_574.PlasmacDNA.P4A8 -------------------- -------------------- -------------------- --------
 A19097.DAY_574.PlasmacDNA.P4A9 -------------------- -------------------- -------------------- --------
A19097.DAY_574.PlasmacDNA.P4A10 -------------------- -------------------- -------------------- --------
A19097.DAY_574.PlasmacDNA.P4A11 -------------------- -------------------- -------------------- --------
A19097.DAY_574.PlasmacDNA.P4A13 -------------------- -------------------- -------------------- --------
A19097.DAY_574.PlasmacDNA.P4A14 -------------------- -------------------- -------------------- --------
A19097.DAY_574.PlasmacDNA.P4A15 -------------------- -------------------- -------------------- --------
A19097.DAY_574.PlasmacDNA.P4A16 -------------------- -------------------- -------------------- --------
A19097.DAY_574.PlasmacDNA.P4A17 -------------------- -------------------- -------------------- --------
A19097.DAY_574.PlasmacDNA.P4A18 -------------------- -------------------- -------------------- --------
A19097.DAY_574.PlasmacDNA.P4A20 -------------------- -------------------- -------------------- --------
A19097.DAY_574.PlasmacDNA.P4A21 -------------------- -------------------- -------------------- --------
A19097.DAY_574.PlasmacDNA.P4A22 -------------------- -------------------- -------------------- --------
A19097.DAY_574.PlasmacDNA.P4A23 -------------------- -------------------- -------------------- --------
 A19097.DAY_574.PlasmacDNA.P4B1 -------------------- -------------------- -------------------- --------
 A19097.DAY_574.PlasmacDNA.P4B2 -------------------- -------------------- -------------------- --------
 A19097.DAY_574.PlasmacDNA.P4B3 -------------------- -------------------- -------------------- --------
 A19097.DAY_574.PlasmacDNA.P4B4 -------------------- -------------------- -------------------- --------
```

A19098 eCD4-Ig

```
                       SHIV1157 MRVKEKYQHLWRWGWRWGIM LLGMLMICSASEKLWVTVYY GVPVWKEAKTTLFCASNAKA YEKEVHNIWATHACVPTDPN PQEIVLGNVTENFNMWKNDM  100
A19098.DAY_657.PlasmacDNA.P3C18 -------------------- -------------------- -------------------- -------------------- --------------------
A19098.DAY_657.PlasmacDNA.P3C19 -------------------- -------------------- -------------------- -------------------- --------------------
 A19098.DAY_657.PlasmacDNA.P3D8 -------------------- -------------------- -------------------- -------------------- --------------------
A19098.DAY_657.PlasmacDNA.P3D12 -----N-------------- -------------------- -------------------- -------------------- --------------------
 A19098.DAY_657.PlasmacDNA.P4I9 -------------------- -------------------- -------------------- -------------------- --------I-----------
A19098.DAY_657.PlasmacDNA.P4I22 -------------------- -------------------- -------------------- -------------------- --------------------
 A19098.DAY_657.PlasmacDNA.P4J4 -------------------- -------------------- -------------------- -------------------- --------I-----------
A19098.DAY_657.PlasmacDNA.P4J11 -------------------- -------------------- -------------------- -------------------- --------------------
A19098.DAY_657.PlasmacDNA.P4J14 -------------------- -------------------- -------------------- -------------------- --------------------
A19098.DAY_657.PlasmacDNA.P4J17 -------------------- ------T------------- -------------------- -------------------- --------------------
 A19098.DAY_657.PlasmacDNA.P4K1 -------------------- -------------------- -------------------- -------------------- --------------------
 A19098.DAY_657.PlasmacDNA.P4K7 -------------------- -------------------- -------------------- -------------------- --------------------
 A19098.DAY_657.PlasmacDNA.P4K9 -------------------- -------------------- -------------------- -------------------- --------------------
A19098.DAY_657.PlasmacDNA.P4K11 -------------------- -------------------- -------------------- -------------------- --------------------
 A19098.DAY_657.PlasmacDNA.P4L2 -------------------- -------------------- -------------------- -------------------- --------------------
 A19098.DAY_657.PlasmacDNA.P4L9 -------------------- -------------------- -------------------- -------------------- --------------------
A19098.DAY_657.PlasmacDNA.P4L15 -------------------- -------------------- -------------------- -------------------- --------------------
A19098.DAY_657.PlasmacDNA.P4L20 -------------------- -------------------- -------------------- -------------------- --------------------
A19098.DAY_657.PlasmacDNA.P4M11 -------------------- -------------------- -------------------- -------------------- --------------------
 A19098.DAY_657.PlasmacDNA.P4N2 -------------------- -------------------- -------------------- -------------------- --------I-----------
 A19098.DAY_657.PlasmacDNA.P4O7 -------------------- -------------------- -------------------- -------------------- --------------------
A19098.DAY_657.PlasmacDNA.P4O11 -------------------- -------------------- -------------------- -------------------- --------------------
A19098.DAY_657.PlasmacDNA.P4O24 -------------------- -------------------- -------------------- -------------------- --------------------
 A19098.DAY_657.PlasmacDNA.P4P7 -------------------- -------------------- -------------------- -------------------- --------------------
A19098.DAY_657.PlasmacDNA.P4P18 -------------------- -------------------- -------------------- -------------------- --------------------

                       SHIV1157 VDQMHEDIISLWDQSLKPCV KLTSLCVTLKCSNFTGKSNV TYKGDMEVKNCSFNVTTEIR DKKQKVYALFYRLDITPLDD NSSEYILINCNSSTITQACP  200
A19098.DAY_657.PlasmacDNA.P3C18 A------------------- -------------------- -------------------- -------------------- --------------------
A19098.DAY_657.PlasmacDNA.P3C19 A------------------- -------------------- -------------------- -------------------- --------------------
 A19098.DAY_657.PlasmacDNA.P3D8 A------------------- -------------------- -------------------- -------------------- --------------------
A19098.DAY_657.PlasmacDNA.P3D12 A------------------- -------------------- -------------------- -------------------- --------------------
 A19098.DAY_657.PlasmacDNA.P4I9 -------------------- -------------------- -------------------- -------------------- --------------------
A19098.DAY_657.PlasmacDNA.P4I22 A------------------- -------------------- -------------------- -------------------- --------------------
 A19098.DAY_657.PlasmacDNA.P4J4 -------------------- -------------------- -------------------- -------------------- --------------------
A19098.DAY_657.PlasmacDNA.P4J11 A------------------- -------------------- -------------------- -------------------- --------------------
A19098.DAY_657.PlasmacDNA.P4J14 A------------------- -------------------- -------------------- -------------------- --------------------
A19098.DAY_657.PlasmacDNA.P4J17 A------------------- -------------------- -------------------- -------------------- --------------------
 A19098.DAY_657.PlasmacDNA.P4K1 A------------------- -------------------- -------------------- -------------------- --------------------
 A19098.DAY_657.PlasmacDNA.P4K7 A------------------- -------------------- -------------------- -------------------- --------------------
 A19098.DAY_657.PlasmacDNA.P4K9 A------------------- -------------------- -------------------- -------------------- --------------------
A19098.DAY_657.PlasmacDNA.P4K11 A------------------- -------------------- -------------------- -------------------- --------------------
 A19098.DAY_657.PlasmacDNA.P4L2 A------------------- -------------------- -------------------- -------------------- --------------------
 A19098.DAY_657.PlasmacDNA.P4L9 A------------------- -------------------- -------------------- -------------------- --------------------
A19098.DAY_657.PlasmacDNA.P4L15 A------------------- -------------------- -------------------- -------------------- --------------------
A19098.DAY_657.PlasmacDNA.P4L20 A------------------- -------------------- -------------------- -------------------- --------------------
A19098.DAY_657.PlasmacDNA.P4M11 A------------------- -------------------- -------------------- -------------------- --------------------
 A19098.DAY_657.PlasmacDNA.P4N2 -------------------- -------------------- -------------------- -------------------- --------------------
 A19098.DAY_657.PlasmacDNA.P4O7 A------------------- -------------------- -------------------- -------------------- --------------------
A19098.DAY_657.PlasmacDNA.P4O11 A------------------- -------------------- -------------------- -------------------- --------------------
```

```
A19098.DAY_657.PlasmacDNA.P4O24 -------------------- -------------------- -------------------- -------------------- --------------------
 A19098.DAY_657.PlasmacDNA.P4P7 -------------------- -------------------- -------------------- -------------------- --------------------
A19098.DAY_657.PlasmacDNA.P4P18 A------------------- -------------------- -------------------- -------------------- --------------------

                       SHIV1157 KVNFDPIPIHYCAPAGYAIL KCNNKTFNGTGPCHNVSTVQ CTHGIKPVVSTQLLLNGSLA EGEIIIRSENLTDNVKTIIV HFNESVEITCTRPNNNTRKS  300
A19098.DAY_657.PlasmacDNA.P3C18 -------------------- -------------------- -------------------- -------------------- --------------------
A19098.DAY_657.PlasmacDNA.P3C19 -------------------- -------------------- -------------------- -------------------- --------------------
 A19098.DAY_657.PlasmacDNA.P3D8 -------------------- -------------------- -------------------- -------------------- --------------------
A19098.DAY_657.PlasmacDNA.P3D12 -------------------- -------------------- -------------------- -------------------- --------------------
 A19098.DAY_657.PlasmacDNA.P4I9 -------------------- -------------------- -------------------- -------------------- --------------------
A19098.DAY_657.PlasmacDNA.P4I22 -------------------- -------------------- -------------------- -------------------- --------------------
 A19098.DAY_657.PlasmacDNA.P4J4 -------------------- -------------------- -------------------- -------------------A --------------------
A19098.DAY_657.PlasmacDNA.P4J11 -------------------- -------------------- -------------------- -------------------- --------------------
A19098.DAY_657.PlasmacDNA.P4J14 -------------------- -------------------- -------------------- -------------------- --------------------
A19098.DAY_657.PlasmacDNA.P4J17 -------------------- -------------------- -------------------- -------------------- --------------------
 A19098.DAY_657.PlasmacDNA.P4K1 -------------------- -------------------- -------------------- -------------------- --------------------
 A19098.DAY_657.PlasmacDNA.P4K7 -------------------- -------------------- -------------------- -------------------- --------------------
 A19098.DAY_657.PlasmacDNA.P4K9 -------------------- -------------------- -------------------- -------------------- --------------------
A19098.DAY_657.PlasmacDNA.P4K11 -------------------- -------------------- -------------------- -------------------- --------------------
 A19098.DAY_657.PlasmacDNA.P4L2 -------------------- -------------------- -------------------- -------------------- --------------------
 A19098.DAY_657.PlasmacDNA.P4L9 -------------------- -------------------- -------------------- -------------------- --------------------
A19098.DAY_657.PlasmacDNA.P4L15 -------------------- -------------------- -------------------- -------------------- --------------------
A19098.DAY_657.PlasmacDNA.P4L20 -------------------- -------------------- -------------------- -------------------- --------------------
A19098.DAY_657.PlasmacDNA.P4M11 -------------------- -------------------- -------------------- -------------------- --------------------
 A19098.DAY_657.PlasmacDNA.P4N2 -------------------- -------------------- -------------------- -------------------- --------------------
 A19098.DAY_657.PlasmacDNA.P4O7 -------------------- -------------------- -------------------- -E------------------ --------------------
A19098.DAY_657.PlasmacDNA.P4O11 -------------------- -------------------- -------------------- -------------------- --------------------
A19098.DAY_657.PlasmacDNA.P4O24 -------------------- -------------------- -------------------- -------------------- --------------------
 A19098.DAY_657.PlasmacDNA.P4P7 -------------------- -------------------- -------------------- -------------------- --------------------
A19098.DAY_657.PlasmacDNA.P4P18 -------------------- -------------------- -------------------- -------------------- --------------------

                       SHIV1157 ISIGPGQAIYATGDIIGDIR QAHCNISKENWNKTLQWVRG KLKEHFPNKTIVFKPSSGGD LEITTHSFNCRGEFFYCNTS KLFNSTDNSTHMGTENNTII  400
A19098.DAY_657.PlasmacDNA.P3C18 -------------------- -------------------- -------------------- -------------------- --------------------
A19098.DAY_657.PlasmacDNA.P3C19 -------------------- -------------------- -------------------- -------------------- --------------------
 A19098.DAY_657.PlasmacDNA.P3D8 -------------------- -------------------- -------------------- -------------------- --------------------
A19098.DAY_657.PlasmacDNA.P3D12 -------------------- -------------------- -------------------- -------------------- --------------------
 A19098.DAY_657.PlasmacDNA.P4I9 -------------------- -------------------- -------------------- -------------------- --------------------
A19098.DAY_657.PlasmacDNA.P4I22 -------------------- -------------------- -------------------- -------------------- --------------------
 A19098.DAY_657.PlasmacDNA.P4J4 -------------------- -------------------- -------------------- -------------------- --------------------
A19098.DAY_657.PlasmacDNA.P4J11 -------------------- -------------------- -------------------- -------------------- --------------------
A19098.DAY_657.PlasmacDNA.P4J14 -------------------- -------------------- ------L------------- -------------------- --------------------
A19098.DAY_657.PlasmacDNA.P4J17 -------------------- -------------------- -------------------- -------------------- --------------------
 A19098.DAY_657.PlasmacDNA.P4K1 -------------------- -------------------- -------------------- -------------------- --------------------
 A19098.DAY_657.PlasmacDNA.P4K7 -------------------- -------------------- -------------------- -------------------- --------------------
 A19098.DAY_657.PlasmacDNA.P4K9 -------------------- -------------------- -------------------- -------------------- --------------------
A19098.DAY_657.PlasmacDNA.P4K11 -------------------- -------------------- -------------------- -------------------- --------------------
 A19098.DAY_657.PlasmacDNA.P4L2 -------------------- -------------------- -------------------- -------------------- --------------------
 A19098.DAY_657.PlasmacDNA.P4L9 -------------------- -------------------- -------------------- -------------------- --------------------
A19098.DAY_657.PlasmacDNA.P4L15 -------------------- -------------------- -------------------- -------------------- --------------------
A19098.DAY_657.PlasmacDNA.P4L20 -------------------- -------------------- -------------------- -------------------- --------------------
A19098.DAY_657.PlasmacDNA.P4M11 -------------------- -------------------- -------------------- -------------------- --------------------
 A19098.DAY_657.PlasmacDNA.P4N2 -------------------- -------------------- -------------------- -------------------- --------------------
 A19098.DAY_657.PlasmacDNA.P4O7 -------------------- -------------------- -------------------- -------------------- --------------------
A19098.DAY_657.PlasmacDNA.P4O11 -------------------- -------------------- -------------------- -------------------- --------------------
A19098.DAY_657.PlasmacDNA.P4O24 -------------------- -------------------- -------------------- -------------------- --------------------
 A19098.DAY_657.PlasmacDNA.P4P7 -------------------- -------------------- -------------------- -------------------- --------------------
A19098.DAY_657.PlasmacDNA.P4P18 -------------------- -------------------- -------------------- -------------------- --------------------

                       SHIV1157 TIPCRIKQIINMWQEVGRAM YAPPIEGNITCKSNITGLLL VRDGGWDNSTNDTETFRPGG GDMRDNWRSELYKYKVVEVK PLGIAPTKAKRRVVEREKRA  500
A19098.DAY_657.PlasmacDNA.P3C18 -------------------- -------------------- -------------------- -------------------- --------------------
A19098.DAY_657.PlasmacDNA.P3C19 -------------------- -------------------- -------------------- -------------------- --------------------
 A19098.DAY_657.PlasmacDNA.P3D8 -------------------- -------------------- -------------------- -------------------- --------------------
A19098.DAY_657.PlasmacDNA.P3D12 -------------------- -------------------- -------------------- -------------------- --------------------
 A19098.DAY_657.PlasmacDNA.P4I9 -------------------- -------------------- -------------------- -------------------- --------------------
A19098.DAY_657.PlasmacDNA.P4I22 -------------------- -------------------- -------------------- -------------------- --------------------
 A19098.DAY_657.PlasmacDNA.P4J4 -------------------- -------------------- -------------------- -------------------- --------------------
A19098.DAY_657.PlasmacDNA.P4J11 -------------------- -------------------- -------------------- ---------K---------- --------------------
A19098.DAY_657.PlasmacDNA.P4J14 -------------------- -------------------- -------------------- -------------------- --------------------
A19098.DAY_657.PlasmacDNA.P4J17 -------------------- -------------------- -------------------- -------------------- --------------------
 A19098.DAY_657.PlasmacDNA.P4K1 -------------------- -------------------- -------------------- -------------------- --------------------
 A19098.DAY_657.PlasmacDNA.P4K7 -------------------- -------------------- -------------------- -------------------- --------------------
 A19098.DAY_657.PlasmacDNA.P4K9 -------------------- -------------------- -------------------- -------------------- --------------------
A19098.DAY_657.PlasmacDNA.P4K11 -------------------- -------------------- -------------------- -------------------- --------------------
 A19098.DAY_657.PlasmacDNA.P4L2 -------------------- -------------------- -------------------- -------------------- --------------------
 A19098.DAY_657.PlasmacDNA.P4L9 -------------------- -------------------- -------------------- -------------------- --------------------
A19098.DAY_657.PlasmacDNA.P4L15 -------------------- -------------------- -------------------- -------------------- --------------------
A19098.DAY_657.PlasmacDNA.P4L20 -------------------- -------------------- -------------------- -------------------- --------------------
A19098.DAY_657.PlasmacDNA.P4M11 -------------------- -------------------- -------------------- -------------------- --------------------
 A19098.DAY_657.PlasmacDNA.P4N2 -------------------- -------------------- -------------------- -------------------- --------------------
 A19098.DAY_657.PlasmacDNA.P4O7 -------------------- -------------------- -------------------- -------------------- --------------------
A19098.DAY_657.PlasmacDNA.P4O11 -------------------- -------------------- -------------------- -------------------- --------------------
A19098.DAY_657.PlasmacDNA.P4O24 -------------------- -------------------- -------------------- -------------------- --------------------
 A19098.DAY_657.PlasmacDNA.P4P7 -------------------- -------------------- -------------------- -------------------- --------------------
A19098.DAY_657.PlasmacDNA.P4P18 -------------------- -------------------- -------------------- -------------------- --------------------

                       SHIV1157 VGIGAVFLGFLGAAGSTMGA ASITLTVQARQLLSGIVQQQ DNLLRAIEAQQHMLQLTVWG IKQLQARVLAIERYLQDQQL LGIWGCSGKLICTTAVPWND  600
A19098.DAY_657.PlasmacDNA.P3C18 -------------------- -------------------- -------------------- -------------------- --------------------
A19098.DAY_657.PlasmacDNA.P3C19 -------------------- -------------------- -------------------- -------------------- --------------------
 A19098.DAY_657.PlasmacDNA.P3D8 -------------------- -------------------- -------------------- -------------------- --------------------
A19098.DAY_657.PlasmacDNA.P3D12 -------------------- -------------------- -------------------- -------------------- --------------------
 A19098.DAY_657.PlasmacDNA.P4I9 -------------------- -------------------- -------------------- -------------------- -----------R--------
A19098.DAY_657.PlasmacDNA.P4I22 -------------------- -------------------- -------------------- -------------------- --------------------
 A19098.DAY_657.PlasmacDNA.P4J4 -------------------- -------------------- -------------------- -------------------- --------------------
A19098.DAY_657.PlasmacDNA.P4J11 -------------------- -------------------- -------------------- -------------------- --------------------
A19098.DAY_657.PlasmacDNA.P4J14 -------------------- -------------------- -------------------- -------------------- --------------------
A19098.DAY_657.PlasmacDNA.P4J17 -------------------- -------------------- -------------------- -------------------- --------------------
 A19098.DAY_657.PlasmacDNA.P4K1 -------------------- -------------------- -------------------- -------------------- --------------------
 A19098.DAY_657.PlasmacDNA.P4K7 -------------------- -------------------- -------------------- -------------------- --------------------
 A19098.DAY_657.PlasmacDNA.P4K9 -------------------- -------------------- -------------------- -------------------- --------------------
A19098.DAY_657.PlasmacDNA.P4K11 -------------------- -------------------- -------------------- -------------------- --------------------
 A19098.DAY_657.PlasmacDNA.P4L2 -------------------- -------------------- -------------------- -------------------- --------------------
 A19098.DAY_657.PlasmacDNA.P4L9 -------------------- -------------------- -------------------- -------------------- --------------------
A19098.DAY_657.PlasmacDNA.P4L15 -------------------- -------------------- -------------------- -------------------- --------------------
A19098.DAY_657.PlasmacDNA.P4L20 -------------------- -------------------- -------------------- -------------------- --------------------
A19098.DAY_657.PlasmacDNA.P4M11 -------------------- -------------------- -------------------- -------------------- --------------------
```

```
A19098.DAY_657.PlasmacDNA.P4N2 -------------------- -------------------- -------------------- -------------------- --------------------
A19098.DAY_657.PlasmacDNA.P4O7 -------------------- -------------------- -------------------- -------------------- --------------------
A19098.DAY_657.PlasmacDNA.P4O11 -------------------- -------------------- -------------------- -------------------- --------------------
A19098.DAY_657.PlasmacDNA.P4O24 -------------------- -------------------- -------------------- -------------------- --------------------
A19098.DAY_657.PlasmacDNA.P4P7 -------------------- -------------------- -------------------- -------------------- --------------------
A19098.DAY_657.PlasmacDNA.P4P18 -------------------- -------------------- -------------------- -------------------- --------------------

SHIV1157 SWSNKSQTDIWENMTWMQWD REISRHTDTIYRLLEDSQNQ QEKNEKDLLALDSWKNLWNW FSITRWLWYIKIFIMIVGGL IGLRIIFAVLSIVNRVRQGY 700
A19098.DAY_657.PlasmacDNA.P3C18 -------------------- -------G------------ -------------------- -------------------- --------------------
A19098.DAY_657.PlasmacDNA.P3C19 -------------------- -------G------------ -------------------- -------------------- --------------------
A19098.DAY_657.PlasmacDNA.P3D8 -------------------- -------------------- -------------------- -------------------- --------------------
A19098.DAY_657.PlasmacDNA.P3D12 -------------------- --T----------------- -------------------- -------------------- --------------------
A19098.DAY_657.PlasmacDNA.P4I9 -------------------- -------G------------ -------------------- -------------------- --------------------
A19098.DAY_657.PlasmacDNA.P4I22 -------------------- -----Y-------------- -------------------- -------------------- --------------------
A19098.DAY_657.PlasmacDNA.P4J4 -----------D-------- -------------------- -------------------- -------------------- --------------------
A19098.DAY_657.PlasmacDNA.P4J11 -------------------- -------------------- ----K--------------- -------------------- --------------------
A19098.DAY_657.PlasmacDNA.P4J14 -------------------- -------------------- -------------------- -------------------- --------------------
A19098.DAY_657.PlasmacDNA.P4J17 -------------------- -----Y-------------- -------------------- -------------------- --------------------
A19098.DAY_657.PlasmacDNA.P4K1 -------------------- -------N------------ -------------------- -------------------- --------------------
A19098.DAY_657.PlasmacDNA.P4K7 -------------------- -----Y-------------- -------------------- -------------------- --------------------
A19098.DAY_657.PlasmacDNA.P4K9 -------------------- -------------------- -------------------- -------------------- --------------------
A19098.DAY_657.PlasmacDNA.P4K11 -------------------- -----Y-------------- -------------------- -------------------- --------------------
A19098.DAY_657.PlasmacDNA.P4L2 -------------------- -------------------- -------------------- -------------------- --------------------
A19098.DAY_657.PlasmacDNA.P4L9 -------------------- -----Y-------------- -------------------- -------------------- --------------------
A19098.DAY_657.PlasmacDNA.P4L15 -------------------- -----Y-------------- -------------------- -------------------- --------------------
A19098.DAY_657.PlasmacDNA.P4L20 -------------------- -------------------- -------------------- -----------------R-- --------------------
A19098.DAY_657.PlasmacDNA.P4M11 -------------------- -------N------------ -------------------- -------------------- --------------------
A19098.DAY_657.PlasmacDNA.P4N2 -------------------- -------G------------ -------------------- ----------------A--- --------------------
A19098.DAY_657.PlasmacDNA.P4O7 -------------------- ------I------------- -------------------- -------------------- --------------------
A19098.DAY_657.PlasmacDNA.P4O11 -------------------- -------------------- -------------------- -------------------- --------------------
A19098.DAY_657.PlasmacDNA.P4O24 -------------------- -----Y-------------- -------------------- -------------------- --------------------
A19098.DAY_657.PlasmacDNA.P4P7 -------------------- -------------------- -------------------- -------------------- --------------------
A19098.DAY_657.PlasmacDNA.P4P18 -------------------- -----Y-------------- -------------------- -------------------- --------------------

SHIV1157 SPLSFQTHLPLPRGADRPEG IEEEGGERDRDRSIRLVTGS LALIWDDLRSLCLFSYHRLR DLLLIVTRTVELLGRRGWEA LKYWWNLLLYWSQELKNSAV 800
A19098.DAY_657.PlasmacDNA.P3C18 -------------------- -------------V------ -------------------- -------------------- --------------------
A19098.DAY_657.PlasmacDNA.P3C19 -------------------- -------------V------ ---------N---------- -------------------- --------------------
A19098.DAY_657.PlasmacDNA.P3D8 -------------------- -------------V------ -------------------- -------------------- --------------------
A19098.DAY_657.PlasmacDNA.P3D12 -------------------- -------------V------ -------------------- -------------------- --------------------
A19098.DAY_657.PlasmacDNA.P4I9 -------------------- -------------V------ -------------------- -------------------- --------------------
A19098.DAY_657.PlasmacDNA.P4I22 -------------------- -------------V------ -------------------- -------------------- --------------------
A19098.DAY_657.PlasmacDNA.P4J4 -------------------- -------------V------ -------------------- -----I-------------- --------------------
A19098.DAY_657.PlasmacDNA.P4J11 -------------------- -------------V------ -------------------- -------------------- --------------------
A19098.DAY_657.PlasmacDNA.P4J14 -------------------- -------------V------ -------------------- -------------------- --------------------
A19098.DAY_657.PlasmacDNA.P4J17 -------------------- -------------V------ -------------------- -------------------- --------------------
A19098.DAY_657.PlasmacDNA.P4K1 -------------------- -------------V------ -------------------- -------------------- --------------------
A19098.DAY_657.PlasmacDNA.P4K7 -------------------- -------------V------ -------------------- -------------------- --------------------
A19098.DAY_657.PlasmacDNA.P4K9 -------------------- -------------V------ -------------------- -------------------- --------------------
A19098.DAY_657.PlasmacDNA.P4K11 -------------------- -------------V------ -------------------- -------------------- --------------------
A19098.DAY_657.PlasmacDNA.P4L2 -------------------- -------------V------ -------------------- -------------------- --------------------
A19098.DAY_657.PlasmacDNA.P4L9 -------------------- -------------V------ -------------------- -------------------- --------------------
A19098.DAY_657.PlasmacDNA.P4L15 -------------------- -------------V------ -------------------- -------------------- --------------------
A19098.DAY_657.PlasmacDNA.P4L20 -------------------- -------------V------ -------------------- -------------------- --------------------
A19098.DAY_657.PlasmacDNA.P4M11 -------------------- -------------V------ -------------------- -------------------- --------------------
A19098.DAY_657.PlasmacDNA.P4N2 -------------------- -------------V------ -------------------- -------------------- --------------------
A19098.DAY_657.PlasmacDNA.P4O7 -------------------- -------------V------ -------------------- -------------------- --------------------
A19098.DAY_657.PlasmacDNA.P4O11 -------------------- -------------V------ -------------------- -------------------- --------------------
A19098.DAY_657.PlasmacDNA.P4O24 -------------------- -------------V------ -------------------- -------------------- --------------------
A19098.DAY_657.PlasmacDNA.P4P7 -------------------- -------------V------ -------------------- -------------------- --------------------
A19098.DAY_657.PlasmacDNA.P4P18 -------------------- -------------V------ -------------------- -------------------- --------------------

SHIV1157 SLLNATAIAVRQYGWSYFHE AVQAVWRSATETLAGAWGDL WEILRRGGRWILAIPRRIRQ GLELTLL*  868
A19098.DAY_657.PlasmacDNA.P3C18 -------------------- -------------------- -------------------- --------
A19098.DAY_657.PlasmacDNA.P3C19 -------------------- -------------------- -------------------- --------
A19098.DAY_657.PlasmacDNA.P3D8 -------------------- -------------------- -------------------- --------
A19098.DAY_657.PlasmacDNA.P3D12 -------------------- -------------------- -------------------- --------
A19098.DAY_657.PlasmacDNA.P4I9 -------------------- -------------------- -------------------- --------
A19098.DAY_657.PlasmacDNA.P4I22 -------------------- -------------------- -------------------- --------
A19098.DAY_657.PlasmacDNA.P4J4 -------------------- -------------------- -------------------- --------
A19098.DAY_657.PlasmacDNA.P4J11 -------------------- -------------------- -------------------- --------
A19098.DAY_657.PlasmacDNA.P4J14 -------------------- -------------------- -------------------- --------
A19098.DAY_657.PlasmacDNA.P4J17 -------------------- -------------------- -------------------- --------
A19098.DAY_657.PlasmacDNA.P4K1 -------------------- -------------------- -------------------- --------
A19098.DAY_657.PlasmacDNA.P4K7 -------------------- -------------------- -------------------- --------
A19098.DAY_657.PlasmacDNA.P4K9 -------------------- -------------------- -------------------- --------
A19098.DAY_657.PlasmacDNA.P4K11 -------------------- -------------------- -------------------- --------
A19098.DAY_657.PlasmacDNA.P4L2 -------------------- -------------------- -------------------- --------
A19098.DAY_657.PlasmacDNA.P4L9 -------------------- -------------------- -G------------------ --------
A19098.DAY_657.PlasmacDNA.P4L15 -------------------- -------------------- -------------------- --------
A19098.DAY_657.PlasmacDNA.P4L20 -------------------- -------------------- -------------------- --------
A19098.DAY_657.PlasmacDNA.P4M11 -------------------- -------------------- -------------------- --------
A19098.DAY_657.PlasmacDNA.P4N2 -------------------- -------------------- -------------------- --------
A19098.DAY_657.PlasmacDNA.P4O7 -------------------- -------------------- -------------------- --------
A19098.DAY_657.PlasmacDNA.P4O11 -------------------- -------------------- -------------------- --------
A19098.DAY_657.PlasmacDNA.P4O24 -------------------- -------------------- -------------------- --------
A19098.DAY_657.PlasmacDNA.P4P7 ----------.--------- -------------------- -------------------- --------
A19098.DAY_657.PlasmacDNA.P4P18 -------------------- -------------------- -------------------- --------
```

A19100 eCD4-Ig+Ciapavir

```
SHIV1157 MRVKEKYQHLWRWGWRWGIM LLGMLMICSASEKLWVTVYY GVPVWKEAKTTLFCASNAKA YEKEVHNIWATHACVPTDPN PQEIVLGNVTENFNMWKNDM 100
A19100.DAY_573.PlasmacDNA.P4C13 -------------------- -------------------- -------------------- -------------------- --------------------
A19100.DAY_573.PlasmacDNA.P4C14 ------------------VV -------------------- -------------------- -------------------- --------------------
A19100.DAY_573.PlasmacDNA.P4C15 -------------------- F-------N----------- -------------------- -------------------- --------------------
A19100.DAY_573.PlasmacDNA.P4C17 -------------------- F-------N----------- -------------------- -------------------- --------------------
A19100.DAY_573.PlasmacDNA.P4C19 -------------------- -------------------- -------------------- -------------------- --------------------
A19100.DAY_573.PlasmacDNA.P4C20 -------------------- -------------------- -------------------- -------------------- --------------------
A19100.DAY_573.PlasmacDNA.P4C21 -------------------- -------------------- -------------------- -------------------- --------------------
A19100.DAY_573.PlasmacDNA.P4C22 ---------------G---- -------------------- -------------------- -------------------- --------------------
A19100.DAY_573.PlasmacDNA.P4D2 -------------------V -------------------- -------------------- -------------------- --------------------
A19100.DAY_573.PlasmacDNA.P4D3 -------------------V -------------------- -------------------- -------------------- --------------------
A19100.DAY_573.PlasmacDNA.P4D4 -------------------- -------------------- -------------------- -------------------- --------------------
A19100.DAY_650.PlasmacDNA.P4I2 -------------------- -------------------- -------------------- -------------------- --------------------
A19100.DAY_650.PlasmacDNA.P4I3 -------------------- -------------------- -------------------- -------------------- --------------------
A19100.DAY_650.PlasmacDNA.P4I20 -------------------- -------------------- -------------------- -------------------- --------------------
A19100.DAY_650.PlasmacDNA.P4J4 -------------------V -------------------- -------------------- -------------------- --------------------
```

```
A19100.DAY_650.PlasmacDNA.P4J19 -------------------- -------------------- -------------------- -------------------- --------------------
A19100.DAY_650.PlasmacDNA.P4J22 -------------------- -------------------- -------------------- -------------------- --------------------
 A19100.DAY_650.PlasmacDNA.P4K3 -------------------- -------------------- -------------------- -------------------- --------------------
A19100.DAY_650.PlasmacDNA.P4K10 -------------------- -------------------- -------------------- -------------------- --------------------
A19100.DAY_650.PlasmacDNA.P4K12 -------------------V -------------------- -------------------- -------------------- --------------------
A19100.DAY_650.PlasmacDNA.P4K17 -------------------- -------------------- -------------------- -------------------- --------------------
A19100.DAY_650.PlasmacDNA.P4K20 -------------------- -------------------- -------------------- -------------------- --------------------
 A19100.DAY_650.PlasmacDNA.P4L7 -------------------- -------------------- -------------------- -------------------- --------------------
A19100.DAY_650.PlasmacDNA.P4L11 -------------------- -------------------- -------------------- -------------------- --------------------
 A19100.DAY_650.PlasmacDNA.P4M3 -------------------- -------------------- -------------------- -------------------- --------------------
 A19100.DAY_650.PlasmacDNA.P4M9 -------------------V -------------------- -------------------- -------------------- --------------------
A19100.DAY_650.PlasmacDNA.P4M16 -------------------- -------------------- -------------------- -------------------- --------------------
 A19100.DAY_650.PlasmacDNA.P4N4 -------------------- -------------------- -------------------- -------------------- --------------------
 A19100.DAY_650.PlasmacDNA.P4N7 -------------------- -------------------- -------------------- -------------------- --------------------
 A19100.DAY_650.PlasmacDNA.P4O4 -------------------- -------------------- -------------------- -------------------- --K-----------------
 A19100.DAY_650.PlasmacDNA.P4O6 -------------------- -------------------- -------------------- -------------------- --------------------
 A19100.DAY_650.PlasmacDNA.P4O7 -------------------- -------------------- -------------------- -------------------- --------------------
A19100.DAY_650.PlasmacDNA.P4O10 -------------------- -------------------- -------------------- -------------------- --------------------
A19100.DAY_650.PlasmacDNA.P4O15 -------------------- -------------------- -------------------- -------------------- --------------------
A19100.DAY_650.PlasmacDNA.P4O23 -------------------- -------------------- -------------------- -------------------- --------------------
A19100.DAY_650.PlasmacDNA.P4P17 -------------------- -------------------- -------------------- -------------------- --------------------
A19100.DAY_650.PlasmacDNA.P4P20 -------------------V -------------------- -------------------- -------------------- ---T----------------

                       SHIV1157 VDQMHEDIISLWDQSLKPCV KLTSLCVTLKCSNFTGKSNV TYKGDMEVKNCSFNVTTEIR DKKQKVYALFYRLDITPLDD NSSEYILINCNSSTITQACP  200
A19100.DAY_573.PlasmacDNA.P4C13 -------------------- -------------------- -------------------- -------------------- --------------------
A19100.DAY_573.PlasmacDNA.P4C14 -------------------- -------------------- -------------------- -------------------- --------------------
A19100.DAY_573.PlasmacDNA.P4C15 -------------------- -------------------- -------------------- -------------------- --------------------
A19100.DAY_573.PlasmacDNA.P4C17 -------------------- -------------------- -------------------- -------------------- --------------------
A19100.DAY_573.PlasmacDNA.P4C19 -------------------- -------------------- -------------------- -------------------- --------------------
A19100.DAY_573.PlasmacDNA.P4C20 -------------------- -------------------- -------------------- ------------------N- --------------------
A19100.DAY_573.PlasmacDNA.P4C21 -------------------- -------------------- -------------------- -------------------- --------------------
A19100.DAY_573.PlasmacDNA.P4C22 -------------------- -------------------- -------------------- -------------------- --------------------
 A19100.DAY_573.PlasmacDNA.P4D2 -------------------- -------------------- -------------------- -------------------- --------------------
 A19100.DAY_573.PlasmacDNA.P4D3 -------------------- -------------------- -------------------- -------------------- --------------------
 A19100.DAY_573.PlasmacDNA.P4D4 -------------------- -------------------- -------------------- -------------------- --------------------
 A19100.DAY_650.PlasmacDNA.P4I2 -------------------- -------------------- ----E--------------- -------------------- --------------------
 A19100.DAY_650.PlasmacDNA.P4I3 -------------------- -------------------- -------------------- -------------------- --------------------
A19100.DAY_650.PlasmacDNA.P4I20 -------------------- -------------------- ----E--------------- -------------------- --------------------
 A19100.DAY_650.PlasmacDNA.P4J4 -------------------- -------------------- -------------------- -------------------- --------------------
A19100.DAY_650.PlasmacDNA.P4J19 -------------------- -------------------- -------------------- -------------------- --------------------
A19100.DAY_650.PlasmacDNA.P4J22 -------------------- -------------------- -------------------- -------------------- --------------------
 A19100.DAY_650.PlasmacDNA.P4K3 -------------------- -------------------- -------------------- -------------------- --------------------
A19100.DAY_650.PlasmacDNA.P4K10 -------------------- -------------------- -------------------- -------------------- --------------------
A19100.DAY_650.PlasmacDNA.P4K12 ------------------G- -------------------- -------------------- -------------------- --------------------
A19100.DAY_650.PlasmacDNA.P4K17 -------------------- -------------------- ---E---------------- -------------------- --------------------
A19100.DAY_650.PlasmacDNA.P4K20 -------------------- -------------------- -------------------- -------------------- --------------------
 A19100.DAY_650.PlasmacDNA.P4L7 -------------------- -------------------- -------------------- -------------------- --------------------
A19100.DAY_650.PlasmacDNA.P4L11 -------------------- -------------------- -------------------- -------------------- --------------------
 A19100.DAY_650.PlasmacDNA.P4M3 -------------------- -------------------- -------------------- -------------------- --------------------
 A19100.DAY_650.PlasmacDNA.P4M9 -------------------- -------------------- -------------------- -------------------- --------------------
A19100.DAY_650.PlasmacDNA.P4M16 -------------------- -------------------- -------------------- -------------------- --------------------
 A19100.DAY_650.PlasmacDNA.P4N4 -------------------- ----------Y--------- -------------------- -------------------- --------------------
 A19100.DAY_650.PlasmacDNA.P4N7 -------------------- -------------------- -------------------- -------------------- --------------------
 A19100.DAY_650.PlasmacDNA.P4O4 -------------------- -------------------- -------------------- -------------------- --------------------
 A19100.DAY_650.PlasmacDNA.P4O6 -------------------- -------------------- -------------------- -------------------- --------------------
 A19100.DAY_650.PlasmacDNA.P4O7 -------------------- -------------------- -------------------- -------------------- --------------------
A19100.DAY_650.PlasmacDNA.P4O10 -------------------- -------------------- -------------------- -------------------- --------------------
A19100.DAY_650.PlasmacDNA.P4O15 -------------------- ----------Y--------- -------------------- -------------------- --------------------
A19100.DAY_650.PlasmacDNA.P4O23 -------------------- -------------------- -------------------- -------------------- --------------------
A19100.DAY_650.PlasmacDNA.P4P17 -------------------- -------------------- -------------------- -------------------- --------------------
A19100.DAY_650.PlasmacDNA.P4P20 -------------------- -------------------- -------------------- -------------------- --------------------

                       SHIV1157 KVNFDPIPIHYCAPAGYAIL KCNNKTFNGTGPCHNVSTVQ CTHGIKPVVSTQLLLNGSLA EGEIIIRSENLTDNVKTIIV HFNESVEITCTRPNNNTRKS  300
A19100.DAY_573.PlasmacDNA.P4C13 -------------------- -------------------- -------------------- -------------------- --------------------
A19100.DAY_573.PlasmacDNA.P4C14 -------------------- -------------------- -------------------- -------------------- --------------------
A19100.DAY_573.PlasmacDNA.P4C15 -------------------- -------------------- -------------------- -------------------- --------------------
A19100.DAY_573.PlasmacDNA.P4C17 -------------------- -------------------- -------------------- -------------------- --------------------
A19100.DAY_573.PlasmacDNA.P4C19 -------------------- -------------------- -------------------- -------------------- --------------------
A19100.DAY_573.PlasmacDNA.P4C20 -------------------- -------------------- -------------------- -------------------- --------------------
A19100.DAY_573.PlasmacDNA.P4C21 -------------------- -------------------- -------------------- -------------------- --------------------
A19100.DAY_573.PlasmacDNA.P4C22 -------------------- -------------------- -------------------- -------------------- --------------------
 A19100.DAY_573.PlasmacDNA.P4D2 -------------------- -------------------- -------------------- -------------------- --------------------
 A19100.DAY_573.PlasmacDNA.P4D3 -------------------- -------------------- -------------------- -------------------- --------------------
 A19100.DAY_573.PlasmacDNA.P4D4 -------------------- -------------------- -------------------- -------------------- --------------------
 A19100.DAY_650.PlasmacDNA.P4I2 -------------------- -------------------- -------------------- -------------------- --------------------
 A19100.DAY_650.PlasmacDNA.P4I3 -------------------- -------------------- -------------------- -------------------- --------------------
A19100.DAY_650.PlasmacDNA.P4I20 -------------------- -------------------- -------------------- -------------------- --------------------
 A19100.DAY_650.PlasmacDNA.P4J4 -------------------- -------------------- -------------------- -------------------- --------------------
A19100.DAY_650.PlasmacDNA.P4J19 -------------------- -------------------- -------------------- -------------------- --------------------
A19100.DAY_650.PlasmacDNA.P4J22 -------------------- -------------------- -------------------- -------------------- --------------------
 A19100.DAY_650.PlasmacDNA.P4K3 -------------------- -------------------- -------------------- -------------------- --------------------
A19100.DAY_650.PlasmacDNA.P4K10 -------------------- -------------------- -------------------- -------------------- --------------------
A19100.DAY_650.PlasmacDNA.P4K12 -------------------- -------------------- -------------------- -------------------- --------------------
A19100.DAY_650.PlasmacDNA.P4K17 -------------------- -------------------- -------------------- -------------------- --------------------
A19100.DAY_650.PlasmacDNA.P4K20 -------------------- -------------------- -------------------- -------------------- --------------------
 A19100.DAY_650.PlasmacDNA.P4L7 -------------------- -------------------- -------------------- -------------------- --------------------
A19100.DAY_650.PlasmacDNA.P4L11 -------------------- -------------------- -------------------- -------------------- --------------------
 A19100.DAY_650.PlasmacDNA.P4M3 -------------------- -------------------- -------------------- -------------------- ------------H-------
 A19100.DAY_650.PlasmacDNA.P4M9 -------------------- -------------------- -------------------- -------------------- --------------------
A19100.DAY_650.PlasmacDNA.P4M16 -------------------- -------------------- -------------------- -------------------- --------------------
 A19100.DAY_650.PlasmacDNA.P4N4 -------------------- -------------------- -------------------- -------------------- --------------------
 A19100.DAY_650.PlasmacDNA.P4N7 -------------------- -------------------- -------------------- -------------------- --------------------
 A19100.DAY_650.PlasmacDNA.P4O4 -------------------- -------------------- -------------------- -------------------- --------------------
 A19100.DAY_650.PlasmacDNA.P4O6 -------------------- -------------------- -------------------- -------------------- ------------H-------
 A19100.DAY_650.PlasmacDNA.P4O7 -------------------- -------------------- -------------------- -------------------- --------------------
A19100.DAY_650.PlasmacDNA.P4O10 -------------------- -------------------- -------------------- -------------------- --------------------
A19100.DAY_650.PlasmacDNA.P4O15 -------------------- -------------------- -------------------- -------------------- --------------------
A19100.DAY_650.PlasmacDNA.P4O23 -------------------- -------------------- -------------------- -------------------- --------------------
A19100.DAY_650.PlasmacDNA.P4P17 -------------------- -------------------- -------------------- -------------------- --------------------
A19100.DAY_650.PlasmacDNA.P4P20 -------------------- -------------------- -------------------- -------------------- --------------------

                       SHIV1157 ISIGPGQAIYATGDIIGDIR QAHCNISKENWNKTLQWVRG KLKEHFPNKTIVFKPSSGGD LEITTHSFNCRGEFFYCNTS KLFNSTDNSTHMGTENNTII  400
A19100.DAY_573.PlasmacDNA.P4C13 -------------------- -------------------- -------------------- -------------------- --------------------
A19100.DAY_573.PlasmacDNA.P4C14 -------------------- -------------------- -------------------- -------------------- --------------------
A19100.DAY_573.PlasmacDNA.P4C15 -------------------- -------------------- -------------------- -------------------- --------------------
```

```
A19100.DAY_573.PlasmacDNA.P4C17 -------------------- -------------------- -------------------- -------------------- --------------------
A19100.DAY_573.PlasmacDNA.P4C19 -------------------- -------------------- -------------------- -------------------- --------------------
A19100.DAY_573.PlasmacDNA.P4C20 -------------------- -------------------- -------------------- -------------------- --------------------
A19100.DAY_573.PlasmacDNA.P4C21 -------------------- -------------------- -------------------- -------------------- --------------------
A19100.DAY_573.PlasmacDNA.P4C22 ------R------------- -------------------- -------------------- -------------------- --------------------
 A19100.DAY_573.PlasmacDNA.P4D2 -------------------- -------------------- -------------------- -------------------- --------------------
 A19100.DAY_573.PlasmacDNA.P4D3 -------------------- -------------------- -------------------- -------------------- --------------------
 A19100.DAY_573.PlasmacDNA.P4D4 -------------------- -------------------- -------------------- -----------------T-- --------------------
 A19100.DAY_650.PlasmacDNA.P4I2 -------------------- -------------------- -------------------- -------------------- --------------K-----
 A19100.DAY_650.PlasmacDNA.P4I3 -------------------- -------------------- -------------------- -------------------- --------------------
A19100.DAY_650.PlasmacDNA.P4I20 -------------------- -------------------- -------------------- -------------------- --------------------
 A19100.DAY_650.PlasmacDNA.P4J4 -------------------- -------------------- -------------------- -------------------- --------------------
A19100.DAY_650.PlasmacDNA.P4J19 -------------------- -------------------- -------------------- -------------------- --------------------
A19100.DAY_650.PlasmacDNA.P4J22 -------------------- -------------------- -------------------- -------------------- --------------------
 A19100.DAY_650.PlasmacDNA.P4K3 -------------------- -------------------- -------------------- -------------------- --------------------
A19100.DAY_650.PlasmacDNA.P4K10 -------------------- -------------------- -------------------- -------------------- --------------------
A19100.DAY_650.PlasmacDNA.P4K12 -N------------------ -------------------- -------------------- -------------------- --------------------
A19100.DAY_650.PlasmacDNA.P4K17 -------------------- -------------------- -------------------- -------------------- --------------------
A19100.DAY_650.PlasmacDNA.P4K20 -------------------- -------------------- -------------------- -------------------- --------------------
 A19100.DAY_650.PlasmacDNA.P4L7 -------------------- -------------------- -------------------- -------------------- --------------------
A19100.DAY_650.PlasmacDNA.P4L11 -------------------- -------------------- -------------------- -------------------- --------------------
 A19100.DAY_650.PlasmacDNA.P4M3 -------------------- -------------------- -------------------- -------------------- --------------------
 A19100.DAY_650.PlasmacDNA.P4M9 -------------------- -------------------- -------------------- -------------------- --------------------
A19100.DAY_650.PlasmacDNA.P4M16 -------------------- -------------------- -------------------- -------------------- --------------------
 A19100.DAY_650.PlasmacDNA.P4N4 -------------------- -------------------- ---------------P---- -------------------- --------------------
 A19100.DAY_650.PlasmacDNA.P4N7 -------------------- -------------------- -------------------- -------------------- --------------------
 A19100.DAY_650.PlasmacDNA.P4O4 -------------------- -------------------- -------------------- -------------------- --------------------
 A19100.DAY_650.PlasmacDNA.P4O6 -------------------- -------------------- -------------------- -------------------- --------------------
 A19100.DAY_650.PlasmacDNA.P4O7 -------------------- -------------------- -------------------- -------------------- --------------------
A19100.DAY_650.PlasmacDNA.P4O10 -------------------- -------------------- -------------------- ----A--------------- --------------------
A19100.DAY_650.PlasmacDNA.P4O15 -------------------- -------------------- ---------------P---- -------------------- --------------------
A19100.DAY_650.PlasmacDNA.P4O23 -------------------- -------------------- -------S------------ -------------------- --------------------
A19100.DAY_650.PlasmacDNA.P4P17 -------------------- -------------------- -------------------- -------------------- --------------------
A19100.DAY_650.PlasmacDNA.P4P20 -------------------- -------------------- ---------------P---- -------------------- --------------------

                       SHIV1157 TIPCRIKQIINMWQEVGRAM YAPPIEGNITCKSNITGLLL VRDGGWDNSTNDTETFRPGG GDMRDNWRSELYKYKVVEVK PLGIAPTKAKRRVVEREKRA  500
A19100.DAY_573.PlasmacDNA.P4C13 -------------------- -------------------- -------------------- -------------------- --------------------
A19100.DAY_573.PlasmacDNA.P4C14 -------------------- -------------------- -------------------- -------------------- --------------------
A19100.DAY_573.PlasmacDNA.P4C15 -------------------- -------------------- -------------------- -------------------- --------------------
A19100.DAY_573.PlasmacDNA.P4C17 -------------------- -------------------- -------------------- -------------------- --------------------
A19100.DAY_573.PlasmacDNA.P4C19 -------------------- -------------------- -------------------- -------------------- --------------------
A19100.DAY_573.PlasmacDNA.P4C20 -------------------- -------------------- -------------------- -------------------- --------------------
A19100.DAY_573.PlasmacDNA.P4C21 -------------------- -------------------- -------------------- -------------------- --------------------
A19100.DAY_573.PlasmacDNA.P4C22 -------------------- -------------------- -------------------- -------------------- --------------------
 A19100.DAY_573.PlasmacDNA.P4D2 -------------------- -------------------- -------------------- -------------------- --------------------
 A19100.DAY_573.PlasmacDNA.P4D3 -------------------- -------------------- -------------------- -------------------- --------------------
 A19100.DAY_573.PlasmacDNA.P4D4 -------------------- -------------------- -------------------- -------------------- --------------------
 A19100.DAY_650.PlasmacDNA.P4I2 -------------------- -------------------- -------------------- --------N----------- --------------------
 A19100.DAY_650.PlasmacDNA.P4I3 -------------------- -------------------- -------------------- --------N----------- --------------------
A19100.DAY_650.PlasmacDNA.P4I20 -------------------- -------------------- -------------------- --------N----------- --------------------
 A19100.DAY_650.PlasmacDNA.P4J4 -------------------- -------------------- -------------------- -------------------- --------------------
A19100.DAY_650.PlasmacDNA.P4J19 -------------------- -------------------- -------------------- --------N----------- --------------------
A19100.DAY_650.PlasmacDNA.P4J22 -------------------- -------------------- -------------------- --------N----------- --------------------
 A19100.DAY_650.PlasmacDNA.P4K3 -------------------- -------------------- -------------------- --------N----------- --------------------
A19100.DAY_650.PlasmacDNA.P4K10 -------------------- -------------------- -------------------- --------N----------- --------------------
A19100.DAY_650.PlasmacDNA.P4K12 -------------------- -------------------- -------------------- --------N----------- --------------------
A19100.DAY_650.PlasmacDNA.P4K17 -------------------- -------------------- -------------------- --------N----------- --------------------
A19100.DAY_650.PlasmacDNA.P4K20 -------------------- -------------------- -------------------- --------N----------- --------------------
 A19100.DAY_650.PlasmacDNA.P4L7 -------------------- -------------------- -------------------- --------N----------- --------------------
A19100.DAY_650.PlasmacDNA.P4L11 -------------------- -------------------- -------------------- --------N----------- --------------------
 A19100.DAY_650.PlasmacDNA.P4M3 -------------------- -------------------- -------------------- --------N----------- --------------------
 A19100.DAY_650.PlasmacDNA.P4M9 -------------------- -------------------- ------G------------- -------------------- --------------------
A19100.DAY_650.PlasmacDNA.P4M16 -------------------- -------------------- -------------------- --------N----------- --------------------
 A19100.DAY_650.PlasmacDNA.P4N4 -------------------- -------------------- -------------------- -------------------- --------------------
 A19100.DAY_650.PlasmacDNA.P4N7 -------------------- -------------------- -------------------- --------N----------- --------------------
 A19100.DAY_650.PlasmacDNA.P4O4 -------------------- -------------------- -------------------- --------N----------- --------------------
 A19100.DAY_650.PlasmacDNA.P4O6 -------------------- -------------------- -------------------- --------N----------- --------------------
 A19100.DAY_650.PlasmacDNA.P4O7 -------------------- -------------------- -------------------- --------N----------- --------------------
A19100.DAY_650.PlasmacDNA.P4O10 -------------------- -------------------- -------------------- -------------------- --------------------
A19100.DAY_650.PlasmacDNA.P4O15 -------------------- -------------------- -------------------- -------------------- --------------------
A19100.DAY_650.PlasmacDNA.P4O23 -------------------- -------------------- -------------------- --------N----------- --------------------
A19100.DAY_650.PlasmacDNA.P4P17 -------------------- -----------R-------- -------------------- --------N----------- --------------------
A19100.DAY_650.PlasmacDNA.P4P20 -------------------- -------------------- E------------------- -------------------- --------------------

                       SHIV1157 VGIGAVFLGFLGAAGSTMGA ASITLTVQARQLLSGIVQQQ DNLLRAIEAQQHMLQLTVWG IKQLQARVLAIERYLQDQQL LGIWGCSGKLICTTAVPWND  600
A19100.DAY_573.PlasmacDNA.P4C13 -------------------- -------------------- -------------------- ----------V--------- --------------------
A19100.DAY_573.PlasmacDNA.P4C14 -------------------- -------------------- -------------------- -------------------- --------------------
A19100.DAY_573.PlasmacDNA.P4C15 -------------------- -------------------- -------------------- ----------M--------- --------------------
A19100.DAY_573.PlasmacDNA.P4C17 -------------------- -------------------- -------------------- ----------M--------- --------------------
A19100.DAY_573.PlasmacDNA.P4C19 -------------------- -------------------- -------------------- ----------V--------- --------------------
A19100.DAY_573.PlasmacDNA.P4C20 -------------------- -------------------- -------------------- ----------V--------- --------------------
A19100.DAY_573.PlasmacDNA.P4C21 -------------------- -------------------- -------------------- ----------V--------- --------------------
A19100.DAY_573.PlasmacDNA.P4C22 -------------------- -------------------- -------------------- -------------------- --------------------
 A19100.DAY_573.PlasmacDNA.P4D2 -------------------- -------------------- -------------------- -------------------- --------------------
 A19100.DAY_573.PlasmacDNA.P4D3 -------------------- -------------------- -------------------- ----------M--------- --------------------
 A19100.DAY_573.PlasmacDNA.P4D4 -------------------- -------------------- -------------------- ----------V--------- --------------------
 A19100.DAY_650.PlasmacDNA.P4I2 -------------------- -------------------- -------------------- -------------------- --------------------
 A19100.DAY_650.PlasmacDNA.P4I3 -------------------- -------------------- -------------------- ----------V--------- --------------------
A19100.DAY_650.PlasmacDNA.P4I20 -------------------- -------------------- -------------------- -------------------- --------------------
 A19100.DAY_650.PlasmacDNA.P4J4 --M----------------- -------------------- -------------------- -------------------- --------------------
A19100.DAY_650.PlasmacDNA.P4J19 -------------------- -------------------- -------------------- ----------V--------- --------------------
A19100.DAY_650.PlasmacDNA.P4J22 -------------------- -------------------- -------------------- ----------V--------- --------------------
 A19100.DAY_650.PlasmacDNA.P4K3 -------------------- -------------------- -------------------- -------------------- --------------------
A19100.DAY_650.PlasmacDNA.P4K10 -------------------- -------------------- -------------------- ----------V--------- --------------------
A19100.DAY_650.PlasmacDNA.P4K12 -------------------- -------------------- -------------------- -------------------- --------------------
A19100.DAY_650.PlasmacDNA.P4K17 -------------------- -------------------- -------------------- ----------V--------- --------------------
A19100.DAY_650.PlasmacDNA.P4K20 -------------------- -------------------- -------------------- ----------V--------- --------------------
 A19100.DAY_650.PlasmacDNA.P4L7 -------------------- --V----------------- -------------------- ----------V--------- --------------------
A19100.DAY_650.PlasmacDNA.P4L11 -----A-------------- -------------------- -------------------- ----------V--------- --------------------
 A19100.DAY_650.PlasmacDNA.P4M3 -------------------- -------------------- -------------------- ----------V--------- --------------------
 A19100.DAY_650.PlasmacDNA.P4M9 --M----------------- -------------------- -------------------- -------------------- --------------------
A19100.DAY_650.PlasmacDNA.P4M16 -------------------- -------------------- -------------------- ----------V--------- --------------------
 A19100.DAY_650.PlasmacDNA.P4N4 -------------------- -------------------- -------------------- ----------V--------- --------------------
 A19100.DAY_650.PlasmacDNA.P4N7 -------------------- -------------------- -------------------- ----------V--------- --------------------
 A19100.DAY_650.PlasmacDNA.P4O4 -------------------- -------------------- -------------------- ----------V--------- --------------------
```

```
 A19100.DAY_650.PlasmacDNA.P4O6 -------------------- -------------------- -------------------- ----------V--------- --------------------
 A19100.DAY_650.PlasmacDNA.P4O7 -------------------- -------------------- -------------------- -------------------- --------------------
A19100.DAY_650.PlasmacDNA.P4O10 -------------------- -------------------- -------------------- ----------M--------- --------------------
A19100.DAY_650.PlasmacDNA.P4O15 -------------------- -------------------- -------------------- ----------V--------- --------------------
A19100.DAY_650.PlasmacDNA.P4O23 -----A-------------- -------------------- -------------------- ----------V--------- --------------------
A19100.DAY_650.PlasmacDNA.P4P17 -------------------- -------------------- -------------------- ----------V--------- --------------------
A19100.DAY_650.PlasmacDNA.P4P20 --M----------------- -------------------- -------------------- -------------------- --------------------

                       SHIV1157 SWSNKSQTDIWENMTWMQWD REISRHTDTIYRLLEDSQNQ QEKNEKDLLALDSWKNLWNW FSITRWLWYIKIFIMIVGGL IGLRIIFAVLSIVNRVRQGY  700
A19100.DAY_573.PlasmacDNA.P4C13 -------------------- -------------------- -------------------- -------------------- --------------------
A19100.DAY_573.PlasmacDNA.P4C14 -------------------- -------------------- -------------------- -------------------- --------------------
A19100.DAY_573.PlasmacDNA.P4C15 -------------------- -------------------- -------------------- -------------------- --------------------
A19100.DAY_573.PlasmacDNA.P4C17 -------------------- -------------------- -------------------- -------------------- --------------------
A19100.DAY_573.PlasmacDNA.P4C19 -------------------- -------------------- -------------------- -------------------- --------------------
A19100.DAY_573.PlasmacDNA.P4C20 -------------------- -------------------- -------------------- -------------------- --------------------
A19100.DAY_573.PlasmacDNA.P4C21 -------------------- -------------------- -------------------- -------------------- --------------------
A19100.DAY_573.PlasmacDNA.P4C22 -----------------R-- -------------------- -------------------- -------------------- --------------------
 A19100.DAY_573.PlasmacDNA.P4D2 -------------------- -------------------- -------------------- -------------------- --------------------
 A19100.DAY_573.PlasmacDNA.P4D3 -------------------- -------------------- -------------------- -------------------- --------------------
 A19100.DAY_573.PlasmacDNA.P4D4 -------------------- -------------------- -------------------- -------------------- M-------------------
 A19100.DAY_650.PlasmacDNA.P4I2 -------------------- -------------------- -------------------- -------------------- M-------------------
 A19100.DAY_650.PlasmacDNA.P4I3 -------------------- -------------------- -------------------- -------------------- --------------------
A19100.DAY_650.PlasmacDNA.P4I20 -------------------- -------------------- -------------------- -------------------- --------------------
 A19100.DAY_650.PlasmacDNA.P4J4 -------------------- -------------------- -------------------- -------------------- --------------------
A19100.DAY_650.PlasmacDNA.P4J19 -------------------- -------------------- -------------------- -------------------- --------------------
A19100.DAY_650.PlasmacDNA.P4J22 -------------------- -------------------- -------------------- -------------------- --------------------
 A19100.DAY_650.PlasmacDNA.P4K3 -------------------- -------------------- -------------------- -------------------- --------------------
A19100.DAY_650.PlasmacDNA.P4K10 -------------------- -------------------- -------------------- -------------------- --------------------
A19100.DAY_650.PlasmacDNA.P4K12 -------------------- -------------------- -------------------- -------------------- --------------------
A19100.DAY_650.PlasmacDNA.P4K17 -------------------- -------------------- -------------------- -------------------- --------------------
A19100.DAY_650.PlasmacDNA.P4K20 -------------------- -------------------- -------------------- -------------------- --------------------
 A19100.DAY_650.PlasmacDNA.P4L7 -------------------- -------------------- -------------------- -------------------- --------------------
A19100.DAY_650.PlasmacDNA.P4L11 -------------------- -------------------- -------------------- -------------------- M-------------------
 A19100.DAY_650.PlasmacDNA.P4M3 -------------------- -------------------- -------------------- -------------------- --------------------
 A19100.DAY_650.PlasmacDNA.P4M9 -------------------- -------------------- -------------------- -------------------- --------------------
A19100.DAY_650.PlasmacDNA.P4M16 -------------------- -------------------- -------------------- -------------------- --------------------
 A19100.DAY_650.PlasmacDNA.P4N4 -------------------- -------------------- -------------------- -------------------- M-------------------
 A19100.DAY_650.PlasmacDNA.P4N7 -------------------- -------------------- -------------------- -------------------- --------------------
 A19100.DAY_650.PlasmacDNA.P4O4 -------------------- -------------------- -------------------- -------------------- --------------------
 A19100.DAY_650.PlasmacDNA.P4O6 -------------------- -------------------- -------------------- -------------------- --------------------
 A19100.DAY_650.PlasmacDNA.P4O7 -------------------- -------------------- -------------------- -------------------- --------------------
A19100.DAY_650.PlasmacDNA.P4O10 -------------------- -------------------- -------------------- -------------------- --------------------
A19100.DAY_650.PlasmacDNA.P4O15 -------------------- -------------------- -------------------- -------------------- M-------------------
A19100.DAY_650.PlasmacDNA.P4O23 -------------------- -------------------- -------------------- -------------------- M-------------------
A19100.DAY_650.PlasmacDNA.P4P17 -------------------- -------------------- -------------------- -------------------- --------------------
A19100.DAY_650.PlasmacDNA.P4P20 -------------------- -------------------- -------------------- -------------------- --------------------

                       SHIV1157 SPLSFQTHLPLPRGADRPEG IEEEGGERDRDRSIRLVTGS LALIWDDLRSLCLFSYHRLR DLLLIVTRTVELLGRRGWEA LKYWWNLLLYWSQELKNSAV  800
A19100.DAY_573.PlasmacDNA.P4C13 -------------------- -------------------- -------------------- -------------------- --------------------
A19100.DAY_573.PlasmacDNA.P4C14 -------------------- -------------------- -------------------- -------------------- --------------------
A19100.DAY_573.PlasmacDNA.P4C15 -------------------- -------------------- -------------------- -------------------- --------------------
A19100.DAY_573.PlasmacDNA.P4C17 -------------------- -------------------- -------------------- -------------------- --------------------
A19100.DAY_573.PlasmacDNA.P4C19 -------------------- -------------------- -------------------- -------------------- --------------------
A19100.DAY_573.PlasmacDNA.P4C20 -------------------- -------------------- -------------------- -------------------- --------------------
A19100.DAY_573.PlasmacDNA.P4C21 -------------------- -------------------- -------------------- -------------------- --------------------
A19100.DAY_573.PlasmacDNA.P4C22 -------------------- -------------------- -------------------- -------------------- --------------------
 A19100.DAY_573.PlasmacDNA.P4D2 -------------------- -------------------- -------------------- -------------------- --------------------
 A19100.DAY_573.PlasmacDNA.P4D3 -------------------- -------------------- -------------------- -------------------- --------------------
 A19100.DAY_573.PlasmacDNA.P4D4 -------------------- -------------------- -------------------- -------------------- --------------------
 A19100.DAY_650.PlasmacDNA.P4I2 -------------------- -------------------- -------------------- -------------------- --------------------
 A19100.DAY_650.PlasmacDNA.P4I3 -------------------- -------------------- -------------------- -------------------- --------------------
A19100.DAY_650.PlasmacDNA.P4I20 -------------------- -------------------- -------------------- -------------------- --------------------
 A19100.DAY_650.PlasmacDNA.P4J4 -------------------- -------------------- -------------------- -------------------- --------------------
A19100.DAY_650.PlasmacDNA.P4J19 -------------------- -------------------- -------------------- -------------------- --------------------
A19100.DAY_650.PlasmacDNA.P4J22 -------------------- -------------------- -------------------- -------------------- --------------------
 A19100.DAY_650.PlasmacDNA.P4K3 -------------------- -------------------- -------------------- -------------------- --------------------
A19100.DAY_650.PlasmacDNA.P4K10 -------------------- -------------------- -------------------- -------------------- --------------------
A19100.DAY_650.PlasmacDNA.P4K12 -------------------- -------------------- -------------------- -------------------- --------------------
A19100.DAY_650.PlasmacDNA.P4K17 -------------------- -------------------- -------------------- -------------------- --------------------
A19100.DAY_650.PlasmacDNA.P4K20 -------------------- -------------------- -------------------- -------------------- --------------------
 A19100.DAY_650.PlasmacDNA.P4L7 -------------------- -------------------- -------------------- -------------------- --------------------
A19100.DAY_650.PlasmacDNA.P4L11 -------------------- -------------------- -------------------- -------------------- --------------------
 A19100.DAY_650.PlasmacDNA.P4M3 -------------------- -------------------- -------------------- -------------------- --------------------
 A19100.DAY_650.PlasmacDNA.P4M9 -------------------- -------------------- -------------------- -------------------- --------------------
A19100.DAY_650.PlasmacDNA.P4M16 -------------------- -------------------- -------------------- -------------------- --------------------
 A19100.DAY_650.PlasmacDNA.P4N4 -------------------- -------------------- -------------------- -------------------- --------------------
 A19100.DAY_650.PlasmacDNA.P4N7 -------------------- -------------------- -------------------- -------------------- --------------------
 A19100.DAY_650.PlasmacDNA.P4O4 -------------------- -------------------- -------------------- -------------------- --------------------
 A19100.DAY_650.PlasmacDNA.P4O6 -------------------- -------------------- -------------------- -------------------- --------------------
 A19100.DAY_650.PlasmacDNA.P4O7 -------------------- -------------------- -------------------- -------------------- --------------------
A19100.DAY_650.PlasmacDNA.P4O10 -------------------- -------------------- -------------------- -------------------- --------------------
A19100.DAY_650.PlasmacDNA.P4O15 -------------------- -------------------- -------------------- -------------------- --------------------
A19100.DAY_650.PlasmacDNA.P4O23 -------------------- -------------------- -------------------- -------------------- --------------------
A19100.DAY_650.PlasmacDNA.P4P17 -------------------- -------------------- -------------------- -------------------- --------------------
A19100.DAY_650.PlasmacDNA.P4P20 -------------------- -------------------- -------------------- -------------------- --------------------

                       SHIV1157 SLLNATAIAVRQYGWSYFHE AVQAVWRSATETLAGAWGDL WEILRRGGRWILAIPRRIRQ GLELTLL*  868
A19100.DAY_573.PlasmacDNA.P4C13 -------------------- -------------------- --T----------------- --------
A19100.DAY_573.PlasmacDNA.P4C14 -------------------- -------------------- -------------------- --------
A19100.DAY_573.PlasmacDNA.P4C15 -------------------- -------------------- -------------------- --------
A19100.DAY_573.PlasmacDNA.P4C17 -------------------- -------------------- -------------------- ------W-
A19100.DAY_573.PlasmacDNA.P4C19 -------------------- -------------------- --T----------------- --------
A19100.DAY_573.PlasmacDNA.P4C20 -------------------- -------------------- --T----------------- --------
A19100.DAY_573.PlasmacDNA.P4C21 -------------------- -------------------- --T----------------- --------
A19100.DAY_573.PlasmacDNA.P4C22 -------------------- -------------------- -------------------- --------
 A19100.DAY_573.PlasmacDNA.P4D2 -------------------- -------------------- -------------------- --------
 A19100.DAY_573.PlasmacDNA.P4D3 -------------------- -------------------- -------------------- --------
 A19100.DAY_573.PlasmacDNA.P4D4 -------------------- -------------------- --T----------------- --------
 A19100.DAY_650.PlasmacDNA.P4I2 -------------------- -------------------- --T----------------- --------
 A19100.DAY_650.PlasmacDNA.P4I3 -------------------- -------------------- --T----------------- --------
A19100.DAY_650.PlasmacDNA.P4I20 -------------------- -------------------- --T----------------- --------
 A19100.DAY_650.PlasmacDNA.P4J4 -------------------- -------------------- -------------------- --------
A19100.DAY_650.PlasmacDNA.P4J19 -------------------- -------------------- --T----------------- --------
A19100.DAY_650.PlasmacDNA.P4J22 -------------------- -------------------- --T----------------- --------
 A19100.DAY_650.PlasmacDNA.P4K3 -------------------- -------------------- --T----------------- --------
```

```
A19100.DAY_650.PlasmacDNA.P4K10 -------------------- ---V---------------- --T----------------- --------
A19100.DAY_650.PlasmacDNA.P4K12 -------------------- ---V---------------- --T----------------- --------
A19100.DAY_650.PlasmacDNA.P4K17 -------------------- -------------------- --T----------------- --------
A19100.DAY_650.PlasmacDNA.P4K20 -------------------- -------------------- --T----------------- --------
 A19100.DAY_650.PlasmacDNA.P4L7 -------------------- -------------------- --T----------------- --------
A19100.DAY_650.PlasmacDNA.P4L11 -------------------- -------------------- --T----------------- --------
 A19100.DAY_650.PlasmacDNA.P4M3 -------------------- -------------------- --T----------------- --------
 A19100.DAY_650.PlasmacDNA.P4M9 -------------------- -------------------- -------------------- --------
A19100.DAY_650.PlasmacDNA.P4M16 -------------------- -------------------- --T----------------- --------
 A19100.DAY_650.PlasmacDNA.P4N4 -------------------- ---V---------------- --T----------------- --------
 A19100.DAY_650.PlasmacDNA.P4N7 -------------------- -------------------- --T----------------- --------
 A19100.DAY_650.PlasmacDNA.P4O4 -------------------- -------------------- --T----------------- --------
 A19100.DAY_650.PlasmacDNA.P4O6 -------------------- -------------------- --T----------------- --------
 A19100.DAY_650.PlasmacDNA.P4O7 -------------------- -------------------- --T----------------- --------
A19100.DAY_650.PlasmacDNA.P4O10 -------------------- -------------------- -------------------- --------
A19100.DAY_650.PlasmacDNA.P4O15 -------------------- ---V---------------- --T----------------- --------
A19100.DAY_650.PlasmacDNA.P4O23 -------------------- -------------------- --T----------------- --------
A19100.DAY_650.PlasmacDNA.P4P17 -------------------- -------------------- --T----------------- --------
A19100.DAY_650.PlasmacDNA.P4P20 -------------------- -------------------- -------------------- --------
```

A19101 Control

```
                       SHIV1157 MRVKEKYQHLWRWGWRWGIM LLGMLMICSASEKLWVTVYY GVPVWKEAKTTLFCASNAKA YEKEVHNIWATHACVPTDPN PQEIVLGNVTENFNMWKNDM  100
 A19101.DAY_650.PlasmacDNA.P3M9 -------------------- -------------------- -------------------- -------------------- --------------------
A19101.DAY_650.PlasmacDNA.P3M17 -------------------- -------------------- -------------------- -------------------- --------------------
A19101.DAY_650.PlasmacDNA.P3M24 -------------------- -------------------- -------------------- -------------------- --------------------
A19101.DAY_650.PlasmacDNA.P3N10 -------------------- -------------------- -------------------- -------------------- --------------------
A19101.DAY_650.PlasmacDNA.P3N14 -------------------- -------------------- -------------------- -------------------- --------------------
 A19101.DAY_650.PlasmacDNA.P4A5 -------------------- -------------------- -------------------- -------------------- --------------------
A19101.DAY_650.PlasmacDNA.P4A11 -------------------- -------------------- -------------------- -------------------- --------------------
A19101.DAY_650.PlasmacDNA.P4A14 -------------------- -------------------- -------------------- -------------------- --------------------
 A19101.DAY_650.PlasmacDNA.P4B2 -------------------- -------------------- -------------------- -------------------- --------------------
 A19101.DAY_650.PlasmacDNA.P4B8 -------------------- -------------------- -------------------- -------------------- --------------------
A19101.DAY_650.PlasmacDNA.P4B10 -------------------- -------------------- -------------------- -------------------- --------------------
A19101.DAY_650.PlasmacDNA.P4B22 -------------------- -------------------- -------------------- -------------------- --------------------
 A19101.DAY_650.PlasmacDNA.P4C3 -------------------- -------------------- -------------------- -------------------- --------------------
 A19101.DAY_650.PlasmacDNA.P4C5 -------------------- -------------------- -------------------- -------------------- --------------------
 A19101.DAY_650.PlasmacDNA.P4C6 -------------------- -------------------- -------------------- -------------------- --------------------
A19101.DAY_650.PlasmacDNA.P4C21 -------------------- -------------------- -------------------- -------------------- --------------------
 A19101.DAY_650.PlasmacDNA.P4D3 -------------------- -------------------- -------------------- -------------------- --------------------
 A19101.DAY_650.PlasmacDNA.P4D5 -------------------- -------------------- -------------------- -------------------- --------------------
A19101.DAY_650.PlasmacDNA.P4D15 -------------------- -------------------- -------------------- -------------------- --------------------
 A19101.DAY_650.PlasmacDNA.P4E2 -------------------- -------------------- -------------------- -------------------- --------------------
 A19101.DAY_650.PlasmacDNA.P4E8 -------------------- -------------------- -------------------- -------------------- --------------------
 A19101.DAY_650.PlasmacDNA.P4E9 -------------------- -------------------- -------------------- -------------------- --------------------
 A19101.DAY_650.PlasmacDNA.P4F6 -------------------- -------------------- -------------------- -------------------- --------------------
 A19101.DAY_650.PlasmacDNA.P4F7 -------------------- -------------------- -------------------- -------------------- --------------------
A19101.DAY_650.PlasmacDNA.P4F10 -------------------- -------------------- -------------------- -------------------- --------------------
A19101.DAY_650.PlasmacDNA.P4F12 -------------------- -------------------- -------------------- -------------------- --------------------
 A19101.DAY_650.PlasmacDNA.P4G3 -------------------- -------------------- -------------------- -------------------- --------------------
A19101.DAY_650.PlasmacDNA.P4G22 -------------------- -------------------- -------------------- -------------------- --------------------
A19101.DAY_650.PlasmacDNA.P4H10 -------------------- -------------------- -------------------- -------------------- --------------------

                       SHIV1157 VDQMHEDIISLWDQSLKPCV KLTSLCVTLKCSNFTGKSNV TYKGDMEVKNCSFNVTTEIR DKKQKVYALFYRLDITPLDD NSSEYILINCNSSTITQACP  200
 A19101.DAY_650.PlasmacDNA.P3M9 -------------------- -------------------- -------------------- -------------------- --------------------
A19101.DAY_650.PlasmacDNA.P3M17 -------------------- -------------------- -------------------- -------------------- --------------------
A19101.DAY_650.PlasmacDNA.P3M24 -------------------- -------------------- -------------------- N------------------- --------------------
A19101.DAY_650.PlasmacDNA.P3N10 -------------------- -------------------- -------------------- -------------------- --------------------
A19101.DAY_650.PlasmacDNA.P3N14 -------------------- -------------------- -------------------- -------------------- --------------------
 A19101.DAY_650.PlasmacDNA.P4A5 -------------------- -------------------- -------------------- -------------------- ----------------R---
A19101.DAY_650.PlasmacDNA.P4A11 -------------------- -------------------- -------------------- -------------------- --------------------
A19101.DAY_650.PlasmacDNA.P4A14 -------------------- -------------------- -------------------- -------------------- --------------------
 A19101.DAY_650.PlasmacDNA.P4B2 -------------------- -------------------- -------------------- -------------------- --------------------
 A19101.DAY_650.PlasmacDNA.P4B8 -------------------- -------------------- -------------------- -------------------- --------------------
A19101.DAY_650.PlasmacDNA.P4B10 -------------------- -------------------- -------------------- -------------------- --------------------
A19101.DAY_650.PlasmacDNA.P4B22 -------------------- -------------------- -------------------- -------------------- --------------------
 A19101.DAY_650.PlasmacDNA.P4C3 -------------------- -------------------- -------------------- -------------------- --------------------
 A19101.DAY_650.PlasmacDNA.P4C5 -------------------- -------------------- -------------------- -------------------- --------------------
 A19101.DAY_650.PlasmacDNA.P4C6 -------------------- -------------------- -------------------- -------------------- --------------------
A19101.DAY_650.PlasmacDNA.P4C21 -------------------- -------------------- -------------------- -------------------- --------------------
 A19101.DAY_650.PlasmacDNA.P4D3 -------------------- -------------------- -------------------- -------------------- --------------------
 A19101.DAY_650.PlasmacDNA.P4D5 -------------------- -------------------- -------------------- -------------------- --------------------
A19101.DAY_650.PlasmacDNA.P4D15 -------------------- -------------------- -------------------- -------------------- --------------------
 A19101.DAY_650.PlasmacDNA.P4E2 -------------------- -------------------- -------------------- -------------------- --------------------
 A19101.DAY_650.PlasmacDNA.P4E8 -------------------- -------------------- -------------------- -------------------- --------------------
 A19101.DAY_650.PlasmacDNA.P4E9 -------------------- -------------------- -------------------- -------------------- --------------------
 A19101.DAY_650.PlasmacDNA.P4F6 -------------------- -------------------- -------------------- -------------------- --------------------
 A19101.DAY_650.PlasmacDNA.P4F7 -------------------- -------------------- -------------------- -------------------- --------------------
A19101.DAY_650.PlasmacDNA.P4F10 -------------------- -------------------- -------------------- N------------------- --------------------
A19101.DAY_650.PlasmacDNA.P4F12 -------------------- -------------------- -------------------- -------------------- --------------------
 A19101.DAY_650.PlasmacDNA.P4G3 -------------------- -------------------- -------------------- -------------------- --------------------
A19101.DAY_650.PlasmacDNA.P4G22 -------------------- -------------------- -------------------- -------------------- --------------------
A19101.DAY_650.PlasmacDNA.P4H10 -------------------- -------------------- -------------------- -------------------- --------------------

                       SHIV1157 KVNFDPIPIHYCAPAGYAIL KCNNKTFNGTGPCHNVSTVQ CTHGIKPVVSTQLLLNGSLA EGEIIIRSENLTDNVKTIIV HFNESVEITCTRPNNNTRKS  300
 A19101.DAY_650.PlasmacDNA.P3M9 -------------------- -------------------- -------------------- -------------------- --------------------
A19101.DAY_650.PlasmacDNA.P3M17 -------------------- -------------------- -------------------- -------------------- --------------------
A19101.DAY_650.PlasmacDNA.P3M24 -------------------- -------------------- -------------------- -------------------- --------------------
A19101.DAY_650.PlasmacDNA.P3N10 -------------------- -------------------- -------------------- -------------------- --------------------
A19101.DAY_650.PlasmacDNA.P3N14 -------------------- -------------------- -------------------- -------------------- --------------------
 A19101.DAY_650.PlasmacDNA.P4A5 -------------------- -------------------- -------------------- -------------------- --------------------
A19101.DAY_650.PlasmacDNA.P4A11 -------------------- -------------------- -------------------- -------------------- --------------------
A19101.DAY_650.PlasmacDNA.P4A14 -------------------- -------------------- -------------------- -------------------- --------------------
 A19101.DAY_650.PlasmacDNA.P4B2 -------------------- -------------------- -------------------- -------------------- --------------------
 A19101.DAY_650.PlasmacDNA.P4B8 -------------------- -------------------- -------------------- -------------------- --------------------
A19101.DAY_650.PlasmacDNA.P4B10 -------------------- -------------------- -------------------- -------------------- --------------------
A19101.DAY_650.PlasmacDNA.P4B22 -------------------- -------------------- -------------------- -------------------- --------------------
 A19101.DAY_650.PlasmacDNA.P4C3 -------------------- -------------------- -------------------- -------------------- --------------------
 A19101.DAY_650.PlasmacDNA.P4C5 -------------------- -------------------- -------------------- -------------------- --------------------
 A19101.DAY_650.PlasmacDNA.P4C6 -------------------- -------------------- -------------------- -------------------- --------------------
A19101.DAY_650.PlasmacDNA.P4C21 -------------------- -------------------- -------------------- -------------------- --------------------
 A19101.DAY_650.PlasmacDNA.P4D3 -------------------- -------------------- -------------------- -------------------- --------------------
 A19101.DAY_650.PlasmacDNA.P4D5 -------------------- -------------------- -------------------- -------------------- --------------------
A19101.DAY_650.PlasmacDNA.P4D15 -------------------- -------------------- -------------------- -------------------- --------------------
 A19101.DAY_650.PlasmacDNA.P4E2 -------------------- -------------------- -------------------- -------------------- --------------------
 A19101.DAY_650.PlasmacDNA.P4E8 -------------------- -------------------- -------------------- -------------------- --------------------
```

```
 A19101.DAY_650.PlasmacDNA.P4E9 -------------------- -------------------- -------------------- -------------------- --------------------
 A19101.DAY_650.PlasmacDNA.P4F6 -------------------- -------------------- -------------------- -------------------- --------------------
 A19101.DAY_650.PlasmacDNA.P4F7 -------------------- -------------------- -------------------- -------------------- --------------------
A19101.DAY_650.PlasmacDNA.P4F10 -------------------- -------------------- -------------------- -------------------- --------------------
A19101.DAY_650.PlasmacDNA.P4F12 -------------------- -------------------- -------------------- -------------------- --------------------
 A19101.DAY_650.PlasmacDNA.P4G3 -------------------- -------------------- -------------------- -------------------- --------------------
A19101.DAY_650.PlasmacDNA.P4G22 -------------------- -------------------- -------------------- -------------------- --------------------
A19101.DAY_650.PlasmacDNA.P4H10 -------------------- -------------------- -------------------- -------------------- --------------------

                       SHIV1157 ISIGPGQAIYATGDIIGDIR QAHCNISKENWNKTLQWVRG KLKEHFPNKTIVFKPSSGGD LEITTHSFNCRGEFFYCNTS KLFNSTDNSTHMGTENNTII  400
 A19101.DAY_650.PlasmacDNA.P3M9 -------------------- -------------------- -------------------- -----------------D-- --------------------
A19101.DAY_650.PlasmacDNA.P3M17 -------------------- -------------------- -------------------- -----------------D-- --------------------
A19101.DAY_650.PlasmacDNA.P3M24 -------------------- -------------------- -------------------- -----------------D-- --------------------
A19101.DAY_650.PlasmacDNA.P3N10 -------------------- -------------------- -------------------- -----------------D-- --------------------
A19101.DAY_650.PlasmacDNA.P3N14 -------------------- -------------------- -------------------- -----------------D-- --------------D-----
 A19101.DAY_650.PlasmacDNA.P4A5 -------------------- -------------------- -------------------- -----------------D-- --------------------
A19101.DAY_650.PlasmacDNA.P4A11 -------------------- -------------------- -------------------- -----------------D-- --------------------
A19101.DAY_650.PlasmacDNA.P4A14 -------------------- -------------------- -------------------- -----------------D-- --------------------
 A19101.DAY_650.PlasmacDNA.P4B2 -------------------- -------------------- -------------------- -----------------D-- --------------------
 A19101.DAY_650.PlasmacDNA.P4B8 -------------------- -------------------- -------------------- -----------------D-- --------------------
A19101.DAY_650.PlasmacDNA.P4B10 -------------------- -------------------- -------------------- -----------------D-- --------------------
A19101.DAY_650.PlasmacDNA.P4B22 -------------------- -------------------- -------------------- -----------------D-- --------------------
 A19101.DAY_650.PlasmacDNA.P4C3 -------------------- -------------------- -------------------- -----------------D-- --------------------
 A19101.DAY_650.PlasmacDNA.P4C5 -------------------- -------------------- -------------------- -----------------D-- --------------------
 A19101.DAY_650.PlasmacDNA.P4C6 -------------------- -------------------- -------------------- -----------------D-- --------------------
A19101.DAY_650.PlasmacDNA.P4C21 -------------------- -------------------- -------------------- -----------------D-- --------------------
 A19101.DAY_650.PlasmacDNA.P4D3 -------------------- -------------------- -------------------- -----------------D-- --------------------
 A19101.DAY_650.PlasmacDNA.P4D5 -------------------- -------------------- -------------------- -----------------D-- --------------------
A19101.DAY_650.PlasmacDNA.P4D15 -------------------- -------------------- -------------------- -----------------D-- --------------------
 A19101.DAY_650.PlasmacDNA.P4E2 -------------------- -------------------- -------------------- -----------------D-- ---------------S----
 A19101.DAY_650.PlasmacDNA.P4E8 -------------------- -------------------- -------------------- -----------------D-- --------------------
 A19101.DAY_650.PlasmacDNA.P4E9 -------------------- -------------------- -------------------- -----------------D-- --------------------
 A19101.DAY_650.PlasmacDNA.P4F6 -------------------- -------------------- -------------------- -----------------D-- --------------------
 A19101.DAY_650.PlasmacDNA.P4F7 -------------------- -------------------- -------------------- -----------------D-- --------------------
A19101.DAY_650.PlasmacDNA.P4F10 -------------------- -------------------- -------------------- -----------------D-- --------------------
A19101.DAY_650.PlasmacDNA.P4F12 -------------------- -------------------- -------------------- -----------------D-- --------------------
 A19101.DAY_650.PlasmacDNA.P4G3 -------------------- -------------------- -------------------- -----------------D-- ---------------S----
A19101.DAY_650.PlasmacDNA.P4G22 -------------------- -------------------- -------------------- -----------------D-- --------------------
A19101.DAY_650.PlasmacDNA.P4H10 -------------------- -------------------- -------------------- -----------------D-- --------------------

                       SHIV1157 TIPCRIKQIINMWQEVGRAM YAPPIEGNITCKSNITGLLL VRDGGWDNSTNDTETFRPGG GDMRDNWRSELYKYKVVEVK PLGIAPTKAKRRVVEREKRA  500
 A19101.DAY_650.PlasmacDNA.P3M9 -------------------- -------------------- -------------------- -------------------- --------------------
A19101.DAY_650.PlasmacDNA.P3M17 -------------------- -------------------- -------------------- -------------------- --------------------
A19101.DAY_650.PlasmacDNA.P3M24 -------------------- -------------------- -------------------- -------K------------ --------------------
A19101.DAY_650.PlasmacDNA.P3N10 -------------------- -------------------- -------------------- -------------------- --------------------
A19101.DAY_650.PlasmacDNA.P3N14 -------------------- -------------------- -------------------- -------------------- --------------------
 A19101.DAY_650.PlasmacDNA.P4A5 -------------------- -------------------- -------------------- -------------------- --------------------
A19101.DAY_650.PlasmacDNA.P4A11 -------------------- -------------------- -------------------- -------------------- --------------------
A19101.DAY_650.PlasmacDNA.P4A14 -------------------- -------------------- -------------------- -------------------- --------------------
 A19101.DAY_650.PlasmacDNA.P4B2 -------------------- -------------------- -------------------- -------------------- --------------------
 A19101.DAY_650.PlasmacDNA.P4B8 -------------------- -------------------- -------------------- -------------------- --------------------
A19101.DAY_650.PlasmacDNA.P4B10 -------------------- -------------------- -------------------- -------------------- --------------------
A19101.DAY_650.PlasmacDNA.P4B22 -------------------- -------------------- -------------------- -------------------- --------------------
 A19101.DAY_650.PlasmacDNA.P4C3 -------------------- -------------------- -------------------- -------------------- --------------------
 A19101.DAY_650.PlasmacDNA.P4C5 -------------------- -------------------- -------------------- -------------------- --------------------
 A19101.DAY_650.PlasmacDNA.P4C6 -------------------- -------------------- -------------------- -------------------- --------------------
A19101.DAY_650.PlasmacDNA.P4C21 -------------------- -------------------- -------------------- -------------------- --------------------
 A19101.DAY_650.PlasmacDNA.P4D3 -------------------- -------------------- -------------------- -------------------- --------------------
 A19101.DAY_650.PlasmacDNA.P4D5 -------------------- -------------------- -------------------- -------------------- --------------------
A19101.DAY_650.PlasmacDNA.P4D15 -------------------- -------------------- -------------------- -------------------- --------------------
 A19101.DAY_650.PlasmacDNA.P4E2 -------------------- -------------------- -------------------- -------------------- --------------------
 A19101.DAY_650.PlasmacDNA.P4E8 -------------------- -------------------- -------------------- -------------------- --------------------
 A19101.DAY_650.PlasmacDNA.P4E9 -------------------- -------------------- -------------------- -------------------- --------------------
 A19101.DAY_650.PlasmacDNA.P4F6 -------------------- -------------------- -------------------- -------------------- --------------------
 A19101.DAY_650.PlasmacDNA.P4F7 -------------------- -------------------- -------------------- -------------------- --------------------
A19101.DAY_650.PlasmacDNA.P4F10 -------------------- -------------------- -------------------- -------K------------ --------------------
A19101.DAY_650.PlasmacDNA.P4F12 -------------------- -------------------- -------------------- -------------------- --------------------
 A19101.DAY_650.PlasmacDNA.P4G3 -------------------- -------------------- -------------------- -------------------- --------------------
A19101.DAY_650.PlasmacDNA.P4G22 -------------------- -------------------- -------------------- -------------------- --------------------
A19101.DAY_650.PlasmacDNA.P4H10 -------------------- -------------------- -------------------- -------------------- --------------------

                       SHIV1157 VGIGAVFLGFLGAAGSTMGA ASITLTVQARQLLSGIVQQQ DNLLRAIEAQQHMLQLTVWG IKQLQARVLAIERYLQDQQL LGIWGCSGKLICTTAVPWND  600
 A19101.DAY_650.PlasmacDNA.P3M9 -------------------- -------------------- -------------------- ----------V--------- --------------------
A19101.DAY_650.PlasmacDNA.P3M17 -------------------- -------------------- -------------------- ----------V--------- --------------------
A19101.DAY_650.PlasmacDNA.P3M24 -------------------- -------------------- -------------------- ----------V--------- --------------------
A19101.DAY_650.PlasmacDNA.P3N10 -------------------- -------------------- -------------------- ----------V--------- --------------------
A19101.DAY_650.PlasmacDNA.P3N14 -------------------- -------------------- -------------------- ----------V--------- --------------------
 A19101.DAY_650.PlasmacDNA.P4A5 -------------------- -------------------- -------------------- ----------V--------- --------------------
A19101.DAY_650.PlasmacDNA.P4A11 -------------------- -------------------- -------------------- ----------V--------- --------------------
A19101.DAY_650.PlasmacDNA.P4A14 -------------------- -------------------- -------------------- ----------V--------- --------------------
 A19101.DAY_650.PlasmacDNA.P4B2 -------------------- -------------------- -------------------- ----------V--------- --------------------
 A19101.DAY_650.PlasmacDNA.P4B8 -------------------- -------------------- -------------------- ----------V--------- --------------------
A19101.DAY_650.PlasmacDNA.P4B10 -------------------- -------------------- -------------------- ----------V--------- --------------------
A19101.DAY_650.PlasmacDNA.P4B22 -------------------- -------------------- -------------------- ----------V--------- --------------------
 A19101.DAY_650.PlasmacDNA.P4C3 -------------------- -------------------- -------------------- ----------V--------- --------------------
 A19101.DAY_650.PlasmacDNA.P4C5 -------------------- -------------------- -------------------- ----------V--------- --------------------
 A19101.DAY_650.PlasmacDNA.P4C6 -------------------- -------------------- -------------------- ----------V--------- --------------------
A19101.DAY_650.PlasmacDNA.P4C21 -------------------- -------------------- -------------------- ----------V--------- --------------------
 A19101.DAY_650.PlasmacDNA.P4D3 -------------------- -------------------- -------------------- ----------V--------- --------------------
 A19101.DAY_650.PlasmacDNA.P4D5 -------------------- -------------------- -------------------- ----------V--------- --------------------
A19101.DAY_650.PlasmacDNA.P4D15 -------------------- -------------------- -------------------- ----------V--------- --------------------
 A19101.DAY_650.PlasmacDNA.P4E2 -------------------- -------------------- -------------------- ----------V--------- --------------------
 A19101.DAY_650.PlasmacDNA.P4E8 -------------------- -------------------- -------------------- ----------V--------- --------------------
 A19101.DAY_650.PlasmacDNA.P4E9 -------------------- -------------------- -------------------- ----------V--------- --------------------
 A19101.DAY_650.PlasmacDNA.P4F6 -------------------- -------------------- -------------------- ----------V--------- --------------------
 A19101.DAY_650.PlasmacDNA.P4F7 -------------------- -------------------- -------------------- ----------V--------- --------------------
A19101.DAY_650.PlasmacDNA.P4F10 -------------------- -------------------- -------------------- ----------V--------- --------------------
A19101.DAY_650.PlasmacDNA.P4F12 -------------------- -------------------- -------------------- ----------V--------- --------------------
 A19101.DAY_650.PlasmacDNA.P4G3 -------------------- -------------------- -------------------- ----------V--------- --------------------
A19101.DAY_650.PlasmacDNA.P4G22 -------------------- -------------------- -------------------- ----------V--------- --------------------
A19101.DAY_650.PlasmacDNA.P4H10 -------------------- -------------------- -------------------- ----------V--------- --------------------
```

```
                    SHIV1157 SWSNKSQTDIWENMTWMQWD REISRHTDTIYRLLEDSQNQ QEKNEKDLLALDSWKNLWNW FSITRWLWYIKIFIMIVGGL IGLRIIFAVLSIVNRVRQGY  700
 A19101.DAY_650.PlasmacDNA.P3M9 -------------------- -------------------- -------------------- -------------------- --------------------
A19101.DAY_650.PlasmacDNA.P3M17 -------------------- -------------------- -------------------- -------------------- --------------------
A19101.DAY_650.PlasmacDNA.P3M24 -------------------- -------------------- -------------------- --------------I----- --------------------
A19101.DAY_650.PlasmacDNA.P3N10 -------------------- -------------------- -------------------- -------------------- --------M-----------
A19101.DAY_650.PlasmacDNA.P3N14 -------------------- -------------------- -------------------- -------------------- --------------------
 A19101.DAY_650.PlasmacDNA.P4A5 -------------------- -------------------- -------------------- -------------------- --------------------
A19101.DAY_650.PlasmacDNA.P4A11 -------------------- -------------------- -------------------- -------------------- --------------------
A19101.DAY_650.PlasmacDNA.P4A14 -------------------- -------------------- -------------------- -------------------- --------------------
 A19101.DAY_650.PlasmacDNA.P4B2 -------------------- -------------------- -------------------- -------------------- --------------------
 A19101.DAY_650.PlasmacDNA.P4B8 -------------------- -------------------- -------------------- -------------------- --------------------
A19101.DAY_650.PlasmacDNA.P4B10 -------------------- -------------------- -------------------- -------------------- --------------------
A19101.DAY_650.PlasmacDNA.P4B22 -------------------- -------------------- -------------------- -------------------- --------------------
 A19101.DAY_650.PlasmacDNA.P4C3 -------------------- -------------------- -------------------- -------------------- --------------------
 A19101.DAY_650.PlasmacDNA.P4C5 -------------------- -------------------- -------------------- -------------------- --------------------
 A19101.DAY_650.PlasmacDNA.P4C6 -------------------- -------------------- -------------L------ -------------------- --------------------
A19101.DAY_650.PlasmacDNA.P4C21 -------------------- -------------------- -------------------- -------------------- --------------------
 A19101.DAY_650.PlasmacDNA.P4D3 -------------------- -------------------- -------------------- -------------------- --------------------
 A19101.DAY_650.PlasmacDNA.P4D5 -------------------- -------------------- -------------------- -------------------- --------------------
A19101.DAY_650.PlasmacDNA.P4D15 -------------------- -------------------- -------------------- -------------------- --------------------
 A19101.DAY_650.PlasmacDNA.P4E2 -------------------- -------------------- -------------------- -------------------- --------------------
 A19101.DAY_650.PlasmacDNA.P4E8 -------------------- -------------------- -------------------- -------------------- --------------------
 A19101.DAY_650.PlasmacDNA.P4E9 -------------------- -------------------- -------------------- -------------------- --------------------
 A19101.DAY_650.PlasmacDNA.P4F6 -------------------- -------------------- -------------------- -------------------- M-------------------
 A19101.DAY_650.PlasmacDNA.P4F7 -------------------- -------------------- -------------------- -------------------- --------------------
A19101.DAY_650.PlasmacDNA.P4F10 -------------------- -------------------- -------------------- --------------I----- --------------------
A19101.DAY_650.PlasmacDNA.P4F12 -------------------- -------------------- -------------------- -------------------- --------------------
 A19101.DAY_650.PlasmacDNA.P4G3 -------------------- -------------------- -------------------- -------------------- --------------------
A19101.DAY_650.PlasmacDNA.P4G22 -------------------- -------------------- -------------------- -------------------- --------------------
A19101.DAY_650.PlasmacDNA.P4H10 -------------------- -------------------- -------------------- -------------------- --------------------

                    SHIV1157 SPLSFQTHLPLPRGADRPEG IEEEGGERDRDRSIRLVTGS LALIWDDLRSLCLFSYHRLR DLLLIVTRTVELLGRRGWEA LKYWWNLLLYWSQELKNSAV  800
 A19101.DAY_650.PlasmacDNA.P3M9 -------------------- -------------------- -T------------------ -------------------- --------------------
A19101.DAY_650.PlasmacDNA.P3M17 -------------------- -------------------- -------------------- -------------------- --------------------
A19101.DAY_650.PlasmacDNA.P3M24 -------------------- -------------------- -------------------- -------------------- --------------------
A19101.DAY_650.PlasmacDNA.P3N10 -------------------- -------------------- -------------------- -------------------- --------------------
A19101.DAY_650.PlasmacDNA.P3N14 -------------------- -------------------- -------------------- -------------------- --------------------
 A19101.DAY_650.PlasmacDNA.P4A5 -------------------- -------------------- -------------------- -------------------- -----------N--------
A19101.DAY_650.PlasmacDNA.P4A11 -------------------- -------------------- -------------------- -------------------- --------------------
A19101.DAY_650.PlasmacDNA.P4A14 -------------------- -------------------- -------------------- -------------------- --------------------
 A19101.DAY_650.PlasmacDNA.P4B2 -------------------- -------------------- -------------------- -------------------- --------------------
 A19101.DAY_650.PlasmacDNA.P4B8 -------------------- -------------------- -T------------------ -------------------- --------------------
A19101.DAY_650.PlasmacDNA.P4B10 -------------------- -------------------- -------------------- -------------------- --------------------
A19101.DAY_650.PlasmacDNA.P4B22 -------------------- -------------------- -------------------- -------------------- --------------------
 A19101.DAY_650.PlasmacDNA.P4C3 -------------------- -------------------- -------------------- -------------------- --------------------
 A19101.DAY_650.PlasmacDNA.P4C5 -------------------- -------------------- -------------------- -------------------- --------------------
 A19101.DAY_650.PlasmacDNA.P4C6 -------------------- -------------------- -------------------- -------------------- --------------------
A19101.DAY_650.PlasmacDNA.P4C21 -------------------- -------------------- -------------------- -------------------- --------------------
 A19101.DAY_650.PlasmacDNA.P4D3 -------------------- -------------------- -T------------------ -------------------- --------------------
 A19101.DAY_650.PlasmacDNA.P4D5 -------------------- -------------------- -------------------- -------------------- --------------------
A19101.DAY_650.PlasmacDNA.P4D15 -------------------- -------------------- -------------------- -------------------- --------------------
 A19101.DAY_650.PlasmacDNA.P4E2 -------------------- -------------------- -------------------- -------------------- --------------------
 A19101.DAY_650.PlasmacDNA.P4E8 -------------------- -------------------- -------------------- -------------------- --------------------
 A19101.DAY_650.PlasmacDNA.P4E9 -------------------- -------------------- -------------------- -------------------- --------------------
 A19101.DAY_650.PlasmacDNA.P4F6 -------------------- -------------------- -------------------- -------------------- --------------------
 A19101.DAY_650.PlasmacDNA.P4F7 -------------------- -------------------- -------------------- -------------------- --------------------
A19101.DAY_650.PlasmacDNA.P4F10 -------------------- -------------------- -------------------- -------------------- --------------------
A19101.DAY_650.PlasmacDNA.P4F12 -------------------- -------------------- -T------------------ -------------------- --------------------
 A19101.DAY_650.PlasmacDNA.P4G3 -------------------- -------------------- -------------------- -------------------- --------------------
A19101.DAY_650.PlasmacDNA.P4G22 -------------------- -------------------- -------------------- -------------------- --------------------
A19101.DAY_650.PlasmacDNA.P4H10 -------------------- -------------------- -------------------- -------------------- --------------------

                    SHIV1157 SLLNATAIAVRQYGWSYFHE AVQAVWRSATETLAGAWGDL WEILRRGGRWILAIPRRIRQ GLELTLL*  868
 A19101.DAY_650.PlasmacDNA.P3M9 -------------------- ------------------E- --T----------------- --------
A19101.DAY_650.PlasmacDNA.P3M17 -------------------- -------------------- --T----------------- --------
A19101.DAY_650.PlasmacDNA.P3M24 -------------------- -------------------- --T----------------- --------
A19101.DAY_650.PlasmacDNA.P3N10 -------------------- -------------------- --T----------------- --------
A19101.DAY_650.PlasmacDNA.P3N14 -------------------- -------------------- --T----------------- --------
 A19101.DAY_650.PlasmacDNA.P4A5 -------------------- -----------------R-- --T----------------- --------
A19101.DAY_650.PlasmacDNA.P4A11 -------------------- -------------------- --T----------------- --------
A19101.DAY_650.PlasmacDNA.P4A14 -------------------- ------------------E- --T----------------- --------
 A19101.DAY_650.PlasmacDNA.P4B2 -------------------- -------------------- --T----------------- --------
 A19101.DAY_650.PlasmacDNA.P4B8 -------------------- -------------------- --T----------------- --------
A19101.DAY_650.PlasmacDNA.P4B10 -------------------- -------------------- --T----------------- --------
A19101.DAY_650.PlasmacDNA.P4B22 -------------------- -------------------- --T----------------- --------
 A19101.DAY_650.PlasmacDNA.P4C3 -------------------- -------------------- --T----------------- --------
 A19101.DAY_650.PlasmacDNA.P4C5 -------------------- -------------------- --T----------------- --------
 A19101.DAY_650.PlasmacDNA.P4C6 -------------------- -------------------- --T----------------- --------
A19101.DAY_650.PlasmacDNA.P4C21 -------------------- -------------------- --T----------------- --------
 A19101.DAY_650.PlasmacDNA.P4D3 -------------------- -------------------- --T----------------- --------
 A19101.DAY_650.PlasmacDNA.P4D5 -------------------- ----------------R--- --T----------------- --------
A19101.DAY_650.PlasmacDNA.P4D15 -------------------- -------------------- --T----------------- --------
 A19101.DAY_650.PlasmacDNA.P4E2 -------------------- -------------------- --T----------------- --------
 A19101.DAY_650.PlasmacDNA.P4E8 -------------------- -------------------- --T----------------- --------
 A19101.DAY_650.PlasmacDNA.P4E9 -------------------- -------------------- --T----------------- --------
 A19101.DAY_650.PlasmacDNA.P4F6 -------------------- -------------------- --T----------------- --------
 A19101.DAY_650.PlasmacDNA.P4F7 -------------------- -------------------- --T----------------- --------
A19101.DAY_650.PlasmacDNA.P4F10 -------------------- -------------------- --T----------------- --------
A19101.DAY_650.PlasmacDNA.P4F12 -------------------- -------------------- --T----------------- --------
 A19101.DAY_650.PlasmacDNA.P4G3 -------------------- -------------------- --T----------------- --------
A19101.DAY_650.PlasmacDNA.P4G22 -------------------- -------------------- --T----------------- --------
A19101.DAY_650.PlasmacDNA.P4H10 -------------------- ------------------N- --T----------------- --------
```

A19102 eCD4-Ig+Ciapavir

```
                    SHIV1157 MRVKEKYQHLWRWGWRWGIM LLGMLMICSASEKLWVTVYY GVPVWKEAKTTLFCASNAKA YEKEVHNIWATHACVPTDPN PQEIVLGNVTENFNMWKNDM  100
A19102.DAY_574.PlasmacDNA.P5I18 -------------------- --------G----------- -------------------- -------------------- --------------------
A19102.DAY_574.PlasmacDNA.P5J23 -------------------- -------------------- -------------------- -------------------- --------------------
 A19102.DAY_574.PlasmacDNA.P5K3 -------------------- -------------------- -------------------- -------------------- --------------------
 A19102.DAY_574.PlasmacDNA.P5L2 -------------------- ---------T---------- -------------------- -------------------- --------------------
 A19102.DAY_574.PlasmacDNA.P5M7 -------------------- -------------------- -------------------- -------------------- --------------------
 A19102.DAY_574.PlasmacDNA.P5N1 -------------------- -------------------- -------------------- -------------------- --------------------
A19102.DAY_574.PlasmacDNA.P5N22 -------------------- -------------------- -------------------- -------------------- --------------------
 A19102.DAY_574.PlasmacDNA.P5O8 ---E---------------- -------------------- -------------------- -------------------- --------------------
A19102.DAY_574.PlasmacDNA.P5O11 ------------G------- -------------------- -------------------- -------------------- --------------------
A19102.DAY_574.PlasmacDNA.P5O20 -------------------- -------------------- -------------------- -------------------- --------------------
```

```
A19102.DAY_574.PlasmacDNA.P5O24 -------------------- -------------------- -------------------- -------------------- --------------------
 A19102.DAY_574.PlasmacDNA.P5P8 -------------------- -------------------- -------------------- -------------------- --------------------
A19102.DAY_574.PlasmacDNA.P5P10 -------------------- -------------------- -------------------- -------------------- --------------------
A19102.DAY_574.PlasmacDNA.P5P14 -------------------- -------------------- -------------------- -------------------- --------------------
A19102.DAY_574.PlasmacDNA.P5P21 -------------------- -------------------- -------------------- -------------------- --------------------
A19102.DAY_574.PlasmacDNA.P5P22 -------------------- -------------------- -------------------- -------------------- --------------------
 A19102.DAY_686.PlasmacDNA.P3P9 -------------------- -------------------- -------------------- -------------------- --------------------

                       SHIV1157 VDQMHEDIISLWDQSLKPCV KLTSLCVTLKCSNFTGKSNV TYKGDMEVKNCSFNVTTEIR DKKQKVYALFYRLDITPLDD NSSEYILINCNSSTITQACP  200
A19102.DAY_574.PlasmacDNA.P5I18 -------------------- -------------------- -------------------- -------------------- -------T------------
A19102.DAY_574.PlasmacDNA.P5J23 -------------------- -------------------- -------------------- -------------------- -------T------------
 A19102.DAY_574.PlasmacDNA.P5K3 -------------------- -------------------- -------------------- -------------------- -------T------------
 A19102.DAY_574.PlasmacDNA.P5L2 -------------------- -------------------- -------------------- -------------------- -------T------------
 A19102.DAY_574.PlasmacDNA.P5M7 -------------------- -------------------- -------------------- -------------------- -------T------------
 A19102.DAY_574.PlasmacDNA.P5N1 -------------------- -------------------- -------------------- -------------------- -------T------------
A19102.DAY_574.PlasmacDNA.P5N22 -------------------- -------------------- -------------------- -------------------- -------T------------
 A19102.DAY_574.PlasmacDNA.P5O8 -------------------- -------------------- -------------------- -------------------- -------T------------
A19102.DAY_574.PlasmacDNA.P5O11 -------------------- -------------------- -------------------- -------------------- -------T------------
A19102.DAY_574.PlasmacDNA.P5O20 -------------------- -------------------- -------------------- -------------------- -------T------------
A19102.DAY_574.PlasmacDNA.P5O24 -------------------- -------------------- -------------------- -------------------- -------T------------
 A19102.DAY_574.PlasmacDNA.P5P8 -------------------- -------------------- -------------------- -------------------- -------T------------
A19102.DAY_574.PlasmacDNA.P5P10 -------------------- -------------------- -------------------- -------------------- -------T------------
A19102.DAY_574.PlasmacDNA.P5P14 -------------------- -------------------- -------------------- -------------------- -------T------------
A19102.DAY_574.PlasmacDNA.P5P21 -------------------- -------------------- -------------------- -------------------- -------T------------
A19102.DAY_574.PlasmacDNA.P5P22 -------------------- -------------------- -------------------- -------------------- -------T------------
 A19102.DAY_686.PlasmacDNA.P3P9 -------------------- -------------------- -------I------------ -------------------- --------------------

                       SHIV1157 KVNFDPIPIHYCAPAGYAIL KCNNKTFNGTGPCHNVSTVQ CTHGIKPVVSTQLLLNGSLA EGEIIIRSENLTDNVKTIIV HFNESVEITCTRPNNNTRKS  300
A19102.DAY_574.PlasmacDNA.P5I18 -------------------- -------------------- -------------------- -------------------- --------------------
A19102.DAY_574.PlasmacDNA.P5J23 -------------------- -------------------- -------------------- -------------------- --------------------
 A19102.DAY_574.PlasmacDNA.P5K3 -------------------- -------------------- -------------------- -------------------- --------------------
 A19102.DAY_574.PlasmacDNA.P5L2 -------------------- -------------------- -------------------- -------------------- --------------------
 A19102.DAY_574.PlasmacDNA.P5M7 -------------------- -------------------- -------------------- -------------------- --------------------
 A19102.DAY_574.PlasmacDNA.P5N1 -------------------- -------------------- -------------------- -------------------- --------------------
A19102.DAY_574.PlasmacDNA.P5N22 -------------------- -------------------- -------------------- -------------------- --------------------
 A19102.DAY_574.PlasmacDNA.P5O8 -------------------- -------------------- -------------------- -------------------- --------------------
A19102.DAY_574.PlasmacDNA.P5O11 -------------------- -------------------- -------------------- -------------------- --------------------
A19102.DAY_574.PlasmacDNA.P5O20 -------------------- -------------------- -------------------- -------------------- --------------------
A19102.DAY_574.PlasmacDNA.P5O24 -------------------- -------------------- -------------------- -------------------- --------------------
 A19102.DAY_574.PlasmacDNA.P5P8 -------------------- -------------------- -------------------- -------------------- --------------------
A19102.DAY_574.PlasmacDNA.P5P10 -------------------- -------------------- -------------------- -------------------- --------------------
A19102.DAY_574.PlasmacDNA.P5P14 -------------------- -------------------- -------------------- -------------------- --------------------
A19102.DAY_574.PlasmacDNA.P5P21 -------------------- -------------------- -------------------- -------------------- --------------------
A19102.DAY_574.PlasmacDNA.P5P22 -------------------- -------------------- -------------------- -------------------- --------------------
 A19102.DAY_686.PlasmacDNA.P3P9 -------------------- -------------------- -------------------- -------------------- --------------------

                       SHIV1157 ISIGPGQAIYATGDIIGDIR QAHCNISKENWNKTLQWVRG KLKEHFPNKTIVFKPSSGGD LEITTHSFNCRGEFFYCNTS KLFNSTDNSTHMGTENNTII  400
A19102.DAY_574.PlasmacDNA.P5I18 -------------------- -------------------- -------------------- -------------------- --------------------
A19102.DAY_574.PlasmacDNA.P5J23 -------------------- -------------------- -------------------- -------------------- --------------------
 A19102.DAY_574.PlasmacDNA.P5K3 -------------------- -------------------- -------------------- -------------------- --------------------
 A19102.DAY_574.PlasmacDNA.P5L2 -------------------- -------------------- -------------------- -------------------- --------------------
 A19102.DAY_574.PlasmacDNA.P5M7 -------------------- -------------------- -------------------- -------------------- ----------Y---------
 A19102.DAY_574.PlasmacDNA.P5N1 -------------------- -------------------- -------------------- -------------------- --------------------
A19102.DAY_574.PlasmacDNA.P5N22 -------------------- -------------------- -------------------- -------------------- --------------------
 A19102.DAY_574.PlasmacDNA.P5O8 -------------------- -------------------- -------------------- -------------------- --------------------
A19102.DAY_574.PlasmacDNA.P5O11 -------------------- -------------------- -------------------- -------------------- --------------------
A19102.DAY_574.PlasmacDNA.P5O20 -------------------- -------------------- -------------------- -------------------- --------------------
A19102.DAY_574.PlasmacDNA.P5O24 -------------------- -------------------- -------------------- -------------------- --------------------
 A19102.DAY_574.PlasmacDNA.P5P8 -------------------- -------------------- -------------------- -------------------- --------------------
A19102.DAY_574.PlasmacDNA.P5P10 -------------------- -------------------- -------------------- -------------------- --------------------
A19102.DAY_574.PlasmacDNA.P5P14 -------------------- -------------------- -------------------- -------------------- --------------------
A19102.DAY_574.PlasmacDNA.P5P21 -------------------- -------------------- -------------------- -------------------- --------------------
A19102.DAY_574.PlasmacDNA.P5P22 -------------------- -------------------- -------------------- -------------------- --------------------
 A19102.DAY_686.PlasmacDNA.P3P9 -------------------- -------------------- -------------------- -------------------- --------------------

                       SHIV1157 TIPCRIKQIINMWQEVGRAM YAPPIEGNITCKSNITGLLL VRDGGWDNSTNDTETFRPGG GDMRDNWRSELYKYKVVEVK PLGIAPTKAKRRVVEREKRA  500
A19102.DAY_574.PlasmacDNA.P5I18 -------------------- -------------------- -------------------- -------------------- --------------------
A19102.DAY_574.PlasmacDNA.P5J23 ----------------E--- -------------------- -------------------- -------------------- --------------------
 A19102.DAY_574.PlasmacDNA.P5K3 -------------------- -------------------- -------------------- -------------------- --------------------
 A19102.DAY_574.PlasmacDNA.P5L2 -------------------- -------------------- -------------------- -------------------- --------------------
 A19102.DAY_574.PlasmacDNA.P5M7 -------------------- -------------------- -------------------- -------------------- --------------------
 A19102.DAY_574.PlasmacDNA.P5N1 -------------------- -------------------- -------------------- -------------------- --------------------
A19102.DAY_574.PlasmacDNA.P5N22 -------------------- -------------------- -------------------- -------------------- --------------------
 A19102.DAY_574.PlasmacDNA.P5O8 -------------------- -------------------- -------------------- -------------------- --------------------
A19102.DAY_574.PlasmacDNA.P5O11 -------------------- -------------------- -------------------- -------------------- --------------------
A19102.DAY_574.PlasmacDNA.P5O20 -------------------- -------------------- -------------------- -------------------- --------------------
A19102.DAY_574.PlasmacDNA.P5O24 -------------------- -------------------- -------------------- -------------------- --------------------
 A19102.DAY_574.PlasmacDNA.P5P8 -------------------- -------------------- -------------------- -------------------- --------------------
A19102.DAY_574.PlasmacDNA.P5P10 -------------------- -------------------- -------------------- -------------------- --------------------
A19102.DAY_574.PlasmacDNA.P5P14 -------------------- -------------------- -------------------- -------------------- --------------------
A19102.DAY_574.PlasmacDNA.P5P21 -------------------- -------------------- -------------------- -------------------- --------------------
A19102.DAY_574.PlasmacDNA.P5P22 -------------------- -------------------- -------------------- -------------------- --------------------
 A19102.DAY_686.PlasmacDNA.P3P9 -------------------- -------------------- -------------------- -------------------- --------------------

                       SHIV1157 VGIGAVFLGFLGAAGSTMGA ASITLTVQARQLLSGIVQQQ DNLLRAIEAQQHMLQLTVWG IKQLQARVLAIERYLQDQQL LGIWGCSGKLICTTAVPWND  600
A19102.DAY_574.PlasmacDNA.P5I18 -------------------- -------------------- -------------------- -------------------- --------------------
A19102.DAY_574.PlasmacDNA.P5J23 -------------------- -------------------- -------------------- -------------------- --------------------
 A19102.DAY_574.PlasmacDNA.P5K3 -------------------- -------------------- -------------------- -------------------- --------------------
 A19102.DAY_574.PlasmacDNA.P5L2 -------------------- -------------------- -------------------- -------------------- --------------------
 A19102.DAY_574.PlasmacDNA.P5M7 -------------------- -------------------- -------------------- -------------------- --------------------
 A19102.DAY_574.PlasmacDNA.P5N1 -------------------- -------------------- -------------------- -------------------- --------------T-----
A19102.DAY_574.PlasmacDNA.P5N22 -------------------- -------------------- -------------------- -------------------- --------------------
 A19102.DAY_574.PlasmacDNA.P5O8 -------------------- -------------------- -------------------- -------------------- --------------------
A19102.DAY_574.PlasmacDNA.P5O11 -------------------- -------------------- -------------------- -------------------- --------------------
A19102.DAY_574.PlasmacDNA.P5O20 -------------------- -------------------- -------------------- -------------------- --------------------
A19102.DAY_574.PlasmacDNA.P5O24 -------------------- -------------------- -------------------- -------------------- --------------------
 A19102.DAY_574.PlasmacDNA.P5P8 -------------------- -------------------- -------------------- -------------------- --------------------
A19102.DAY_574.PlasmacDNA.P5P10 -------------------- -------------------- -------------------- -------------------- --------------------
A19102.DAY_574.PlasmacDNA.P5P14 -------------------- -------------------- -------------------- -------------------- --------------------
A19102.DAY_574.PlasmacDNA.P5P21 -------------------- -------------------- -------------------- -------------------- --------------------
A19102.DAY_574.PlasmacDNA.P5P22 -------------------- -------------------- -------------------- -------------------- --------------------
 A19102.DAY_686.PlasmacDNA.P3P9 -------------------- -------------------- -------------------- -------------------- --------------------

                       SHIV1157 SWSNKSQTDIWENMTWMQWD REISRHTDTIYRLLEDSQNQ QEKNEKDLLALDSWKNLWNW FSITRWLWYIKIFIMIVGGL IGLRIIFAVLSIVNRVRQGY  700
A19102.DAY_574.PlasmacDNA.P5I18 -------------------- -------------------- ----K--------------- -------------------- --------------------
```

```
A19102.DAY_574.PlasmacDNA.P5J23 -------------------- -------------------- -------------------- -------------------- --------------------
 A19102.DAY_574.PlasmacDNA.P5K3 -------------------- -------------------- -------------------- -------------------- --------------------
 A19102.DAY_574.PlasmacDNA.P5L2 -------------------- -------------------- -------------------- -------------------- --------------------
 A19102.DAY_574.PlasmacDNA.P5M7 -------------------- -------------------- -------------------- -------------------- --------------------
 A19102.DAY_574.PlasmacDNA.P5N1 -------------------- -------------------- -------------------- -------------------- --------------------
A19102.DAY_574.PlasmacDNA.P5N22 -------------------- -------------------- -------------------- -------------------- --------------------
 A19102.DAY_574.PlasmacDNA.P5O8 -------------------- -------------------- -------------------- -------------------- --------------------
A19102.DAY_574.PlasmacDNA.P5O11 -------------------- -------------------- -------------------- -------------------- --------------------
A19102.DAY_574.PlasmacDNA.P5O20 -------------------- -------------------- -------------------- -------------------- --------------------
A19102.DAY_574.PlasmacDNA.P5O24 -------------------- -------------------- -------------------- -------------------- --------------------
 A19102.DAY_574.PlasmacDNA.P5P8 -------------------- -------------------- -------------------- -------------------- --------------------
A19102.DAY_574.PlasmacDNA.P5P10 -------------------- -------------------- -------------------- -------------------- --------------------
A19102.DAY_574.PlasmacDNA.P5P14 -------------------- -------------------- -------------------- -------------------- --------------------
A19102.DAY_574.PlasmacDNA.P5P21 -------------------- -------------------- -------------------- -------------------- --------------------
A19102.DAY_574.PlasmacDNA.P5P22 -------------------- -------------------- -------------------- -------------------- --------------------
 A19102.DAY_686.PlasmacDNA.P3P9 -------------------- -------------------- -------------------- -------------------- --------------------

                       SHIV1157 SPLSFQTHLPLPRGADRPEG IEEEGGERDRDRSIRLVTGS LALIWDDLRSLCLFSYHRLR DLLLIVTRTVELLGRRGWEA LKYWWNLLLYWSQELKNSAV  800
A19102.DAY_574.PlasmacDNA.P5I18 -------------------- -------------------- -------------------- -------------------- --------------------
A19102.DAY_574.PlasmacDNA.P5J23 -------------------- -------------------- -------------------- -------------------- --------------------
 A19102.DAY_574.PlasmacDNA.P5K3 -------------------- -------------------- -------------------- -------------------- --------------------
 A19102.DAY_574.PlasmacDNA.P5L2 -------------------- -------------------- -------------------- -------------------- --------------------
 A19102.DAY_574.PlasmacDNA.P5M7 -------------------- -------------------- -------------------- -------------------- --------------------
 A19102.DAY_574.PlasmacDNA.P5N1 -------------------- -------------------- -------------------- -------------------- --------------------
A19102.DAY_574.PlasmacDNA.P5N22 -------------------- -------------------- -------------------- -------------------- --------------------
 A19102.DAY_574.PlasmacDNA.P5O8 -------------------- -------------------- -------------------- -------------------- --------------------
A19102.DAY_574.PlasmacDNA.P5O11 -------------------- -------------------- -------------------- -------------------- --------------------
A19102.DAY_574.PlasmacDNA.P5O20 -------------------- -------------------- -------------------- -------------------- --------------------
A19102.DAY_574.PlasmacDNA.P5O24 -------------------- -------------------- -------------------- -------------------- --------------------
 A19102.DAY_574.PlasmacDNA.P5P8 -------------------- -------------------- -------------------- -------------------- --------------------
A19102.DAY_574.PlasmacDNA.P5P10 -------------------- -------------------- -------------------- -------------------- --------------------
A19102.DAY_574.PlasmacDNA.P5P14 -------------------- -------------------- -------------------- -------------------- --------------------
A19102.DAY_574.PlasmacDNA.P5P21 -------------------- -------------------- -------------------- -------------------- --------------------
A19102.DAY_574.PlasmacDNA.P5P22 -------------------- -------------------- -------------------- -------------------- --------------------
 A19102.DAY_686.PlasmacDNA.P3P9 -------------------- -------------------- -------------------- -------------------- --------------------

                       SHIV1157 SLLNATAIAVRQYGWSYFHE AVQAVWRSATETLAGAWGDL WEILRRGGRWILAIPRRIRQ GLELTLL*  868
A19102.DAY_574.PlasmacDNA.P5I18 -------------------- ------K------------- -------------------- --------
A19102.DAY_574.PlasmacDNA.P5J23 -------------------- ------K------------- -------------------- --------
 A19102.DAY_574.PlasmacDNA.P5K3 -------------------- ------K------------- -------------------- --------
 A19102.DAY_574.PlasmacDNA.P5L2 -------------------- ------K------------- -------------------- --------
 A19102.DAY_574.PlasmacDNA.P5M7 -------------------- ------K------------- -------------------- --------
 A19102.DAY_574.PlasmacDNA.P5N1 -------------------- ------K------------- -------------------- --------
A19102.DAY_574.PlasmacDNA.P5N22 -------------------- ------K------------- -------------------- --------
 A19102.DAY_574.PlasmacDNA.P5O8 -------------------- ------K------------- -------------------- --------
A19102.DAY_574.PlasmacDNA.P5O11 -------------------- -A----K--------T---- -------------------- --------
A19102.DAY_574.PlasmacDNA.P5O20 -------------------- ------K------------- -------------------- --------
A19102.DAY_574.PlasmacDNA.P5O24 -------------------- ------K------------- -------------------- --------
 A19102.DAY_574.PlasmacDNA.P5P8 -------------------- ------K------------- -------------------- --------
A19102.DAY_574.PlasmacDNA.P5P10 -------------------- ------K------------- -------------------- --------
A19102.DAY_574.PlasmacDNA.P5P14 -----------------L-- ------K------------- -------------------- --------
A19102.DAY_574.PlasmacDNA.P5P21 -------------------- ------K------------- -------------------- --------
A19102.DAY_574.PlasmacDNA.P5P22 -------------------- ------K------------- -------------------- --------
 A19102.DAY_686.PlasmacDNA.P3P9 -------------------- ------K------------- -------------------- --------
```

A19103 Control

```
                       SHIV1157 MRVKEKYQHLWRWGWRWGIM LLGMLMICSASEKLWVTVYY GVPVWKEAKTTLFCASNAKA YEKEVHNIWATHACVPTDPN PQEIVLGNVTENFNMWKNDM  100
A19103.DAY_575.PlasmacDNA.P2M11 -------------------- -------------------- -------------------- ----E--------------- --------------------
A19103.DAY_575.PlasmacDNA.P2M13 -------------------- -------------------- -------------------- ----E--------------- --------------------
A19103.DAY_575.PlasmacDNA.P2M16 -------------------- -------------------- -------------------- ----E--------------- --------------------
A19103.DAY_575.PlasmacDNA.P2M18 -------------------- -------------------- -------------------- ----E--------------- --------------------
 A19103.DAY_575.PlasmacDNA.P2N4 -------------------- -------------------- -------------------- ----E--------------- --------------------
A19103.DAY_575.PlasmacDNA.P2N11 -------------------- -------------------- -------------------- ----E--------------- --------------------
A19103.DAY_575.PlasmacDNA.P2N19 -------------------- -------------------- -------------------- ----E--------------- --------------------
 A19103.DAY_575.PlasmacDNA.P5A9 -------------------- -------------------- -------------------- ----E--------------- --------------------
A19103.DAY_575.PlasmacDNA.P5A13 -------------------- -------------------- -------------------- ----E--------------- --------------------
A19103.DAY_575.PlasmacDNA.P5A17 -------------------- -------------------- -------------------- ----E--------------- --------------------
A19103.DAY_575.PlasmacDNA.P5A21 -------------------- -------------------- -------------------- ----E--------------- --------------------
A19103.DAY_575.PlasmacDNA.P5B14 -------------------- -------------------- -------------------- ----E--------------- --------------------
A19103.DAY_575.PlasmacDNA.P5B19 -------------------- -------------------- -------------------- ----E--------------- --------------------
 A19103.DAY_575.PlasmacDNA.P5C1 -------------------- -------------------- -------------------- ----E--------------- --------------------
 A19103.DAY_575.PlasmacDNA.P5C5 -------------------- -------------------- -------------------- ----E--------------- --------------------
 A19103.DAY_575.PlasmacDNA.P5C9 -------------------- -------------------- -------------------- ----E--------------- --------------------
A19103.DAY_575.PlasmacDNA.P5C12 -------------------- -------------------- -------------------- -------------------- --------------------
A19103.DAY_575.PlasmacDNA.P5D21 -------------------- -------------------- -------------------- ----E--------------- --------------------
 A19103.DAY_575.PlasmacDNA.P5E8 -------------------- -------------------- -------------------- -K--E--------------- --------------------
A19103.DAY_575.PlasmacDNA.P5E22 -------------------- -------------------- -------------------- -------------------- -----------D--------
A19103.DAY_575.PlasmacDNA.P5E23 -------------------- -------------------- -------------------- ----E--------------- --------------------
 A19103.DAY_575.PlasmacDNA.P5F2 -------------------- -------------------- -------------------- -----Y-------------- --------------------
 A19103.DAY_575.PlasmacDNA.P5F3 -------------------- -------------------- -------------------- ----E--------------- --------------------
A19103.DAY_575.PlasmacDNA.P5F20 -------------------- -------------------- -------------------- -------------------- --------------------
 A19103.DAY_575.PlasmacDNA.P5G3 -------------------- -------------------- -------------------- ----E--------------- --------------------
 A19103.DAY_575.PlasmacDNA.P5G4 -------------------- -------------------- -------------------- ----E--------------- --------------------
A19103.DAY_575.PlasmacDNA.P5G11 -------------------- -------------------- -------------------- ----E--------------- --------------------
A19103.DAY_650.PlasmacDNA.P3J11 -------------------- -------------------- -------------------- -------------------- --------------------
A19103.DAY_650.PlasmacDNA.P4D15 -------------------- -------------------- -------------------- -------------------- --------------------
A19103.DAY_650.PlasmacDNA.P4D20 -------------------- -------------------- -------------------- -------------------- --------------------
 A19103.DAY_650.PlasmacDNA.P4E9 -------------------- -------------------- -------------------- -------------------- --------------------
A19103.DAY_650.PlasmacDNA.P4E21 -------------------- -------------------- -------------------- -------------------- --------------------
 A19103.DAY_650.PlasmacDNA.P4F2 -------------------- -------------------- -------------------- -------------------- ---V----------------
 A19103.DAY_650.PlasmacDNA.P4F8 -------------------- -------------------- -------------------- ----E--------------- --------------------
 A19103.DAY_650.PlasmacDNA.P4F9 -------------------- -------------------- -------------------- ----E--------------- --------------------
A19103.DAY_650.PlasmacDNA.P4G23 -------------------- -------------------- -------------------- ----E--------------- --------------------

                       SHIV1157 VDQMHEDIISLWDQSLKPCV KLTSLCVTLKCSNFTGKSNV TYKGDMEVKNCSFNVTTEIR DKKQKVYALFYRLDITPLDD NSSEYILINCNSSTITQACP  200
A19103.DAY_575.PlasmacDNA.P2M11 -------------------- -------------------- -------------------- -------------------- --------------------
A19103.DAY_575.PlasmacDNA.P2M13 -------------------- -------------------- -------------------- -------------------- --------------------
A19103.DAY_575.PlasmacDNA.P2M16 -------------------- -------------------- -------------------- -------------------- --------------------
A19103.DAY_575.PlasmacDNA.P2M18 -------------------- -------------------- -------------------- -------------------- --------------------
 A19103.DAY_575.PlasmacDNA.P2N4 -------------------- -------------------- -------------------- -------------------- --------------------
A19103.DAY_575.PlasmacDNA.P2N11 -------------------- -------------------- -------------------- -------------------- --------------------
A19103.DAY_575.PlasmacDNA.P2N19 -------------------- -------------------- -------------------- -------------------- --------------------
 A19103.DAY_575.PlasmacDNA.P5A9 -------------------- -------------------- -------------------- -------------------- --------------------
A19103.DAY_575.PlasmacDNA.P5A13 -------------------- -------------------- -------------------- -------------------- --------------------
A19103.DAY_575.PlasmacDNA.P5A17 -------------------- -------------------- -------------------- -------------------- --------------------
```

```
A19103.DAY_575.PlasmacDNA.P5A21 -------------------- -------------------- -------------------- -------------------- --------------------
A19103.DAY_575.PlasmacDNA.P5B14 -------------------- -------------------- -------------------- -------------------- --------------------
A19103.DAY_575.PlasmacDNA.P5B19 -------------------- -------------------- -------------------- -------------------- --------------------
 A19103.DAY_575.PlasmacDNA.P5C1 -------------------- -------------------- -------------------- -------------------- --------------------
 A19103.DAY_575.PlasmacDNA.P5C5 -------------------- -------------------- -------------------- -------------------- --------------------
 A19103.DAY_575.PlasmacDNA.P5C9 -------------------- -------------------- -------------------- -------------------- --------------------
A19103.DAY_575.PlasmacDNA.P5C12 -------------------- -------------------- -------------------- -------------------- --------------------
A19103.DAY_575.PlasmacDNA.P5D21 -------------------- -------------------- -------------------- -------------------- --------------------
 A19103.DAY_575.PlasmacDNA.P5E8 -------------------- -------------------- -------------------- -------------------- --------------------
A19103.DAY_575.PlasmacDNA.P5E22 -------------------- -------------------- -------------------- -------------------- --------------------
A19103.DAY_575.PlasmacDNA.P5E23 -------------------- -------------------- -------------------- -------------------- --------------------
 A19103.DAY_575.PlasmacDNA.P5F2 -------------------- -------------------- -------------------- -------------------- --------------------
 A19103.DAY_575.PlasmacDNA.P5F3 -------------------- -------------------- -------------------- -------------------- --------------------
A19103.DAY_575.PlasmacDNA.P5F20 -------------------- -------------------- -------------------- -------------------- --------------------
 A19103.DAY_575.PlasmacDNA.P5G3 -------------------- -------------------- -------------------- -------------------- --------------------
 A19103.DAY_575.PlasmacDNA.P5G4 -------------------- -------------------- -------------------- -------------------- --------------------
A19103.DAY_575.PlasmacDNA.P5G11 -------------------- -------------------- -------------------- -------------------- --------------------
A19103.DAY_650.PlasmacDNA.P3J11 -------------------- -------------------- -------------------- -------------------- --------------------
A19103.DAY_650.PlasmacDNA.P4D15 -------------------- -------------------- -------------------- -------------------- --------------------
A19103.DAY_650.PlasmacDNA.P4D20 -------------------- -------------------- -------------------- -------------------- --------------------
 A19103.DAY_650.PlasmacDNA.P4E9 -------------------- -------------------- -------------------- -------------------- --------------------
A19103.DAY_650.PlasmacDNA.P4E21 -------------------- -------------------- -------------------- -------------------- --------------------
 A19103.DAY_650.PlasmacDNA.P4F2 -------------------- -------------------- -------------------- -------------------- --------------------
 A19103.DAY_650.PlasmacDNA.P4F8 -------------------- -------------------- -------------------- -------------------- --------------------
 A19103.DAY_650.PlasmacDNA.P4F9 -------------------- -------------------- -------------------- -------------------- --------------------
A19103.DAY_650.PlasmacDNA.P4G23 -------------------- -------------------- -------------------- -------------------- --------------------

                       SHIV1157 KVNFDPIPIHYCAPAGYAIL KCNNKTFNGTGPCHNVSTVQ CTHGIKPVVSTQLLLNGSLA EGEIIIRSENLTDNVKTIIV HFNESVEITCTRPNNNTRKS  300
A19103.DAY_575.PlasmacDNA.P2M11 -------------------- -------------------- -------------------- -------------------- --------------------
A19103.DAY_575.PlasmacDNA.P2M13 -------------------- -------------------- -------------------- -------------------- --------------------
A19103.DAY_575.PlasmacDNA.P2M16 -------------------- -------------------- -------------------- -------------------- --------------------
A19103.DAY_575.PlasmacDNA.P2M18 -------------------- -------------------- -------------------- -------------------- --------------------
 A19103.DAY_575.PlasmacDNA.P2N4 -------------------- -------------------- -------------------- -------------------- --------------------
A19103.DAY_575.PlasmacDNA.P2N11 -------------------- -------------------- -------------------- -------------------- --------------------
A19103.DAY_575.PlasmacDNA.P2N19 -------------------- -------------------- -------------------- -------------------- --------------------
 A19103.DAY_575.PlasmacDNA.P5A9 -------------------- -------------------- -------------------- -------------------- --------------------
A19103.DAY_575.PlasmacDNA.P5A13 -------------------- -------------------- -------------------- -------------------- --------------------
A19103.DAY_575.PlasmacDNA.P5A17 -------------------- -------------------- -------------------- -------------------- --------------------
A19103.DAY_575.PlasmacDNA.P5A21 -------------------- -------------------- -------------------- -------------------- --------------------
A19103.DAY_575.PlasmacDNA.P5B14 -------------------- -------------------- -------------------- -------------------- --------------------
A19103.DAY_575.PlasmacDNA.P5B19 -------------------- -------------------- -------------------- -------------------- --------------------
 A19103.DAY_575.PlasmacDNA.P5C1 -------------------- -------------------- -------------------- K------------------- --------------------
 A19103.DAY_575.PlasmacDNA.P5C5 -------------------- -------------------- -------------------- -------------------- --------------------
 A19103.DAY_575.PlasmacDNA.P5C9 -------------------- -------------------- -------------------- -------------------- --------------------
A19103.DAY_575.PlasmacDNA.P5C12 -------------------- -------------------- -------------------- -------------------- --------------------
A19103.DAY_575.PlasmacDNA.P5D21 -------------------- -------------------- -------------------- -------------------- --------------------
 A19103.DAY_575.PlasmacDNA.P5E8 -------------------- -------------------- -------------------- -------------------- --------------------
A19103.DAY_575.PlasmacDNA.P5E22 -------------------- -------------------- -------------------- -------------------- --------------------
A19103.DAY_575.PlasmacDNA.P5E23 -------------------- -------------------- -------------------- -------------------- --------------------
 A19103.DAY_575.PlasmacDNA.P5F2 -------------------- -------------------- -------------------- -------------------- --------------------
 A19103.DAY_575.PlasmacDNA.P5F3 -------------------- -------------------- -------------------- -------------------- --------------------
A19103.DAY_575.PlasmacDNA.P5F20 -------------------- -------------------- -------------------- -------------------- --------------------
 A19103.DAY_575.PlasmacDNA.P5G3 -------------------- -------------------- -------------------- -------------------- --------------------
 A19103.DAY_575.PlasmacDNA.P5G4 -------------------- -------------------- -------------------- -------------------- --------------------
A19103.DAY_575.PlasmacDNA.P5G11 -------------------- -------------------- -------------------- -------------------- --------------------
A19103.DAY_650.PlasmacDNA.P3J11 -------------------- -------------------- -------------------- -------------------- --------------------
A19103.DAY_650.PlasmacDNA.P4D15 -------------------- -------------------- -------------------- -------------------- --------------------
A19103.DAY_650.PlasmacDNA.P4D20 -------------------- -------------------- -------------------- -------------------- --------------------
 A19103.DAY_650.PlasmacDNA.P4E9 -------------------- -------------------- -------------------- -------------------- --------------------
A19103.DAY_650.PlasmacDNA.P4E21 -------------------- -------------------- -------------------- -------------------- --------------------
 A19103.DAY_650.PlasmacDNA.P4F2 -------------------- -------------------- -------------------- -------------------- --------------------
 A19103.DAY_650.PlasmacDNA.P4F8 -------------------- -------------------- -------------------- -------------------- --------------------
 A19103.DAY_650.PlasmacDNA.P4F9 -------------------- -------------------- -------------------- -------------------- --------------------
A19103.DAY_650.PlasmacDNA.P4G23 -------------------- -------------------- -------------------- -------------------- --------------------

                       SHIV1157 ISIGPGQAIYATGDIIGDIR QAHCNISKENWNKTLQWVRG KLKEHFPNKTIVFKPSSGGD LEITTHSFNCRGEFFYCNTS KLFNSTDNSTHMGTENNTII  400
A19103.DAY_575.PlasmacDNA.P2M11 -------------------- -------------------- -------------------- -------------------- --------------------
A19103.DAY_575.PlasmacDNA.P2M13 -------------------- -------------------- -------------------- -------------------- --------------------
A19103.DAY_575.PlasmacDNA.P2M16 -------------------- -------------------- -------------------- -------------------- --------------------
A19103.DAY_575.PlasmacDNA.P2M18 -------------------- -------------------- -------------------- -------------------- --------------------
 A19103.DAY_575.PlasmacDNA.P2N4 -------------------- -------------------- -------------------- -------------------- --------------------
A19103.DAY_575.PlasmacDNA.P2N11 -------------------- -------------------- -------------------- -------------------- --------------------
A19103.DAY_575.PlasmacDNA.P2N19 -------------------- -------------------- -------------------- -------------------- --------------------
 A19103.DAY_575.PlasmacDNA.P5A9 -------------------- -------------------- -------------------- -------------------- --------------------
A19103.DAY_575.PlasmacDNA.P5A13 -------------------- -------------------- -------------------- -------------------- --------------------
A19103.DAY_575.PlasmacDNA.P5A17 -------------------- -------------------- -------------------- -------------------- --------------------
A19103.DAY_575.PlasmacDNA.P5A21 -------------------- -------------------- -------------------- ----------------Y--- --------------------
A19103.DAY_575.PlasmacDNA.P5B14 -------------------- -------------------- -------------------- -------------------- --------------------
A19103.DAY_575.PlasmacDNA.P5B19 -------------------- -------------------- -------------------- -------------------- --------------------
 A19103.DAY_575.PlasmacDNA.P5C1 -------------------- -------------------- -------------------- -------------------- --------------------
 A19103.DAY_575.PlasmacDNA.P5C5 -------------------- -------------------- -------------------- -------------------- --------------------
 A19103.DAY_575.PlasmacDNA.P5C9 -------------------- -------------------- -------------------- -------------------- --------------------
A19103.DAY_575.PlasmacDNA.P5C12 -------------------- -------------------- -------------------- -------------------- --------------------
A19103.DAY_575.PlasmacDNA.P5D21 -------------------- -------------------- -------------------- -------------------- --------------------
 A19103.DAY_575.PlasmacDNA.P5E8 -------------------- -------------------- -------------------- -------------------- --------------------
A19103.DAY_575.PlasmacDNA.P5E22 -------------------- -------------------- -------------------- -------------------- --------------------
A19103.DAY_575.PlasmacDNA.P5E23 -------------------- -------------------- -------------------- -------------------- --------------------
 A19103.DAY_575.PlasmacDNA.P5F2 -------------------- -------------------- -------------------- -------------------- --------------------
 A19103.DAY_575.PlasmacDNA.P5F3 -------------------- -------------------- -------------------- -------------------- --------------------
A19103.DAY_575.PlasmacDNA.P5F20 -------------------- -------------------- -------------------- -------------------- --------------------
 A19103.DAY_575.PlasmacDNA.P5G3 -------------------- -------------------- -------------------- -------------------- --------------------
 A19103.DAY_575.PlasmacDNA.P5G4 -------------------- -------------------- -------------------- -------------------- --------------------
A19103.DAY_575.PlasmacDNA.P5G11 -------------------- -------------------- -------------------- -------------------- --------------------
A19103.DAY_650.PlasmacDNA.P3J11 -------------------- -------------------- -------------------- ------------K------- --------------------
A19103.DAY_650.PlasmacDNA.P4D15 -------------------- -------------------- -------------------- -------------------- --------------------
A19103.DAY_650.PlasmacDNA.P4D20 -------------------- -------------------- -------------------- -------------------- --------------------
 A19103.DAY_650.PlasmacDNA.P4E9 -------------------- -------------------- -------------------- -------------------- --------------------
A19103.DAY_650.PlasmacDNA.P4E21 -------------------- -------------------- -------------------- -------------------- -----------I--------
 A19103.DAY_650.PlasmacDNA.P4F2 -------------------- -------------------- -------------------- -------------------- --------------------
 A19103.DAY_650.PlasmacDNA.P4F8 -------------------- -------------------- -------------------- -------------------- --------------------
 A19103.DAY_650.PlasmacDNA.P4F9 -------------------- -------------------- -------------------- -------------------- --------------------
A19103.DAY_650.PlasmacDNA.P4G23 -------------------- -------------------- -------------------- -------------------- --------------------

                       SHIV1157 TIPCRIKQIINMWQEVGRAM YAPPIEGNITCKSNITGLLL VRDGGWDNSTNDTETFRPGG GDMRDNWRSELYKYKVVEVK PLGIAPTKAKRRVVEREKRA  500
A19103.DAY_575.PlasmacDNA.P2M11 -------------------- -------------------- -------------------- -------------------- --------------------
```

```
A19103.DAY_575.PlasmacDNA.P2M13 -------------------- -------------------- -------------------- -------------------- --------------------
A19103.DAY_575.PlasmacDNA.P2M16 -------------------- -------------------- -------------------- -------------------- --------------G-----
A19103.DAY_575.PlasmacDNA.P2M18 -------------------- -------------------- -------------------- -------------------- --------------------
 A19103.DAY_575.PlasmacDNA.P2N4 -------------------- -------------------- -------------------- -------------------- --------------------
A19103.DAY_575.PlasmacDNA.P2N11 -------------------- -------------------- -------------------- -------------------- --------------------
A19103.DAY_575.PlasmacDNA.P2N19 -------------------- -------------------- -------------------- -------------------- --------------------
 A19103.DAY_575.PlasmacDNA.P5A9 -------------------- -------------------- -------------------- -------------------- --------------------
A19103.DAY_575.PlasmacDNA.P5A13 -------------------- -------------------- -------------------- -------------------- --------------------
A19103.DAY_575.PlasmacDNA.P5A17 -------------------- -------------------- -------------------- -------------------- --------------------
A19103.DAY_575.PlasmacDNA.P5A21 -------------------- -------------------- -------------------- -------------------- --------------------
A19103.DAY_575.PlasmacDNA.P5B14 -------------------- -------------------- -------------------- -------------------- --------------------
A19103.DAY_575.PlasmacDNA.P5B19 -------------------- -------------------- -------------------- -------------------- --------------------
 A19103.DAY_575.PlasmacDNA.P5C1 -------------------- -------------------- -------------------- -------------------- --------------------
 A19103.DAY_575.PlasmacDNA.P5C5 -------------------- -------------------- -------------------- -------------------- --------------------
 A19103.DAY_575.PlasmacDNA.P5C9 -------------------- -------------------- -------------------- -------------------- --------------------
A19103.DAY_575.PlasmacDNA.P5C12 -------------------- -------------------- -------------------- -------------------- --------------------
A19103.DAY_575.PlasmacDNA.P5D21 -------------------- -------------------- -------------------- -------------------- --------------------
 A19103.DAY_575.PlasmacDNA.P5E8 -------------------- -------------------- -------------------- -------------------- --------------------
A19103.DAY_575.PlasmacDNA.P5E22 -------------------- -V------------------ -------------------- -------------------- --------------------
A19103.DAY_575.PlasmacDNA.P5E23 -------------------- -------------------- -------------------- -------------------- --------------------
 A19103.DAY_575.PlasmacDNA.P5F2 -------------------- -------------------- -------------------- -------------------- --------------------
 A19103.DAY_575.PlasmacDNA.P5F3 -------------------- -------------------- -------------------- -------------------- --------------------
A19103.DAY_575.PlasmacDNA.P5F20 -------------------- -------------------- -------------------- -------------------- --------------------
 A19103.DAY_575.PlasmacDNA.P5G3 -------------------- -------------------- -------------------- -------------------- --------------------
 A19103.DAY_575.PlasmacDNA.P5G4 -------------------- -------------------- -------------------- -------------------- --------------------
A19103.DAY_575.PlasmacDNA.P5G11 -------------------- -------------------- -------------------- -------------------- --------------------
A19103.DAY_650.PlasmacDNA.P3J11 -------------------- -------------------- ------------------E- -------------------- --------------------
A19103.DAY_650.PlasmacDNA.P4D15 -------------------- -------------------- -------------------- -------------------- --------------------
A19103.DAY_650.PlasmacDNA.P4D20 -------------------- -------------------- -------------------- -------------------- --------------------
 A19103.DAY_650.PlasmacDNA.P4E9 -------------------- -------------------- -------------------- -------------------- --------------------
A19103.DAY_650.PlasmacDNA.P4E21 -------------------- -------------------- -------------------- -------------------- --------------------
 A19103.DAY_650.PlasmacDNA.P4F2 -------------------- -------------------- -------------------- -------------------- --------------------
 A19103.DAY_650.PlasmacDNA.P4F8 -------------------- -------------------- -------------------- -------------------- --------------------
 A19103.DAY_650.PlasmacDNA.P4F9 -------------------- -------------------- -------------------- -------------------- --------------------
A19103.DAY_650.PlasmacDNA.P4G23 -------------------- -------------------- -------------------- -------------------- --------------------

                       SHIV1157 VGIGAVFLGFLGAAGSTMGA ASITLTVQARQLLSGIVQQQ DNLLRAIEAQQHMLQLTVWG IKQLQARVLAIERYLQDQQL LGIWGCSGKLICTTAVPWND  600
A19103.DAY_575.PlasmacDNA.P2M11 -------------------- -------------------- -------------------- -------------------- --------------------
A19103.DAY_575.PlasmacDNA.P2M13 -------------------- -------------------- -------------------- -------------------- --------------------
A19103.DAY_575.PlasmacDNA.P2M16 -------------------- -------------------- -------------------- -------------------- --------------------
A19103.DAY_575.PlasmacDNA.P2M18 -------------------- -------------------- -------------------- -------------------- --------------------
 A19103.DAY_575.PlasmacDNA.P2N4 -------------------- -------------------- -------------------- -------------------- --------------------
A19103.DAY_575.PlasmacDNA.P2N11 -------------------- -------------------- -------------------- -------------------- --------------------
A19103.DAY_575.PlasmacDNA.P2N19 -------------------- -------------------- -------------------- -------------------- --------------------
 A19103.DAY_575.PlasmacDNA.P5A9 -------------------- -------------------- -------------------- -------------------- --------------------
A19103.DAY_575.PlasmacDNA.P5A13 -------------------- -------------------- -------------------- -------------------- --------------------
A19103.DAY_575.PlasmacDNA.P5A17 -------------------- -------------------- -------------------- -------------------- --------------------
A19103.DAY_575.PlasmacDNA.P5A21 -------------------- -------------------- -------------------- -------------------- --------------------
A19103.DAY_575.PlasmacDNA.P5B14 -------------------- -------------------- -------------------- -------------------- --------------------
A19103.DAY_575.PlasmacDNA.P5B19 -------------------- -------------------- -------------------- -------------------- --------------------
 A19103.DAY_575.PlasmacDNA.P5C1 -------------------- -------------------- -------------------- -------------------- --------------------
 A19103.DAY_575.PlasmacDNA.P5C5 -------------------- -------------------- -------------------- -------------------- --------------------
 A19103.DAY_575.PlasmacDNA.P5C9 -------------------- -------------------- -------------------- -------------------- --------------T-----
A19103.DAY_575.PlasmacDNA.P5C12 -------------------- -------------------- -------------------- -------------------- --------------------
A19103.DAY_575.PlasmacDNA.P5D21 -------------------- -------------------- -------------------- -------------------- --------------------
 A19103.DAY_575.PlasmacDNA.P5E8 -------------------- -------------------- -------------------- -------------------- --------------------
A19103.DAY_575.PlasmacDNA.P5E22 -------------------- -------------------- -------------------- -------------------- --------------------
A19103.DAY_575.PlasmacDNA.P5E23 -------------------- -------------------- -------------------- -------------------- --------------------
 A19103.DAY_575.PlasmacDNA.P5F2 -------------------- -------------------- -------------------- -------------------- --------------------
 A19103.DAY_575.PlasmacDNA.P5F3 -------------------- -------------------- -------------------- -------------------- --------------------
A19103.DAY_575.PlasmacDNA.P5F20 -------------------- -------------------- -------------------- -------------------- --------------------
 A19103.DAY_575.PlasmacDNA.P5G3 -------------------- -------------------- -------------------- -------------------- --------------------
 A19103.DAY_575.PlasmacDNA.P5G4 -------------------- -------------------- -------------------- -------------------- --------------------
A19103.DAY_575.PlasmacDNA.P5G11 -------------------- -------------------- -------------------- -------------------- --------------------
A19103.DAY_650.PlasmacDNA.P3J11 -------------------- -------------------- -------------------- -------I------------ --------------------
A19103.DAY_650.PlasmacDNA.P4D15 -------------------- -------------------- -------------------- -------I------------ --------------------
A19103.DAY_650.PlasmacDNA.P4D20 -------------------- -------------------- -------------------- -------------------- -------E------------
 A19103.DAY_650.PlasmacDNA.P4E9 -------------------- -------------------- -------------------- -------I------------ --------------------
A19103.DAY_650.PlasmacDNA.P4E21 -------------------- -------------------- -------------------- -------I------------ --------------------
 A19103.DAY_650.PlasmacDNA.P4F2 -------------------- -------------------- -------------------- -------I------------ --------------------
 A19103.DAY_650.PlasmacDNA.P4F8 -------------------- -------------------- -------------------- -------------------- --------------------
 A19103.DAY_650.PlasmacDNA.P4F9 -------------------- -------------------- -------------------- -------------------- --------------------
A19103.DAY_650.PlasmacDNA.P4G23 -------------------- -------------------- -------------------- -------------------- --------------------

                       SHIV1157 SWSNKSQTDIWENMTWMQWD REISRHTDTIYRLLEDSQNQ QEKNEKDLLALDSWKNLWNW FSITRWLWYIKIFIMIVGGL IGLRIIFAVLSIVNRVRQGY  700
A19103.DAY_575.PlasmacDNA.P2M11 -------------------- -------------------- -------------------- -------------------- --------------------
A19103.DAY_575.PlasmacDNA.P2M13 -------------------- -------------------- -------------------- -------------------- --------------------
A19103.DAY_575.PlasmacDNA.P2M16 -------------------- -------------------- -------------------- -------------------- --------------------
A19103.DAY_575.PlasmacDNA.P2M18 -------------------- -------------------- -------------------- -------------------- --------------------
 A19103.DAY_575.PlasmacDNA.P2N4 -------------------- -------------------- -------------------- -------------------- --------------------
A19103.DAY_575.PlasmacDNA.P2N11 -------------------- -------------------- -------------------- -------------------- --------------------
A19103.DAY_575.PlasmacDNA.P2N19 -------------------- -------------------- -------------------- -------------------- --------------------
 A19103.DAY_575.PlasmacDNA.P5A9 -------------------- -------------------- -------------------- -------------------- --------------------
A19103.DAY_575.PlasmacDNA.P5A13 -------------------- -------------------- -------------------- -------------------- --------------------
A19103.DAY_575.PlasmacDNA.P5A17 -------------------- -------------------- -------------------- -------------------- --------------------
A19103.DAY_575.PlasmacDNA.P5A21 -------------------- -------------------- -------------------- -------------------- --------------------
A19103.DAY_575.PlasmacDNA.P5B14 -------------------- -------------------- -------------------- -------------------- --------------------
A19103.DAY_575.PlasmacDNA.P5B19 -------------------- -------------------- -------------------- -------------------- M-------------------
 A19103.DAY_575.PlasmacDNA.P5C1 -------------------- -------------------- -------------------- -------------------- --------------------
 A19103.DAY_575.PlasmacDNA.P5C5 -------------------- -------------------- -------------------- -------------------- --------------------
 A19103.DAY_575.PlasmacDNA.P5C9 -------------------- -------------------- -------------------- -------------------- --------------------
A19103.DAY_575.PlasmacDNA.P5C12 -------------------- -------------------- -------------------- -------------------- -------T------------
A19103.DAY_575.PlasmacDNA.P5D21 -------------------- -------------------- -------------------- -------------------- --------------------
 A19103.DAY_575.PlasmacDNA.P5E8 -------------------- -------------------- -------------------- -------------------- --------------------
A19103.DAY_575.PlasmacDNA.P5E22 -------------------- -------------------- -------------------- -------------------- --------------------
A19103.DAY_575.PlasmacDNA.P5E23 -------------------- -------------------- -------------------- -------------------- --------------------
 A19103.DAY_575.PlasmacDNA.P5F2 -------------------- -------------------- -------------------- -------------------- --------------------
 A19103.DAY_575.PlasmacDNA.P5F3 -------------------- -------------------- -------------------- -------------------- --------------------
A19103.DAY_575.PlasmacDNA.P5F20 -------------------- -------------------- -------------------- -------------------- --------------------
 A19103.DAY_575.PlasmacDNA.P5G3 -------------------- -------------------- -------------------- -------------------- --------------------
 A19103.DAY_575.PlasmacDNA.P5G4 -------------------- -------------------- -------------------- -------------------- --------------------
A19103.DAY_575.PlasmacDNA.P5G11 -------------------- -------------------- -------------------- -------------------- --------------------
A19103.DAY_650.PlasmacDNA.P3J11 -------------------- -------------------- -------------------- -------------------- --------------------
A19103.DAY_650.PlasmacDNA.P4D15 -------------------- -------------------- -------------------- -------------------- --------------------
A19103.DAY_650.PlasmacDNA.P4D20 -------------------- -------------------- -------------------- -------------------- --------------------
```

```
 A19103.DAY_650.PlasmacDNA.P4E9 -------------------- -------------------- -------------------- -------------------- --------------------
A19103.DAY_650.PlasmacDNA.P4E21 -------------------- -------------------- -------------------- -------------------- --------------------
 A19103.DAY_650.PlasmacDNA.P4F2 -------------------- -------------------- -------------------- -------------------- --------------------
 A19103.DAY_650.PlasmacDNA.P4F8 -------------------- -------------------- -------------------- -------------------- --------------------
 A19103.DAY_650.PlasmacDNA.P4F9 -------------------- -------------------- -------------------- -------------------- --------------------
A19103.DAY_650.PlasmacDNA.P4G23 -------------------- -------------------- -------------------- -------------------- --------------------

                       SHIV1157 SPLSFQTHLPLPRGADRPEG IEEEGGERDRDRSIRLVTGS LALIWDDLRSLCLFSYHRLR DLLLIVTRTVELLGRRGWEA LKYWWNLLLYWSQELKNSAV  800
A19103.DAY_575.PlasmacDNA.P2M11 -------------------- -------------------- -------------------- -------------------- --------------------
A19103.DAY_575.PlasmacDNA.P2M13 -------------------- -------------------- -------------------- -------------------- --------------------
A19103.DAY_575.PlasmacDNA.P2M16 -------------------- -------------------- -------------------- -------------------- --------------------
A19103.DAY_575.PlasmacDNA.P2M18 -------------------- -------------------- -------------------- -------------------- --------------------
 A19103.DAY_575.PlasmacDNA.P2N4 -------------------- -------------------- -------------------- -------------------- --------------------
A19103.DAY_575.PlasmacDNA.P2N11 -------------------- -------------------- -------------------- -------------------- --------------------
A19103.DAY_575.PlasmacDNA.P2N19 -------------------- -------------------- -------------------- -------------------- --------------------
 A19103.DAY_575.PlasmacDNA.P5A9 -------------------- -------------------- -------------------- -------------------- --------------------
A19103.DAY_575.PlasmacDNA.P5A13 -------------------- -------------------- -------------------- ----------K--------- --------------------
A19103.DAY_575.PlasmacDNA.P5A17 -------------------- -------------------- -------------------- -------------------- --------------------
A19103.DAY_575.PlasmacDNA.P5A21 -------------------- -------------------- -------------------- -------------------- --------------------
A19103.DAY_575.PlasmacDNA.P5B14 -------------------- -------------------- -------------------- -------------------- --------------------
A19103.DAY_575.PlasmacDNA.P5B19 -------------------- -------------------- -------------------- -------------------- --------------------
 A19103.DAY_575.PlasmacDNA.P5C1 -------------------- -------------------- -------------------- -------------------- --------------------
 A19103.DAY_575.PlasmacDNA.P5C5 -------------------- -------------------- -------------------- -------------------- --------------------
 A19103.DAY_575.PlasmacDNA.P5C9 -------------------- -------------------- -------------------- -------------------- --------------------
A19103.DAY_575.PlasmacDNA.P5C12 -------------------- -------------------- -------------------- -------------------- --------------------
A19103.DAY_575.PlasmacDNA.P5D21 -------------------- -------------------- -------------------- -------------------- --------------------
 A19103.DAY_575.PlasmacDNA.P5E8 -------------------- -------------------- -------------------- -------------------- --------------------
A19103.DAY_575.PlasmacDNA.P5E22 -------------------- -------------------- -------------------- -------------------- --------------------
A19103.DAY_575.PlasmacDNA.P5E23 -------------------- -------------------- -------------------- -------------------- --------------------
 A19103.DAY_575.PlasmacDNA.P5F2 -------------------- -------------V------ -------------------- -------------------- --------------------
 A19103.DAY_575.PlasmacDNA.P5F3 -------------------- -------------------- -------------------- -------------------- --------------------
A19103.DAY_575.PlasmacDNA.P5F20 -------------------- -------------------- -------------------- -------------------- --------------------
 A19103.DAY_575.PlasmacDNA.P5G3 -------------------- -------------------- -------------------- -------------------- --------------------
 A19103.DAY_575.PlasmacDNA.P5G4 -------------------- -------------------- -------------------- -------------------- --------------------
A19103.DAY_575.PlasmacDNA.P5G11 -------------------- -------------------- -------------------- -------------------- --------------------
A19103.DAY_650.PlasmacDNA.P3J11 -------------------- ------D------------- -------------------- -------------------- --------------------
A19103.DAY_650.PlasmacDNA.P4D15 -------------------- ------D------------- -------------------- -------------------- --------------------
A19103.DAY_650.PlasmacDNA.P4D20 -------------------- ------D------------- -------------------- -------------------- --------------------
 A19103.DAY_650.PlasmacDNA.P4E9 -------------------- ------D------------- -------------------- -------------------- --------------------
A19103.DAY_650.PlasmacDNA.P4E21 -------------------- ------D------------- -------------------- -------------------- --------------------
 A19103.DAY_650.PlasmacDNA.P4F2 -------------------- ------D------------- -------------------- -------------------- --------------------
 A19103.DAY_650.PlasmacDNA.P4F8 -------------------- -------------------- -------------------- -------------------- --------------------
 A19103.DAY_650.PlasmacDNA.P4F9 -------------------- -------------------- -------------------- -------------------- --------------------
A19103.DAY_650.PlasmacDNA.P4G23 -------------------- -------------------- -------------------- -------------------- --------------------

                       SHIV1157 SLLNATAIAVRQYGWSYFHE AVQAVWRSATETLAGAWGDL WEILRRGGRWILAIPRRIRQ GLELTLL*  868
A19103.DAY_575.PlasmacDNA.P2M11 -------------------- -------------------- -------------------- --------
A19103.DAY_575.PlasmacDNA.P2M13 -------------------- -------------------- -------------------- --------
A19103.DAY_575.PlasmacDNA.P2M16 -------------------- -------------------- -------------------- --------
A19103.DAY_575.PlasmacDNA.P2M18 -------------------- -------------------- -------------------- --------
 A19103.DAY_575.PlasmacDNA.P2N4 -------------------- -------------------- -------------------- --------
A19103.DAY_575.PlasmacDNA.P2N11 -------------------- -------------------- -------------------- --------
A19103.DAY_575.PlasmacDNA.P2N19 -------------------- -------------------- -------------------- --------
 A19103.DAY_575.PlasmacDNA.P5A9 -------------------- -------------------- -------------------- --------
A19103.DAY_575.PlasmacDNA.P5A13 -------------------- -------------------- -------------------- --------
A19103.DAY_575.PlasmacDNA.P5A17 -------------------- -------------------- -------------------- --------
A19103.DAY_575.PlasmacDNA.P5A21 -------------------- -------------------- -------------------- --------
A19103.DAY_575.PlasmacDNA.P5B14 -------------------- -------------------- -------------------- --------
A19103.DAY_575.PlasmacDNA.P5B19 -------------------- -------------------- -------------------- --------
 A19103.DAY_575.PlasmacDNA.P5C1 -------------------- -------------------- -------------------- --------
 A19103.DAY_575.PlasmacDNA.P5C5 -------------------- -------------------- -------------------- --------
 A19103.DAY_575.PlasmacDNA.P5C9 -------------------- -------------------- -------------------- --------
A19103.DAY_575.PlasmacDNA.P5C12 -------------------- -------------------- -------------------- --------
A19103.DAY_575.PlasmacDNA.P5D21 -------------------- -------------------- -------------------- --------
 A19103.DAY_575.PlasmacDNA.P5E8 -------------------- -------------------- -------------------- --------
A19103.DAY_575.PlasmacDNA.P5E22 -------------------- -------------------- -------------------- --------
A19103.DAY_575.PlasmacDNA.P5E23 -------------------- -------------------- -------------------- --------
 A19103.DAY_575.PlasmacDNA.P5F2 -------------------- -------------------- -------------------- --------
 A19103.DAY_575.PlasmacDNA.P5F3 -------------------- -------------------- -------------------- --------
A19103.DAY_575.PlasmacDNA.P5F20 -------------------- -------------------- -------------------- --------
 A19103.DAY_575.PlasmacDNA.P5G3 -------------------- -------------------- -------------------- --------
 A19103.DAY_575.PlasmacDNA.P5G4 -------------------- -------------------- -------------------- --------
A19103.DAY_575.PlasmacDNA.P5G11 -------------------- -------------------- -------------------- --------
A19103.DAY_650.PlasmacDNA.P3J11 -------------------- ------K------------- -------------------- --------
A19103.DAY_650.PlasmacDNA.P4D15 -------------------- ------K------------- -------------------- --------
A19103.DAY_650.PlasmacDNA.P4D20 -------------------- ------K------------- -------------------- --------
 A19103.DAY_650.PlasmacDNA.P4E9 -------------------- ------K------------- -------------------- --------
A19103.DAY_650.PlasmacDNA.P4E21 -------------------- ------K------------- -------------------- --------
 A19103.DAY_650.PlasmacDNA.P4F2 -------------------- ------K------------- -------------------- --------
 A19103.DAY_650.PlasmacDNA.P4F8 -------------------- -------------------- -------------------- --------
 A19103.DAY_650.PlasmacDNA.P4F9 -------------------- -------------------- -------------------- --------
A19103.DAY_650.PlasmacDNA.P4G23 -------------------- -------------------- -------------------- --------
```

A19104 eCD4-Ig+Ciapavir

```
                       SHIV1157 MRVKEKYQHLWRWGWRWGIM LLGMLMICSASEKLWVTVYY GVPVWKEAKTTLFCASNAKA YEKEVHNIWATHACVPTDPN PQEIVLGNVTENFNMWKNDM  100
A19104.DAY_573.PlasmacDNA.P2J14 -------------------- -------------------- -------------------- -------------------- --------------------
A19104.DAY_573.PlasmacDNA.P2J18 -------------------- -------------------- -------------------- -------------------- --------------------
A19104.DAY_573.PlasmacDNA.P2J24 -------------------- -------------------- -------------------- -------------------- --------------------
A19104.DAY_650.PlasmacDNA.P3G18 -------------------- -------------------- -------------------- -------------------- --------------------
A19104.DAY_650.PlasmacDNA.P3H11 -------------------- -------------------- -------------------- -------------------- --------------------
 A19104.DAY_650.PlasmacDNA.P4I3 -------------------- -------------------- -------------------- -------------------- --------------------
 A19104.DAY_650.PlasmacDNA.P4I9 -------------------- -------------------- -------------------- -------------------- --------------------
A19104.DAY_650.PlasmacDNA.P4I22 -------------------- -------------------- -------------------- -------------------- --------------------
A19104.DAY_650.PlasmacDNA.P4I24 -------------------- -------------------- -------------------- -------------------- --------------------
 A19104.DAY_650.PlasmacDNA.P4J6 -------------------- -------------------- -------------------- -------------------- --------------------
 A19104.DAY_650.PlasmacDNA.P4K4 -------------------- -------------------- -------------------- -------------------- --------------------
 A19104.DAY_650.PlasmacDNA.P4K9 -------------------- -------------------- -------------------- -------------------- --------------------
 A19104.DAY_650.PlasmacDNA.P4L1 -------------------- -------------------- -------------------- -------------------- --------------------
 A19104.DAY_650.PlasmacDNA.P4L8 -------------------- -------------------- -------------------- -------------------- --------------------
A19104.DAY_650.PlasmacDNA.P4L20 -------------------- -------------------- -------------------- -------------------- --------------------
A19104.DAY_650.PlasmacDNA.P4M13 -------------------- -------------------- -------------------- -------------------- --------------------
A19104.DAY_650.PlasmacDNA.P4M18 -------------------- -------------------- -------------------- -------------------- --------------------
 A19104.DAY_650.PlasmacDNA.P4N8 -------------------- -------------------- -------------------- -------------------- --------------------
A19104.DAY_650.PlasmacDNA.P4N10 -------------------- -------------------- -------------------- -------------------- --------------------
A19104.DAY_650.PlasmacDNA.P4N12 -------------------- -------------------- -------------------- -------------------- --------------------
```

```
A19104.DAY_650.PlasmacDNA.P4N14 -------------------- -------------------- -------------------- -------------------- --------------------
A19104.DAY_650.PlasmacDNA.P4N22 -------------------- -------------------- -------------------- -------------------- --------------------
 A19104.DAY_650.PlasmacDNA.P4O3 -------------------- -------------------- -------------------- -------------------- --------------------
 A19104.DAY_650.PlasmacDNA.P4O7 -------------------- -------------------- -------------------- -------------------- --------------------
A19104.DAY_650.PlasmacDNA.P4O10 -------------------- -------------------- -------------------- -------------------- --------------------
A19104.DAY_650.PlasmacDNA.P4O11 -------------------- -------------------- -------------------- -------------------- --------------------
A19104.DAY_650.PlasmacDNA.P4P14 -------------------- -------------------- -------------------- -------------------- --------------------
A19104.DAY_650.PlasmacDNA.P4P16 -------------------- -------------------- -------------------- -------------------- --------------------
A19104.DAY_650.PlasmacDNA.P4P19 -------------------- -------------------- -------------------- -------------------- --------------------

                       SHIV1157 VDQMHEDIISLWDQSLKPCV KLTSLCVTLKCSNFTGKSNV TYKGDMEVKNCSFNVTTEIR DKKQKVYALFYRLDITPLDD NSSEYILINCNSSTITQACP  200
A19104.DAY_573.PlasmacDNA.P2J14 -------------------- -------------------- -------------------- -------------------- --------------------
A19104.DAY_573.PlasmacDNA.P2J18 -------------------- -------------------- -------------------- -------------------- --------------------
A19104.DAY_573.PlasmacDNA.P2J24 -------------------- -------------------- -------------------- -------------------- --------------------
A19104.DAY_650.PlasmacDNA.P3G18 -------------------- -------------------- -------------------- -------------------- --------------------
A19104.DAY_650.PlasmacDNA.P3H11 -------------------- -------------------- -------------------- -------------------- --------------------
 A19104.DAY_650.PlasmacDNA.P4I3 -------------------- -------------------- -------------------- -------------------- --------------------
 A19104.DAY_650.PlasmacDNA.P4I9 -------------------- -------------------- -------------------- -------------------- --------------------
A19104.DAY_650.PlasmacDNA.P4I22 -------------------- -------------------- -------------------- -------------------- --------------------
A19104.DAY_650.PlasmacDNA.P4I24 -------------------- -------------------- -------------------- -------------------- --------------------
 A19104.DAY_650.PlasmacDNA.P4J6 -------------------- -------------------- -------------------- -------------------- --------------------
 A19104.DAY_650.PlasmacDNA.P4K4 -------------------- -------------------- -------------------- -------------------- --------------------
 A19104.DAY_650.PlasmacDNA.P4K9 -------------------- -------------------- -------------------- -------------------- --------------------
 A19104.DAY_650.PlasmacDNA.P4L1 -------------------- -------------------- -------------------- -------------------- --------------------
 A19104.DAY_650.PlasmacDNA.P4L8 -------------------- -------------------- -------------------- -------------------- --------------------
A19104.DAY_650.PlasmacDNA.P4L20 -------------------- -------------------- -------------------- -------------------- --------------------
A19104.DAY_650.PlasmacDNA.P4M13 -------------------- -------------------- -------------------- -------------------- --------------------
A19104.DAY_650.PlasmacDNA.P4M18 -------------------- -------------------- -------------------- -------------------- --------------------
 A19104.DAY_650.PlasmacDNA.P4N8 -------------------- -------------------- -------------------- -------------------- --------------------
A19104.DAY_650.PlasmacDNA.P4N10 -------------------- -------------------- -------------------- ---R---------------- --------------------
A19104.DAY_650.PlasmacDNA.P4N12 -------------------- -------------------- -------------------- -------------------- --------------------
A19104.DAY_650.PlasmacDNA.P4N14 -------------------- -------------------- -------------------- -------------------- --------------------
A19104.DAY_650.PlasmacDNA.P4N22 -------------------- -------------------- -------------------- -------------------- --------------------
 A19104.DAY_650.PlasmacDNA.P4O3 -------------------- -------------------- -------------------- -------------------- --------------------
 A19104.DAY_650.PlasmacDNA.P4O7 -------------------- -----------------G-- -------------------- -------------------- --------------------
A19104.DAY_650.PlasmacDNA.P4O10 -------------------- -------------------- -------------------- -------------------- --------------------
A19104.DAY_650.PlasmacDNA.P4O11 -------------------- -------------------- -------------------- -------------------- --------------------
A19104.DAY_650.PlasmacDNA.P4P14 -------------------- -------------------- -------------------- -------------------- --------------------
A19104.DAY_650.PlasmacDNA.P4P16 -------------------- -------------------- -------------------- -------------------- --------------------
A19104.DAY_650.PlasmacDNA.P4P19 -------------------- -------------------- -------------------- -----------K-------- --------------------

                       SHIV1157 KVNFDPIPIHYCAPAGYAIL KCNNKTFNGTGPCHNVSTVQ CTHGIKPVVSTQLLLNGSLA EGEIIIRSENLTDNVKTIIV HFNESVEITCTRPNNNTRKS  300
A19104.DAY_573.PlasmacDNA.P2J14 -------------------- -------------------- -------------------- -------------------- --------------------
A19104.DAY_573.PlasmacDNA.P2J18 -------------------- -------------------- -------------------- -------------------- --------------------
A19104.DAY_573.PlasmacDNA.P2J24 -------------------- -------------------- -------------------- -------------------- --------------------
A19104.DAY_650.PlasmacDNA.P3G18 -------------------- -------------------- -------------------- -------------------- --------------------
A19104.DAY_650.PlasmacDNA.P3H11 -------------------- -------------------- -------------------- -------------------- --------------------
 A19104.DAY_650.PlasmacDNA.P4I3 -------------------- -------------------- -------------------- -------------------- --------------------
 A19104.DAY_650.PlasmacDNA.P4I9 -------------------- -------------------- -------------------- -------------------- --------------------
A19104.DAY_650.PlasmacDNA.P4I22 -------------------- -------------------- -------------------- -------------------- --------------------
A19104.DAY_650.PlasmacDNA.P4I24 -------------------- -------------------- -------------------- -------------------- --------------------
 A19104.DAY_650.PlasmacDNA.P4J6 -------------------- -------------------- -------------------- -------------------- --------------------
 A19104.DAY_650.PlasmacDNA.P4K4 -------------------- -------------------- -------------------- -------------------- --------------------
 A19104.DAY_650.PlasmacDNA.P4K9 -------------------- -------------------- -------------------- -------------------- --------------------
 A19104.DAY_650.PlasmacDNA.P4L1 -------------------- -------------------- -------------------- -------------------- --------------------
 A19104.DAY_650.PlasmacDNA.P4L8 -------------------- -------------------- -------------------- -------------------- --------------------
A19104.DAY_650.PlasmacDNA.P4L20 -------------------- -------------------- -------------------- -------------------- --------------------
A19104.DAY_650.PlasmacDNA.P4M13 -------------------- -------------------- -------------------- -------------------- --------------------
A19104.DAY_650.PlasmacDNA.P4M18 -------------------- -------------------- -------------------- -------------------- --------------------
 A19104.DAY_650.PlasmacDNA.P4N8 -------------------- -------------------- -------------------- -------------------- --------------------
A19104.DAY_650.PlasmacDNA.P4N10 -------------------- -------------------- -------------------- -------------------- --------------------
A19104.DAY_650.PlasmacDNA.P4N12 -------------------- -------------------- -------------------- -------------------- --------------------
A19104.DAY_650.PlasmacDNA.P4N14 -------------------- -------------------- -------------------- -------------------- --------------------
A19104.DAY_650.PlasmacDNA.P4N22 -------------------- -------------------- -------------------- -------------------- --------------------
 A19104.DAY_650.PlasmacDNA.P4O3 -------------------- -------------------- -------------------- -------------------- --------------------
 A19104.DAY_650.PlasmacDNA.P4O7 -------------------- -------------------- -------------------- -------------------- --------------------
A19104.DAY_650.PlasmacDNA.P4O10 -------------------- -------------------- -------------------- -------------------- --------------------
A19104.DAY_650.PlasmacDNA.P4O11 -------------------- -------------------- -------------------- -------------------- --------------------
A19104.DAY_650.PlasmacDNA.P4P14 -------------------- -------------------- -------------------- -------------------- --------------------
A19104.DAY_650.PlasmacDNA.P4P16 -------------------- -------------------- -------------------- -------------------- --------------------
A19104.DAY_650.PlasmacDNA.P4P19 -------------------- -------------------- -------------------- -------------------- --------------------

                       SHIV1157 ISIGPGQAIYATGDIIGDIR QAHCNISKENWNKTLQWVRG KLKEHFPNKTIVFKPSSGGD LEITTHSFNCRGEFFYCNTS KLFNSTDNSTHMGTENNTII  400
A19104.DAY_573.PlasmacDNA.P2J14 -------------------- -------------------- -------------------- -------------------- --------------------
A19104.DAY_573.PlasmacDNA.P2J18 ------R------------- -------------------- -------------------- -------------------- --------------------
A19104.DAY_573.PlasmacDNA.P2J24 -------------------- -------------------- -------------------- -------------------- --------------------
A19104.DAY_650.PlasmacDNA.P3G18 -------------------- -------------------- -------------------- -------------------- --------------------
A19104.DAY_650.PlasmacDNA.P3H11 -------------------- -------------------- -------------------- -------L------------ --------------------
 A19104.DAY_650.PlasmacDNA.P4I3 -------------------- -------------------- -------------------- -------------------- --------------------
 A19104.DAY_650.PlasmacDNA.P4I9 -------------------- -------------------- -------------------- -------------------- --------------------
A19104.DAY_650.PlasmacDNA.P4I22 -------------------- -------------------- -------------------- -------------------- --------------------
A19104.DAY_650.PlasmacDNA.P4I24 -------------------- -------------------- -------------------- -------------------- --------------------
 A19104.DAY_650.PlasmacDNA.P4J6 -------------------- -------------------- -------------------- -------------------- --------------------
 A19104.DAY_650.PlasmacDNA.P4K4 -------------------- -------------------- -------------------- -------------------- --------------------
 A19104.DAY_650.PlasmacDNA.P4K9 -------------------- -------------------- -------------------- -------------------- --------------------
 A19104.DAY_650.PlasmacDNA.P4L1 -------------------- -------------------- -------------------- -------------------- --------------------
 A19104.DAY_650.PlasmacDNA.P4L8 -------------------- -------------------- -------------------- -------------------- --------------------
A19104.DAY_650.PlasmacDNA.P4L20 -------------------- -------------------- -------------------- -------------------- --------------------
A19104.DAY_650.PlasmacDNA.P4M13 -------------------- -------------------- -------------------- -------------------- --------------------
A19104.DAY_650.PlasmacDNA.P4M18 -------------------- -------------------- -------------------- -------------------- --------------------
 A19104.DAY_650.PlasmacDNA.P4N8 -------------------- -------------------- -------------------- -------------------- --------------------
A19104.DAY_650.PlasmacDNA.P4N10 -------------------- -------------------- -------------------- -------------------- --------------------
A19104.DAY_650.PlasmacDNA.P4N12 -------------------- -------------------- -------------------- -------------------- --------------------
A19104.DAY_650.PlasmacDNA.P4N14 -------------------- -------------------- -------------------- -------------------- --------------------
A19104.DAY_650.PlasmacDNA.P4N22 -------------------- -------------------- -------------------- -------------------- --------------------
 A19104.DAY_650.PlasmacDNA.P4O3 -------------------- -------------------- -------------------- -------------------- --------------------
 A19104.DAY_650.PlasmacDNA.P4O7 -------------------- -------------------- -------------------- -------------------- --------------------
A19104.DAY_650.PlasmacDNA.P4O10 -------------------- -------------------- -------------------- -------------------- --------------------
A19104.DAY_650.PlasmacDNA.P4O11 -------------------- -------------------- -------------------- -------------------- --------------------
A19104.DAY_650.PlasmacDNA.P4P14 ---------C---------- -------------------- -------------------- -------------------- --------------------
A19104.DAY_650.PlasmacDNA.P4P16 -------------------- -------------------- -------------------- -------------------- --------------------
A19104.DAY_650.PlasmacDNA.P4P19 -------------------- -------------------- -------------------- -------------------- --------------------

                       SHIV1157 TIPCRIKQIINMWQEVGRAM YAPPIEGNITCKSNITGLLL VRDGGWDNSTNDTETFRPGG GDMRDNWRSELYKYKVVEVK PLGIAPTKAKRRVVEREKRA  500
A19104.DAY_573.PlasmacDNA.P2J14 -------------------- -------------------- -------------------- -------------------- --------------------
```

```
A19104.DAY_573.PlasmacDNA.P2J18 -------------------- -------------------- -------------------- -------------------- --------------------
A19104.DAY_573.PlasmacDNA.P2J24 -------------------- -------------------- -------------------- -------------------- --------------------
A19104.DAY_650.PlasmacDNA.P3G18 -------------------- -------------------- -------------------- -------------------- --------------------
A19104.DAY_650.PlasmacDNA.P3H11 -------------------- -------------------- -------------------- -------------------- --------------------
 A19104.DAY_650.PlasmacDNA.P4I3 -------------------- -------------------- -------------------- -------------------- --------------------
 A19104.DAY_650.PlasmacDNA.P4I9 -------------------- -------------------- -------------------- -------------------- --------------------
A19104.DAY_650.PlasmacDNA.P4I22 -------------------- -------------------- -------------------- -------------------- --------------------
A19104.DAY_650.PlasmacDNA.P4I24 -------------------- -------------------- -------------------- -------------------- --------------------
 A19104.DAY_650.PlasmacDNA.P4J6 -------------------- -------------------- -------------------- -------------------- --------------------
 A19104.DAY_650.PlasmacDNA.P4K4 -------------------- -------------------- -------------------- -------------------- --------------------
 A19104.DAY_650.PlasmacDNA.P4K9 -------------------- -------------------- -------------------- -------------------- --------------------
 A19104.DAY_650.PlasmacDNA.P4L1 -------------------- -------------------- -------------------- -------------------- --------------------
 A19104.DAY_650.PlasmacDNA.P4L8 -------------------- -------------------- -------------------- -------------------- --------------------
A19104.DAY_650.PlasmacDNA.P4L20 -------------------- -------------------- -------------------- -------------------- --------------------
A19104.DAY_650.PlasmacDNA.P4M13 -------------------- -------------------- -------------------- -------------------- --------------------
A19104.DAY_650.PlasmacDNA.P4M18 -------------------- -------------------- -------------------- -------------------- --------------------
 A19104.DAY_650.PlasmacDNA.P4N8 -------------------- -------------------- -------------------- -------------------- --------------------
A19104.DAY_650.PlasmacDNA.P4N10 -------------------- -------------------- -------------------- -------------------- --------------------
A19104.DAY_650.PlasmacDNA.P4N12 -------------------- -------------------- -------------------- -------------------- --------------------
A19104.DAY_650.PlasmacDNA.P4N14 -------------------- -------------------- -------------------- -------------------- --------------------
A19104.DAY_650.PlasmacDNA.P4N22 -------------------- -------------------- -------------------- -------------------- --------------------
 A19104.DAY_650.PlasmacDNA.P4O3 -------------------- -------------------- -------------------- -------------------- --------------------
 A19104.DAY_650.PlasmacDNA.P4O7 -------------------- -------------------- -------------------- -------------------- --------------------
A19104.DAY_650.PlasmacDNA.P4O10 -------------------- -------------------- -------------------- -------------------- --------------------
A19104.DAY_650.PlasmacDNA.P4O11 -------------------- -------------------- -------------------- -------------------- --------------------
A19104.DAY_650.PlasmacDNA.P4P14 -------------------- -------------------- -------------------- -------------------- --------------------
A19104.DAY_650.PlasmacDNA.P4P16 -------------------- -------------------- -------------------- -------------------- --------------------
A19104.DAY_650.PlasmacDNA.P4P19 -------------------- -------------------- -------------------- -------------------- --------------------

                       SHIV1157 VGIGAVFLGFLGAAGSTMGA ASITLTVQARQLLSGIVQQQ DNLLRAIEAQQHMLQLTVWG IKQLQARVLAIERYLQDQQL LGIWGCSGKLICTTAVPWND  600
A19104.DAY_573.PlasmacDNA.P2J14 -------------------- -------------------- -------------------- ----------V--------- --------------------
A19104.DAY_573.PlasmacDNA.P2J18 -------------------- -------------------- -------------------- -------------------- --------------------
A19104.DAY_573.PlasmacDNA.P2J24 -------------------- -------------------- -------------------- ----------V--------- --------------------
A19104.DAY_650.PlasmacDNA.P3G18 -------------------- -----------------R-- -------------------- -------------------- --------------------
A19104.DAY_650.PlasmacDNA.P3H11 -------------------- -------------------- -------------------- ----------V--------- --------------------
 A19104.DAY_650.PlasmacDNA.P4I3 -------------------- -------------------- -------------------- ----------V--------- --------------------
 A19104.DAY_650.PlasmacDNA.P4I9 -------------------- -------------------- -------------------- -------------------- --------------------
A19104.DAY_650.PlasmacDNA.P4I22 -------------------- -------------------- -------------------- -------------------- --------------------
A19104.DAY_650.PlasmacDNA.P4I24 -------------------- -------------------- -------------------- -------------------- --------------------
 A19104.DAY_650.PlasmacDNA.P4J6 -------------------- -------------------- -------------------- ----------V--------- --------------------
 A19104.DAY_650.PlasmacDNA.P4K4 -------------------- -------------------- -------------------- -------------------- --------------------
 A19104.DAY_650.PlasmacDNA.P4K9 -------------------- -------------------- -------------------- -------------------- --------------------
 A19104.DAY_650.PlasmacDNA.P4L1 -------------------- -------------------- -------------------- ----------V--------- --------------------
 A19104.DAY_650.PlasmacDNA.P4L8 -------------------- -------------------- -------------------- -------------------- --------------------
A19104.DAY_650.PlasmacDNA.P4L20 -------------------- -------------------- -------------------- ----------V--------- --------------------
A19104.DAY_650.PlasmacDNA.P4M13 -------------------- -------------------- -------------------- ----------V--------- --------------------
A19104.DAY_650.PlasmacDNA.P4M18 -------------------- -------------------- -------------------- -------------------- --------------------
 A19104.DAY_650.PlasmacDNA.P4N8 -------------------- -------------------- -------------------- ----------V--------- --------------------
A19104.DAY_650.PlasmacDNA.P4N10 -------------------- -------------------- -------------------- -------------------- --------------------
A19104.DAY_650.PlasmacDNA.P4N12 -------------------- -------------------- -------------------- ----------V--------- --------------------
A19104.DAY_650.PlasmacDNA.P4N14 -------------------- -------------------- -------------------- -------------------- --------------------
A19104.DAY_650.PlasmacDNA.P4N22 -------------------- -------------------- -------------------- ----------V--------- --------------------
 A19104.DAY_650.PlasmacDNA.P4O3 -------------------- -------------------- -------------------- ----------V--------- --------------------
 A19104.DAY_650.PlasmacDNA.P4O7 -------------------- -------------------- -------------------- -------------------- --------------------
A19104.DAY_650.PlasmacDNA.P4O10 -------------------- -------------------- -------------------- -------------------- --------------------
A19104.DAY_650.PlasmacDNA.P4O11 -------------------- -------------------- -------------------- ----------V--------- --------------------
A19104.DAY_650.PlasmacDNA.P4P14 -------------------- -------------------- -------------------- ----------V--------- --------------------
A19104.DAY_650.PlasmacDNA.P4P16 -------------------- -------------------- -------------------- -------------------- --------------------
A19104.DAY_650.PlasmacDNA.P4P19 -------------------- -------------------- -------------------- ----------V--------- --------------------

                       SHIV1157 SWSNKSQTDIWENMTWMQWD REISRHTDTIYRLLEDSQNQ QEKNEKDLLALDSWKNLWNW FSITRWLWYIKIFIMIVGGL IGLRIIFAVLSIVNRVRQGY  700
A19104.DAY_573.PlasmacDNA.P2J14 -------------------- -------------------- --E----------------- -------------------- --------------------
A19104.DAY_573.PlasmacDNA.P2J18 -------------------- -------------------- -------------------- -------------------- --------------------
A19104.DAY_573.PlasmacDNA.P2J24 -------------------- -------------------- --E----------------- -------------------- --------------------
A19104.DAY_650.PlasmacDNA.P3G18 -------------------- -------------------- -------------------- -------------------- M-------------------
A19104.DAY_650.PlasmacDNA.P3H11 -------------------- -------------------- -------------------- -------------------- --------------------
 A19104.DAY_650.PlasmacDNA.P4I3 -------------------- -------------------- -------------------- -------------------- --------------------
 A19104.DAY_650.PlasmacDNA.P4I9 -------------------- -------------------- ------------G------- -------------------- --------------------
A19104.DAY_650.PlasmacDNA.P4I22 -------------------- -------------------- -------------------- -------------------- --------------------
A19104.DAY_650.PlasmacDNA.P4I24 -------------------- -------------------- -------------------- -------------------- --------------------
 A19104.DAY_650.PlasmacDNA.P4J6 -------------------- -------------------- -------------------- -------------------- --------------------
 A19104.DAY_650.PlasmacDNA.P4K4 -------------------- -------------------- -------------------- -------------------- --------------------
 A19104.DAY_650.PlasmacDNA.P4K9 -------------------- -------------------- --E----------------- -------------------- --------------------
 A19104.DAY_650.PlasmacDNA.P4L1 -------------------- -------------------- -------------------- -------------------- -----------V--------
 A19104.DAY_650.PlasmacDNA.P4L8 -------------------- -------------------- -------------------- -------------------- M-------------------
A19104.DAY_650.PlasmacDNA.P4L20 -------------------- -------------------- -------------------- -------------------- -----------V--------
A19104.DAY_650.PlasmacDNA.P4M13 -------------------- -------------------- -------------------- -------------------- ---K----------------
A19104.DAY_650.PlasmacDNA.P4M18 -------------------- -------------------- -------------------- -------------------- --------------------
 A19104.DAY_650.PlasmacDNA.P4N8 -------------------- -------------------- -------------------- -------------------- --------------------
A19104.DAY_650.PlasmacDNA.P4N10 -------------------- -------------------- -------------------- -------------------- --------------------
A19104.DAY_650.PlasmacDNA.P4N12 -------------------- -------------------- -------------------- -------------------- --------------------
A19104.DAY_650.PlasmacDNA.P4N14 -------------------- -------------------- -------------------- -------------------- M-------------------
A19104.DAY_650.PlasmacDNA.P4N22 -------------------- -------------------- --E----------------- -------------------- --------------------
 A19104.DAY_650.PlasmacDNA.P4O3 -------------------- -------------------- -------------------- -------------------- --------------------
 A19104.DAY_650.PlasmacDNA.P4O7 -------------------- -------------------- -------------------- -------------------- M-------------------
A19104.DAY_650.PlasmacDNA.P4O10 -------------------- -------------------- -------------------- -------------------- --------------------
A19104.DAY_650.PlasmacDNA.P4O11 -------------------- -------------------- --E----------------- -------------------- --------------------
A19104.DAY_650.PlasmacDNA.P4P14 -------------------- -------------------- --E----------------- -------------------- --------------------
A19104.DAY_650.PlasmacDNA.P4P16 -------------------- -------------------- -------------------- -------------------- --------------------
A19104.DAY_650.PlasmacDNA.P4P19 -------------------- -------------------- -------------------- -------------------- -----------V--------

                       SHIV1157 SPLSFQTHLPLPRGADRPEG IEEEGGERDRDRSIRLVTGS LALIWDDLRSLCLFSYHRLR DLLLIVTRTVELLGRRGWEA LKYWWNLLLYWSQELKNSAV  800
A19104.DAY_573.PlasmacDNA.P2J14 -------------------- -------------------- -------------------- -------------------- --------------------
A19104.DAY_573.PlasmacDNA.P2J18 -------------------- -------------------- -------------------- -------------------- --------------------
A19104.DAY_573.PlasmacDNA.P2J24 -------------------- -------------------- -------------------- -------------------- --------------------
A19104.DAY_650.PlasmacDNA.P3G18 -------------------- -------------------- -------------------- -------------------- --------------------
A19104.DAY_650.PlasmacDNA.P3H11 -------------------- -------------------- -------------------- -------------------- --------------------
 A19104.DAY_650.PlasmacDNA.P4I3 -------------------- -------------------- -------------------- -------------------- --------------------
 A19104.DAY_650.PlasmacDNA.P4I9 -------------------- -------------------- -------------------- -------------------- --------------------
A19104.DAY_650.PlasmacDNA.P4I22 -------------------- -------------------- -------------------- -------------------- --------------------
A19104.DAY_650.PlasmacDNA.P4I24 -------------------- -------------------- -------------------- -------------------- --------------------
 A19104.DAY_650.PlasmacDNA.P4J6 -------------------- -------------------- -------------------- -------------------- --------------------
 A19104.DAY_650.PlasmacDNA.P4K4 -------------------- -------------------- -------------------- -------------------- --------------------
 A19104.DAY_650.PlasmacDNA.P4K9 -------------------- -------------------- -------------------- -------------------- --------------------
 A19104.DAY_650.PlasmacDNA.P4L1 -------------------- -------------------- -------------------- -------------------- --------------------
```

```
 A19104.DAY_650.PlasmacDNA.P4L8 -------------------- -------------------- -------------------- -------------------- -R------------------
A19104.DAY_650.PlasmacDNA.P4L20 -------------------- -------------------- -------------------- -------------------- --------------------
A19104.DAY_650.PlasmacDNA.P4M13 -------------------- -------------------- -------------------- -------------------- --------------------
A19104.DAY_650.PlasmacDNA.P4M18 -------------------- -------------------- -------------------- -------------------- --------------------
 A19104.DAY_650.PlasmacDNA.P4N8 -------------------- -------------------- -------------------- -------------------- --------------------
A19104.DAY_650.PlasmacDNA.P4N10 -------------------- -------------------- -------------------- -------------------- --------------------
A19104.DAY_650.PlasmacDNA.P4N12 -------------------- -------------------- -------------------- -------------------- --------------------
A19104.DAY_650.PlasmacDNA.P4N14 -------------------- -------------------- -------------------- -------------------- --------------------
A19104.DAY_650.PlasmacDNA.P4N22 -------------------- -------------------- -------------------- -------------------- --------------------
 A19104.DAY_650.PlasmacDNA.P4O3 -------------------- -------------------- -------------------- -------------------- --------------------
 A19104.DAY_650.PlasmacDNA.P4O7 -------------------- -------------V------ -------------------- -------------------- --------------------
A19104.DAY_650.PlasmacDNA.P4O10 -------------------- -------------------- -------------------- -------------------- --------------------
A19104.DAY_650.PlasmacDNA.P4O11 -------------------- -------------------- -------------------- -------------------- --------------------
A19104.DAY_650.PlasmacDNA.P4P14 -------------------- -------------------- -------------------- -------------------- --------------------
A19104.DAY_650.PlasmacDNA.P4P16 -------------------- -------------V------ -------------------- -------------------- --------------------
A19104.DAY_650.PlasmacDNA.P4P19 -------------------- -------------------- -------------------- -------------------- --------------------

                       SHIV1157 SLLNATAIAVRQYGWSYFHE AVQAVWRSATETLAGAWGDL WEILRRGGRWILAIPRRIRQ GLELTLL*  868
A19104.DAY_573.PlasmacDNA.P2J14 -------------------- -------------------- -------------------- --------
A19104.DAY_573.PlasmacDNA.P2J18 -------------------- -------------------- -------------------- --------
A19104.DAY_573.PlasmacDNA.P2J24 -------------------- -------------------- -------------------- --------
A19104.DAY_650.PlasmacDNA.P3G18 ----------G--------- -------------------- -------------------- --------
A19104.DAY_650.PlasmacDNA.P3H11 -------------------- -------------------- --T----------------- --------
 A19104.DAY_650.PlasmacDNA.P4I3 -------------------- -------------------- -------------------- --------
 A19104.DAY_650.PlasmacDNA.P4I9 ----------G--------- -------------------- -------------------- --------
A19104.DAY_650.PlasmacDNA.P4I22 -------------------- -------------------- -------------------- --------
A19104.DAY_650.PlasmacDNA.P4I24 -------------------- -------------------- -------------------- --------
 A19104.DAY_650.PlasmacDNA.P4J6 -------------------- -------------------- --T----------------- --------
 A19104.DAY_650.PlasmacDNA.P4K4 -------------------- -------------------- -------------------- --------
 A19104.DAY_650.PlasmacDNA.P4K9 -------------------- -------------------- --T----------------- --------
 A19104.DAY_650.PlasmacDNA.P4L1 -------------------- -------------------- -------------------- --------
 A19104.DAY_650.PlasmacDNA.P4L8 -------------------- -------------------- -------------------- --------
A19104.DAY_650.PlasmacDNA.P4L20 -------------------- -------------------- -------------------- --------
A19104.DAY_650.PlasmacDNA.P4M13 -------------------- -------------------- --T----------------- --------
A19104.DAY_650.PlasmacDNA.P4M18 -------------------- -------------------- -------------------- --------
 A19104.DAY_650.PlasmacDNA.P4N8 -------------------- -------------------- --T----------------- --------
A19104.DAY_650.PlasmacDNA.P4N10 -------------------- -------------------- ----------V--------- --------
A19104.DAY_650.PlasmacDNA.P4N12 -------------------- -------------------- -------------------- --------
A19104.DAY_650.PlasmacDNA.P4N14 -------------------- -------------------- -------------------- --------
A19104.DAY_650.PlasmacDNA.P4N22 -------------------- ----I--------------- --T----------------- --------
 A19104.DAY_650.PlasmacDNA.P4O3 -------------------- -------------------- --T----------------- --------
 A19104.DAY_650.PlasmacDNA.P4O7 -------------------- -------------------- -------------------- --------
A19104.DAY_650.PlasmacDNA.P4O10 -------------------- -------------------- -------------------- --------
A19104.DAY_650.PlasmacDNA.P4O11 -------------------- -------------------- -------------------- --------
A19104.DAY_650.PlasmacDNA.P4P14 -------------------- -------------------- -------------------- --------
A19104.DAY_650.PlasmacDNA.P4P16 -------------------- -------------------- -------------------- --------
A19104.DAY_650.PlasmacDNA.P4P19 -------------------- -------F------------ --T----------------- --------
```

Supplemental Table 2: Env sequencing data

Region	Name	Sequence	Company
GAG	SHIVGAG_Fwd	TTAAGTGTTGGAATTGTGGGAAAG	Thermo Fisher
	SHIVGAG_Rev	TTTCCAGCATCCCTGTCTTCTT	Thermo Fisher
	SHIVGAG_Probe	**[FAM**]CAATGCAGAGCCC-**MBG**	Thermo Fisher
POL	SHIVPOL_Fwd	AGGAAAAGAGGTGGATAGCAGTTC	Thermo Fisher
	SHIVPOL_Rev	TTTATGAGGCTATGCCACCTCTCT	Thermo Fisher
	SHIVPOL_Probe	[**ATTO590**]CACATGGAGGATACCGGAGA[**MGB-NFQ**]	IDT
LTR	SHIVLTR_Fwd	GCAGGTAAGTGCAACACAAAAAA	Thermo Fisher
	SHIVLTR_Rev	CGCCCATCTCCCACTCTATC	Thermo Fisher
	SHIVLTR_Probe	**[VIC]**TAGCTGTCTTTTATCCAGGAAG-**MGB**	Thermo Fisher
TAT	SHIVTAT_Fwd	GGCATCTCCTATGGCAGGAA	Thermo Fisher
	SHIVTAT_ Rev	AGTCTGACTGTTCTGATGAGCTCTTC	Thermo Fisher
	SHIVTAT_Probe	**[CY5.5]**AAGCGGAGACAGCG-**MGB**	IDT
ENV	SHIVENV_Fwd	AGCTGTGTTCCTTGGGTTCTT	Thermo Fisher
	SHIVENV_Rev	CTGTACCGTCAGCGTTATTGA	Thermo Fisher
	SHIVENV probe	**[ROX]**AGCAGCAGGAAGCA-**MGB**	IDT
MRPP30	MRPP30 3'_Fwd	GTTCTTCTTAGCCTCTTCGTAATTCC	Thermo Fisher
	MRPP30 3'_Rev	CCACCCACAAGGAGAAGAAAAT	Thermo Fisher
	MRPP30 3'_Probe	**[FAM]**ATGTAAAGTCAGCTTCCCCACAGCCAAGA**-BHQ**	Thermo Fisher
MRPP30	MRPP30 5'_Fwd	CAGGTCATTATTGCTCCCTCTTCT	Thermo Fisher
	MRPP30 5'_Rev	CAAAACAACATCAGTGGCACGTA	Thermo Fisher
	MRPP30 5'_Probe	[**VIC**]TGCTCTTCCTTACTCTGCCCTTTCCCC-**BHQ**	Thermo Fisher
MDelta D	MDeltaD_Fwd	ATGGGAATGTGTAACTTTGTGAAGAT	Thermo Fisher
	MDeltaD_Rev	TAATGGCTTGATAAAGATGAGTGATCA	Thermo Fisher
	MDeltaD_Probe	[**ROX**]TCTGTGGCCATCTTA[**MGB-BHQ]**	IDT

Supplemental Table 3: Intact proviral DNA assay (IPDA) primer/probe sequences

Supplemental References

1. O'Brien SP, Swanstrom AE, Pegu A, Ko SY, Immonen TT, Del Prete GQ, et al. Rational design and in vivo selection of SHIVs encoding transmitted/founder subtype C HIV-1 envelopes. *PLoS Pathog.* 2019;15(4):e1007632.
2. Keele BF, Giorgi EE, Salazar-Gonzalez JF, Decker JM, Pham KT, Salazar MG, et al. Identification and characterization of transmitted and early founder virus envelopes in primary HIV-1 infection. *Proc Natl Acad Sci U S A.* 2008;105(21):7552-7.
3. Keele BF, Li H, Learn GH, Hraber P, Giorgi EE, Grayson T, et al. Low-dose rectal inoculation of rhesus macaques by SIVsmE660 or SIVmac251 recapitulates human mucosal infection by HIV-1. *J Exp Med.* 2009;206(5):1117-34.
4. Wiegand A, Spindler J, Hong FF, Shao W, Cyktor JC, Cillo AR, et al. Single-cell analysis of HIV-1 transcriptional activity reveals expression of proviruses in expanded clones during ART. *Proc Natl Acad Sci U S A.* 2017;114(18):E3659-E68.
5. Levy CN, Hughes SM, Roychoudhury P, Reeves DB, Amstuz C, Zhu H, et al. A highly multiplexed droplet digital PCR assay to measure the intact HIV-1 proviral reservoir. *Cell Rep Med.* 2021;2(4):100243.
6. Green MR, and Sambrook J. Precipitation of DNA with Ethanol. *Cold Spring Harb Protoc.* 2016;2016(12).
7. Nixon CC, Mavigner M, Sampey GC, Brooks AD, Spagnuolo RA, Irlbeck DM, et al. Systemic HIV and SIV latency reversal via non-canonical NF-kappaB signalling in vivo. *Nature.* 2020;578(7793):160-5.
